# Statistics of Language Morphology Change: From Biconsonantal Hunters to Triconsonantal Farmers

**DOI:** 10.1371/journal.pone.0083780

**Published:** 2013-12-19

**Authors:** Noam Agmon, Yigal Bloch

**Affiliations:** 1 Institute of Chemistry, The Hebrew University of Jerusalem, Jerusalem, Israel; 2 Department of Jewish History, The Hebrew University of Jerusalem, Jerusalem, Israel; University of Leicester, United Kingdom

## Abstract

Linguistic evolution mirrors cultural evolution, of which one of the most decisive steps was the "agricultural revolution" that occurred 11,000 years ago in W. Asia. Traditional comparative historical linguistics becomes inaccurate for time depths greater than, say, 10 kyr. Therefore it is difficult to determine whether decisive events in human prehistory have had an observable impact on human language. Here we supplement the traditional methodology with independent statistical measures showing that following the transition to agriculture, languages of W. Asia underwent a transition from biconsonantal (2c) to triconsonantal (3c) morphology. Two independent proofs for this are provided. Firstly the reconstructed Proto-Semitic fire and hunting lexicons are predominantly 2c, whereas the farming lexicon is almost exclusively 3c in structure. Secondly, while Biblical verbs show the usual Zipf exponent of about 1, their 2c subset exhibits a larger exponent. After the 2c > 3c transition, this could arise from a faster decay in the frequency of use of the less common 2c verbs. Using an established frequency-dependent word replacement rate, we calculate that the observed increase in the Zipf exponent has occurred over the 7,500 years predating Biblical Hebrew namely, starting with the transition to agriculture.

## Introduction

 In most of its history, *homo-sapiens sapiens* followed the hunter-gatherer way of life. Between 15,000 and 10,000 years ago, a major transition in human sustenance was instigated in W. Asia, which set the stage for modern human society: the transition to agriculture [1]. This included domestication of plants and mammals [2], sedentism and the establishment of the large Neolithic villages [3]. An important factor allowing the concomitant enhancement in social complexity was the ability to communicate. Was language influenced by this decisive step in human prehistory? Most of the historical linguistic literature does not explicitly relate to this intriguing question. 

 In Semitic languages [4], a hypothetical transition from biconsonsonantal (2c) to triconsonantal (3c) language morphology was debated for quite some time [5]. Semitic lexemes are derived from roots consisting of predominantly three radicals (i.e., root consonants), termed 3c. However, there is a small corpus of 2c roots (defined in Methods), responsible for most of the irregular Semitic verbs. Are these remnants from a more archaic linguistic phase? One observation favoring this is the relative abundance of 2c body parts and, particularly facial features (“eye”, “tooth”, etc.). If this semantic field originated early in language development then so did the 2c morphology. But how can we know this? 

 Further progress can be made by correlating linguistic and archeological innovations. Selecting an archeologically dateable semantic field (e.g., materials), we have shown [6] that, in the reconstructed Proto-Semitic (PS) language [7,8], names of materials known to and utilized by early hunter-gatherers (wood, reed, stone, flint, lime, gravel, sand, mud, clay, cloth, skin and water) are overwhelmingly (85%) of 2c morphology, while materials introduced as of the Neolithic period in W. Asia (bitumen, sulfur, salt, charcoal, pottery, brick, wool, lead, antimony, copper, silver and gold) were all given 3c names. This non-uniform distribution of 2c vs. 3c lexemes in these two semantic fields suggests that a 2c > 3c language morphology change accompanied the transition to agriculture in the Early Neolithic, ca. 11,000 years Before Present (BP). 

 Such a dramatic event in the prehistory of pre-Semitic languages, if occurred, must have impacted the statistics of 2c vs. 3c lexemes in Semitic languages. The present work explores two independent consequences of such an irreversible language replacement process that together provide a rather compelling evidence for its occurrence. The first makes use of comparative linguistics and archeology, whereas the second uses lexical statistics.

 Firstly, there should be a rift between the lexicon of farmers and their predecessor hunter-gatherers. One can nowadays reconstruct PS rather reliably [8] thanks to the extensive Akkadian (Akk.) texts [9], which go back 2.5–4.5 kyr. PS was supposedly spoken during the Chalcolithic period, sometime between 5,750 BP [10] and 6,300 BP [11]. The society then was already composed of well-established agricultural communities, whose language must have contained the linguistic innovations of the agricultural era side-by-side with relics from the hunter-gatherer lexicon, prevailing just 5 kyr earlier. We therefore reconstruct the hunter-gatherer and farmer lexicons at the PS level, focusing predominantly on archeologically dateable human innovations. The reconstructions are justified in the Etymological Appendix (EA) that includes Text S1, Tables S1-S4, and Text S2 within the Information (the complete EA is linked as Text S3).

 Just as in the case of materials, we expect the hunter-gatherer lexicon to be enriched in 2c lexemes (bearing in mind that some of these have already been replaced by new 3c terms), whereas the farmer's lexicon should have 3c morphology. Additionally, a sizeable percentage of the PS hunter's lexicon should appear in Proto Afroasiatic (PAA) [11-18], the predecessor of PS, or even in the reconstructed lexicon of the Nostratic macrofamily [19-22].

 The second approach utilizes word frequency analysis [23], starting with Zipf, who showed that when words in a given text are ranked (r) by their frequency (f) of utilization, a power-law is observed [24]: 

(1) $$f=Ar^{\alpha }$$

The Zipf exponent α is about 1 in natural languages, while *A* is a normalizing factor that depends on the total size of the textual corpus [23]. A similar correlation holds just for the verbs extracted from a given text [25]. Eq. (1) fits most word frequency data, except for the highest ranks (which may be text-specific), and the low-frequency (large *r*) part that deviates downward from this correlation. This may represent a switch-over from *α*_0_≈1at small *r* to *α*_1_≈2 at large *r* [26,27]. The high frequency words obeying the original Zipf law constitute a "kernel lexicon", whereas the vast low frequency part consists of more specific terminology.

 Word frequency is intimately connected to language history. It was already noted e.g., in Chap. 3 of Zipf's book [24], that high-frequency words tend to be older. This was recently quantified via a decay rate coefficient, *k*(*f*), that is larger for less frequently used words [28,29]. If words only disappear, with a monotonically decreasing *k*(*f*), their Zipf exponent would increase with time. The fact that α is always about 1 suggests that new words are formed with a similar *k*(*f*), and these balance the death of the old ones. 

 It follows that for any non-productive, morphologically distinguishable lexical subset (consisting of word types that stopped being created), α should increase with time. Here we analyze the frequency of verbal roots in an ancient Semitic text, the Hebrew Bible. We find that while the total verbal corpus shows the expected Zipf behavior, the 2c verbal roots (Table S5) exhibit a noticeably larger Zipf exponent. Using the power-law form of *k*(*f*) determined by Pagel et al. [29], the increase in α is uniquely converted to a lifetime for the 2c corpus. We find that the end of the 2c era has occurred ca. 7.8 kyr before Biblical Hebrew (BH) [30-32], and this corresponds rather nicely to the onset of agriculture.

## Methods

 Reconstruction of PS is rather straightforward [8] and less controversial than reconstructions on deeper levels, such as PAA [12-18] and Nostratic [19-21]. PS is believed to be based on the 29 consonant phonemes in the transliteration table S6 (one more than in Arabic). Every reconstructed PS word must normally have reflexes ("cognates") in Akk. [9] and at least one W. Semitic language. Relaxing the demand for an Akk. cognate will inevitably lead to additional (more questionable) reconstructions (see *DAE*). Borrowing is excluded based on expert opinions from the linguistic literature. Details and discussions of all reconstructions are given in the EA compiled by Yigal Bloch (Text S3), which is in general agreement with earlier work [8].

 Next, we suggest two definitions of 2c roots. The narrow definition includes only strictly 2c nouns, such as *da**m** “blood” (note the embolden radicals), the "hollow" II-*w* roots (that in the traditional 3c grammar have *w* or *y* as the second radical), and those with a reduplicated last consonant. By direct counting in a Biblical Concordance we find that this group corresponds to about 12% of BH nouns. These possibly originate from an early phase of the 2c language. 

 The broad definition of 2c roots includes, in addition, all I-*n* and I-*w* plus most I-*y* and III-*w/y* roots [4,6,32]. These added consonants may represent early affixes, later perceived as radicals. We estimate that roughly 35% of BH nouns are 2c (and 60% 3c) according to this criterion. This broader 2c lexicon should correspond to the latest phase of the 2c language, just before the transition to 3c morphology occurred. In the statistical analysis of 2c BH verbs below we include, with little loss of accuracy, all I-*y* and III-*w/y* roots (Table S5), and this allows for a nearly automated procedure for 2c verb identification. 

## Results

### I: The hunter-gatherer lexicon

 Our hunter-gatherer lexicon avoids terms characteristic of both hunter and farmer cultures (e.g., “to collect” may refer to collecting produce from the wild or an agricultural field). We avoid most plant and animal names that could have been introduced either before or after their domestication. This leaves mainly terms related to fire and hunting, whose inclusion in the lexicon is justified by archeological data discussed below. *A priori* one might expect a similar 3c/2c ratio in all semantic fields. But, as we show below, this is not the case.

####  (a) Fire

"The manufacture of stone tools and the manipulation of fire are the most important extrasomatic milestones in our early evolutionary trajectory" [33]. "Fire played a multifunctional role in human history: a source of warmth, light, and a means for cooking; it could also serve to discourage carnivores, clear areas of vegetation, and be used for the smoking and drying of meat, among others" [34]. Evidence for hominin use of fire may go back 790,000 years [35]. 

 The multifunctional role of fire is manifested by several PS synonyms for the noun “fire” (Table 1). We can use this abundance as a statistical test for archaic language morphology. Notably, four out of five (80%) are of 2c morphology (in its narrow definition, see Methods). This value is strikingly larger than the *a priori* probability for such 2c nouns in Semitic languages, say 12% in BH. It could be explained if most of the fire synonyms originate from an older proto-language that had an abundance of 2c lexemes. Normally, words are replaced approximately every 3,000 years, but some survive considerably longer [22,29]. These are typically the more frequently used ones. Since fire was so vital for existence it had to be manipulated daily. Consequently, prehistoric people must have used the word “fire” daily, and this explains its longevity. 

**Table 1:** PS synonyms for “fire” and “to burn.”.

**#**	**meaning**	**Akk.** [9]	**PS** (EA)	*DAE* ^1^ #	***ND/RPN*** #
1.1	fire (2c)	*i**š**ātu*	*ˀi**š**	1154	*ND* 86
1.2	"	***g**i**rr**u*	*g *i* ***r***(r)	1178	*ND* 688, *RPN* 443
1.3	"	*u**rr**u*	*ˀū**r**	1152	*ND* 73
1.4	"	***n**ū**r**u*	*nā**r**	1663	*ND* 1617
1.5	flame (3c)**^*2*^**	***n**a**bl**u*	*na**bl**	–	–
1.6	to burn (2c)	*a**gg**u*	*ˀ/**hg**	1155	*RPN* 596
1.7	"	*e**rr**u*	*ḥr	2648	–
1.8	"	***k**a**b**ā**b**u*	*kb	1192	*ND* 592?
1.9	"	***k**a**w**û* (?)	*kwy	1146	*ND* 1238
1.10	"	***q**â**d**u*	*qd	2465	–
1.11	"	***q**a**l**û*	*ql	1144	*ND* 1041
1.12	"	***q**a**m**û*	*qm	2193	*RPN* 466, *ND* 1068a
1.13	"	***š**a**b**ā**b**û*	*šb	1148	–
1.14	to burn (3c)	la*ˀ**b**u* **^*3*^**	*lhb	1799	–
1.15	"	***š**a**r**ā**p**u*	*śrp	–	–

Reconstructions are denoted by an asterisk, and root consonants (radicals) are in bold. *DAE* [18] entry numbers indicate suggested PAA origin, whereas a Nostratic origin is suggested by the cited entries from *ND* [20] and *RPN* [21]. See Table S1 of the Supporting Information for further detail.

^1^ There are PAA synonyms for “fire” that are unattested in PS. The full *DAE* list includes entries #1147, 1152, 1154, 1178, 1183-6, 1188, 1190, 1663, 2134 and 2599 there: all except #1185 are 2c.

***^2^*** I-*n* *na**bl** would be 2c in the broad definition of 2c roots (see Methods).

***^3^*** *lhb is PS only if Akk. la*ˀ**b**u* “skin disease” [9] is really a cognate. Otherwise there is only a single 3c/PS verb “to burn”.

 Corroboration of our conclusions can indeed be obtained from older proto-languages. Preceding PS on the linguistic genealogical tree is PAA (previously called Hamito-Semitic), from which the Afroasiatic (AA) language families (Semitic, Egyptian, Berber, Cushitic, Omotic, and Chadic) have evolved. Unlike Semitic and Old Egyptian, the other African languages have only recently been documented. Consequently, there is yet no consensus over the PAA lexicon, and whether it originated before or immediately after the transition to agriculture, i.e. between 9,000 [16] to 12,000 BP [17]. A Hamito-Semitic Etymological Dictionary (HSED) was published by Orel and Stolboba [12], and criticized by several authors [7,13,14]. It has since been updated online as the Database of Afroasiatic Etymology (DAE) [18], of which we make use in the tables below. An even older (and more controversial) conjectured macrofamily of protolanguages is Nostratic, for which two major dictionaries were compiled, abbreviated herein *ND* [20] and *RPN* [21]. It encompasses AA, Indo-European (IE), Kartvelian and other Euro-Asian language families, and is estimated to originate in the Levant some 15,000 years ago [19].

 Because our starting point is PS, we do not require that every item in these dictionaries be correct, only that they are sufficiently comprehensive to include the predecessors of most PS lexemes. The last two columns in Table 1 list entry numbers for PAA and Nostratic compilations, when exist. Of the five “fire” synonyms, all four 2c terms appear on both PAA and Nostratic levels, whereas the 3c term (*na**bl**) does not. Indeed, “fire” was found to be one of the "ultraconserved words" in the Nostratic macrofamily [22]. Thus all the pre-agricultural names for “fire” that survived in PS are ancient 2c terms.

#### (b): Burning statistics

A sample space of 5 items might be too small for statistical inference. Hence we add the 10 synonyms for the verb “set afire, burn” in Table 1. Of these, eight are 2c and only two are 3c. Again, all of the 2c terms are classified as PAA. The behavior in this semantic field confirms that most nouns and verbs connected with fire are 2c, contrasting with the low abundance of 2c lexemes in Semitic languages. 

#### (c) “Water”

like fire, is one of the "bare essentials" required to sustain life. Thus water vocabulary should also be immune to replacement. The PS noun for “water” is 2c *m *ā* ***y***, whereas drinking (water) is depicted by the two PS/2c verbal roots *št *y* and *šq *y* [8]. All three are also PAA (*DAE* entries 999, 1878 and 1209), hence of pre-agricultural origin.

#### (d): Hunting

Table 2 summarizes PS hunting terminology. The hunter had little possessions which he carried along: **bow** (#2.3) with which **to shoot** (#2.4) an **arrow** (#2.1), a **small bag** (#2.2) for collected items, perhaps a water bottle. All these PS terms are 2c and all are attested in PAA. The prehistory of the bow is difficult to determine because most components (except arrowheads) are perishable. Ballistic arrowhead analysis concluded [36] that lithic projectiles emerged with the onset of the Upper Paleolithic (ca. 45,000 BP). Nevertheless, the transition from atlatl to bow and arrow in W. Asia is believed to have occurred in Natufian times (15,000–11,700 BP), when both weapons may have been in use [37,38]. The fact that PS *qa**š**-t, “bow”, is agreeably PAA, and there is no obvious linguistic trace for “atlatl”, suggests that Natufians have utilized predominantly bows and arrows and/or that the term for atlatl has undergone a semantic shift to indicate the bow.

**Table 2:** Hunting terms in the PS lexicon (all are 2c).

**#**	**Meaning**	**Akk.** [9]	**PS** (EA)	**PAA** (ref. #)
2.1	Arrow	*ūṣu, uṣṣu*	*ḥiẓ***ẓ***	*T* 11
2.2	small bag	***k**ī**s**u*	*kī**s**	*T* 13
2.3	Bow	***q**a**š**tu*	*qa**š**-t **^*1*^**	*HSED* 1560, *DAE* 524
2.4	throw, shoot	***r**a**m**û*	*rmy	*DAE* 1499
2.5	hunt, prowl	*ṣâ**d**u*	*ṣd	*DAE* 1230
2. 6	provisions	*ṣi**d**ī**t**u*	*ṣī**d**	–

References to PAA origins include entry numbers from the treatises denoted herein *HSED* [12], *DAE* [18], and *T* [15].

See Table S2 of the Supporting Information.

^1^ In Semitic, “bow” ends with a feminine suffix, -*t*, that is missing in AA.

In addition to the overwhelming 2c vs. 3c statistics, we note an interesting polysemy (multiple meanings) of the PS root *ṣd (#2.5). While in BH it means “to hunt”, in Akk. *ṣâ**d**u* means “to prowl, turn about” [9]. Prowling characterizes a hunter-gatherer in search of food rather than a farmer. Farmers that occasionally went hunting would return to their permanent abode in a village [39]. Hunter-gatherer tribes in the Levant would spend the winter in the coastal plains, follow the deer in the spring to the mountains, and then *turn around*, completing an elliptic annual trajectory.

The pre-agricultural origin of this verb is supported by a possible pre-agricultural connection between hunting (#2.5) and **provisions** (#2.6). In PS, these must have been considered as homonyms (see EA), because for the farmer there was no connection between “hunting” and “provisions” (the latter coming mainly from his domesticated fauna and flora). For his hunter-gatherer predecessor, however, these must have been strongly associated, because provisions carried on hunting journeys might have included dried/smoked meat of hunted animals. Such a connection between hunting, prowling and provisions is thus indicative of a nomadic hunter-gatherer society. 

### II: The farmer's lexicon

 Evidently, more farming than hunting terms survived in PS, and nearly all have 3c morphology. Table 3 lists 27 agricultural terms that have been archeologically dated. Verbs like “collect”, “grind” and “bake”, characterizing both agricultural and pre-agricultural communities, and animal or plant names that could have originated either before or after domestication, are not listed. The discussion below provides archeological evidence that the entries in Table 3 originate within the Neolithic or Chalcolithic societies (ca. 11,000–6,000 BP).

**Table 3:** Agricultural terms in PS are of 3c morphology.

**#**	**Meaning**	**Akk.** [9]	**PS** (EA)	***DAE*** #
3.1	Farmer	*i**kk**a**r**u*	*ˀi**kk**a**r**	–
3.2	Storehouse	*i**s**i**tt**u*	*ˀa**s**a**m**	–
3.3	grape, fruit	*i**nb**u*	*ˁi**nb**	–
3.4	well, pit**^*1*^**	***b**ū**r**u*	*bi***ˀr***	916, 2536
3.5	ripe, cook	***b**a**š**ā**l**u*	*bšl	–
3.6	Terebinth	*buṭ**n**u*	*buṭ***m***/***n***	–
3.7	Millet	*duḫ**n**u*	*du**ḫn**	–
3.8	Livestock	*ṣā**n**u*	*ḍa**ˀn**	–
3.9	storage/threshing place	***g**a**r**ā**n**u*	*gu**rn**	–
3.10	arable land	*u**g**ā**r**u*	*hu**g**ā**r**	2327
3.11	Field	*e**ql**u*	*ḥa**ql**	–
3.12	to plow	*e**r**ē**š**u*	*ḥr***ṯ***	–
3.13	fermenting wine	*ḫa**mm**u**r**tu*	*ḫa**mr**	–
3.14	Butter	*ḫi**m**ātu*	*ḫimˀat	–
3.15	Village	***k**a**pr**u*	*ka**pr**	–
3.16	vine(yard) **^*2*^**	***k**a**r**ā**n**u*	*ka**rm**	1050
3.17	mud brick	***l**i**b**i**tt**u*	*la**b**i**n**at	–
3.18	stockbreeder	***n**ā**q**i**d**u*	*nā**q**i**d**	–
3.19	Canal	***p**a**lg**u*	*pa**lg**	–
3.20	Flour	***q**ē**m**u*	*qam**ḥ**	–
3.21	Trough	*rāṭu*	*raha***ṭ***	–
3.22	to draw water	***s**â**b**u*	*š***ˀb***	984
3.23a	Beer	***š**i**k**a**r**u*	*ši**k**a**r**	–
3.23b	be drunk	***š**a**k**â**r**u*	*škr	–
3.24	boil, cook	***s**a**l**ā**q**u*	*šlq	–
3.25	to plant	***s**a**t**ā**l**u*	*štl	–
3.26	straw, chaff	***t**i**bn**u*	*ti**bn**	–
3.27	to sow	***z**a**rû***	*zr**ˁ**	2338

Only 5 are possibly PAA, and even some of these assignments are questionable.

See Table S3 of the Supporting Information.

***^1^*** #3.4 is not PAA if the Chadic and Cushitic cognates are Arabic loans (*DAE* #916).

***^2^*** The PAA status of #3.16 “vineyard” relies on an Egyptian cognate which is likely a W. Semitic loan (see *EA*)*.*

 As of the Pre-Pottery Neolithic B (PPNB), ca. 10,500 BP, the **farmer** (#3.1) lived in a large **village** (#3.15), constructed of square houses [3], often made of **straw** (#3.26) reinforced [40] sun-baked mud **bricks** (#3.17). Indeed, straw became readily available after the Pre-Pottery Neolithic A (PPNA) wheat domestication [1], and hence its identification as an agricultural commodity. 

 The farmer would work in an agricultural **field** (#3.10, 3.11), which he would **plow** (#3.12), **sow** (#3.27) or **plant** (#3.25). Furrow tracts from W. Europe date to 5,500 BP [41] and must have appeared earlier in W. Asia. Cattle were domesticated in the upper Euphrates valley by 10,000 BP, spreading to Central Anatolia, Mesopotamia and the S. Levant around 8,500 BP [42]. This may mark the onset of ox-traction and hence the use of the scratch-plow (ard) for plowing. The ard might have also been instrumental in installing the first irrigation systems. An early irrigation **canal** (#3.19), over 7 kyr old, was discovered in Choga Mami, 110 km E. of Baghdad: "It is conceivable, indeed probable, that plough cultivation accompanies irrigation agriculture in the earlier Samarra period" [48]. 

 Tilled fields can be sown only if grain from the previous year is stored under adequate conditions. PPNA **granaries** (#3.2 and 3.9), about 11,300 years old, were unearthed in the Dead-Sea region near Dhraʽ, Jordan [43]. These round structures, with suspended floors for air circulation and protection from rodents, were located between residential structures that contain plant-processing installations.

 The first attested **wells** (#3.4) were dug by Neolithic farmers on the coast of Cyprus ca. 9,200 BP [44]. The oldest well found in Israel (8700–8400 BP) is in the undersea site of Atlit-Yam [45,46]. A Pottery Neolithic (PN) well, dated to ca. 8,300 BP, was found at Sha‘ar Hagolan in the Jordan Valley [47]. Thus wells were yet another important innovation of the Neolithic. The II-*ˀ* morphology of *bi***ˀr*** “well” (#3.4) is also attested in the PS/3c verbal root for **drawing water** (š***ˀb***, #3.22), possibly because they have originated in the same period.

 The earliest mineral-tempered ceramics from Tell Sabi Abyad (N. Syria) was likely introduced for **cooking** (#3.5 and 3.24), leading to a "culinary revolution" nearly 9,000 years ago [49], when (PS/2c) “baking” [8] and “roasting” (#1.11) were supplemented by cooking. Only later was pottery utilized for storing liquids. 

 Herding began after goat and sheep domestication, either in the Neolithic or as late as the Chalcolithic [50]. **Livestock** (#3.8) was often composed of mixed sheep and goat herds that optimize vegetation exploitation. This contrasts with their non-overlapping habitats in Nature [2], suggesting that *ḍa***ˀn*** “livestock” (#3.8) is a post-agricultural innovation. The herd was lead by a **stockbreeder** (#3.18) to a **drinking trough** (#3.21). Due to lactose intolerance, milk utilization has begun rather late, at the end of the Chalcolithic or the Early Bronze [41]. However, recent fatty acid analysis of pottery sherds suggests that processed milk was used as early as 8,500 BP [51]. In agreement with this, there is no PS name for milk but there is one for **butter** (#3.14), a low lactose milk product.

**Fermenting wine** (#3.13) was made from **grapes** (#3.3) already in the Neolithic: jars from Georgia (in the Caucasus), dating to ca. 8,000 BP, were shown to contain resinated wine deposits, as have 7,300 BP sherds from the Zagros Mountains in Iran ([52], Chap. 4). The popular resin was from the **terebinth** tree (#3.6), *Pistacia atlantica* [52]. The earliest known winery (6100 BP) was recently found in an Armenian cave site [53]. The prominence of viticulture in the Fertile Crescent is echoed in toponyms derived from *ka**rm**, *ka**r**ā**n** “vineyard” (#3.16): Mt. *Karmel* in N. Israel and *Karānā* in Upper Mesopotamia (perhaps Tell ar-Rimāh, 60 km W. of Nineveh). Although a dry wasteland today, the high concentration of archeological mounds suggests it has once been fertile land ([52] p. 173). 

**Beer** (#3.23a) was the most popular intoxicating (#3.23b) drink in Mesopotamia. Until recently, the earliest evidence for beer (from ca. 5,500 BP) was found in the Sumerian trading post of Godin Tepe in Iran [54]. But recent evidence from Göbekli Tepe (S.E. Turkey) suggests that beer was brewed already in the PPNB [55]. 

**Millet** (#3.7) was domesticated in N.E. China about 10,000 years ago [56]. It made its way to the Black-Sea region around 7,000 BP [57], just in time to be included in the PS lexicon. Because it came from outside W. Asia, its PS name depicts the domesticated plant and not its wild progenitor.

**Exceptions** to the regularity demonstrated in Table 3 namely, PS agricultural terms with 2c morphology, are hard to find. We have found two such examples (as compared with 27 entries in Table 3), and even these are not clearly exceptions. (i) It is suggested that PS/PAA/2c *ma**rr** “a hoe”, derived from the 2c root *mrr “to hoe”, originates within a PAA farming lexicon [16]. The noun is either Nostratic, *ND* #1482 [20], or a "wandering-word" borrowed into many languages from Sumerian [58]. If the verb *mrr has itself been borrowed by Sumerian from PAA [16], then its original meaning was “to dig” [59], an activity practiced by hunter-gatherers much before the agricultural era. (ii) The PS verb for **herding**, *r*ˁy*, is 2c although herding postdates ungulate domestication that occurred after the transition to agriculture. However, in some Chadic dialects it means “to chase, follow”, *DAE* #663 [18]. This may go back to gazelle chases, involving gathering herds by "effective utilization of drives and surrounds" [60], including the utilization of huge traps known as "desert kites" [61]. Thus if gathering domesticated herds is the behavioral continuation of gathering herds of gazelles, the continued use of the same verb for depicting it could be understandable.

### III: Word Frequency Analysis

**Figure 1:**
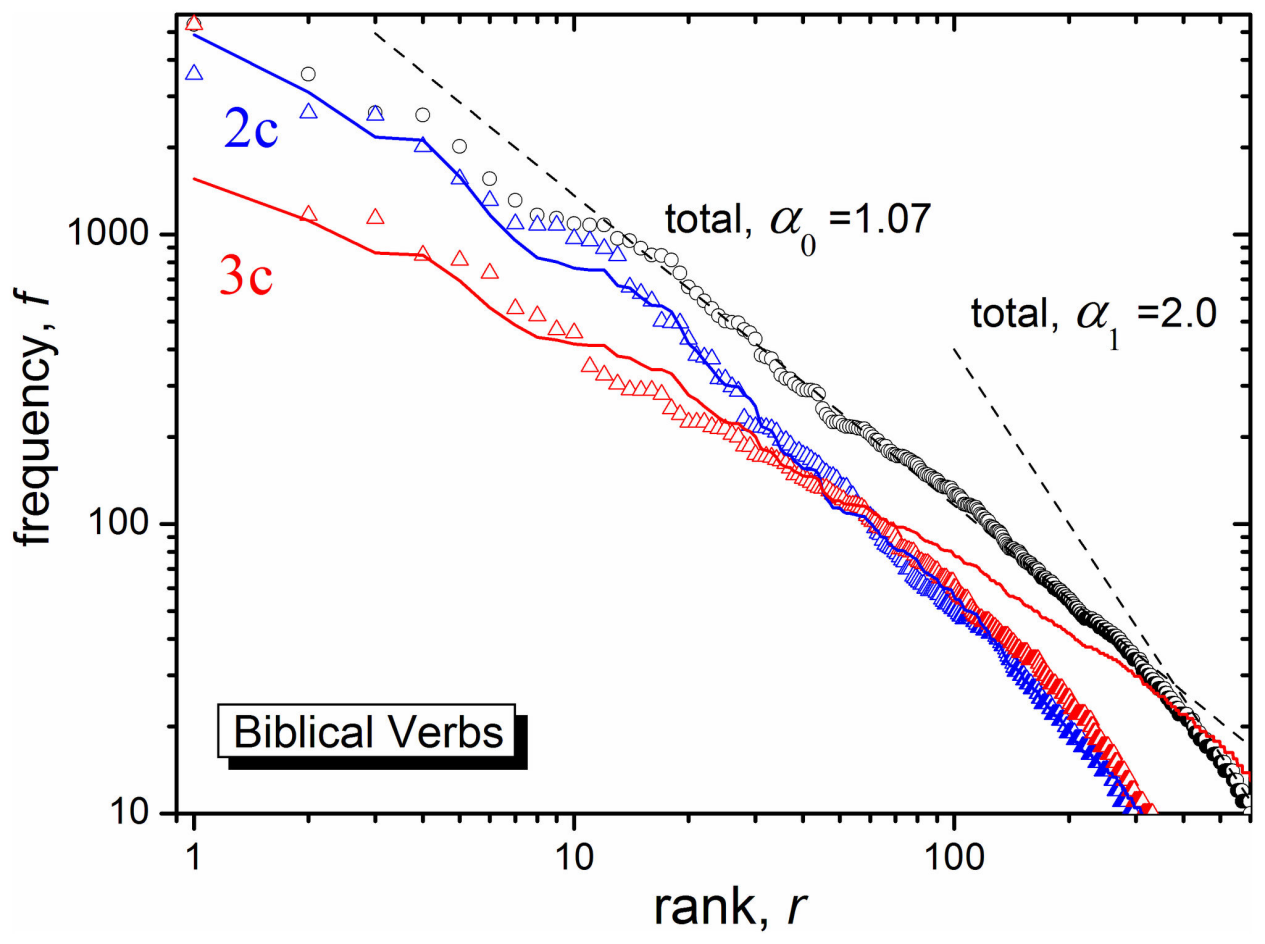
Frequency-rank plot for Hebrew verbal roots appearing more the 10 times in the Bible (black circles) **[31]**. Black dashed lines: fits of the total frequency,*f*_0_(r) , to Eq. (1) with *A*_0_=16,000 and *α*_0_=1.07or *A*_1_=1.3×10^6^ and*α*_1_=2. Blue triangles represent 2c/BH verbal roots in their broadest definition (see Methods). They were extracted from Lester's list [31] and collected in Table S5 of the Supporting Information. The non-2c verbs there were defined as 3c, and their frequencies are depicted by the red triangles. Blue line is a fit to Eq. (2) with *t* = 7.8 kyr and*A*_2c_=3. The rate function *k*(*f*_0_) from Eq. (3b) has *B* = 0.55 kyr^-1^ and β = 0.13, as deduced from Figure 3a of Ref [29].. Red line is a fit to Eq. (4) with the same parameters, except for*A*_3c_=0.09.

 The study thus far focused on statistics of culturally specific terms (hunting vs. farming) that could be correlated with archeology. These are mostly low frequency lexemes, hence not belonging to the "kernel lexicon". We now analyze the kernel lexicon of an Ancient Semitic text, the Hebrew Bible, bisecting it into its 2c vs. 3c components. We consider verbs, because their 2c vs. 3c origin can be determined rather mechanically (see Methods), allowing processing a large number of verbs. Yet they constitute the only part of speech whose Zipf plot is similar to that of the whole corpus [25]. The black circles in Figure 1 depict the frequency-rank dependence, *f(r)*, for the BH (non-Aramaic) verbal roots with f ≥ 10 [31]. It indeed appears that there are two regimes here [26], with *α*_0_=1.07and *α*_1_≈2 (dashed lines). The switchover occurs around f = 20, so that the kernel of BH is characterized by f ≥ 20. 

Table 4 lists the 20 most frequent BH verbs. These are indeed very generic, not related to any specific culture or occupation, and likely used with high frequency in any natural language. Of these, 13 are 2c, far exceeding the fraction of 2c verbs in the Bible. This agrees with the observation that the most frequently used words in English tend to be short [24] (and also of Old English origin). According to Zipf's "principle of least effort" long words got shortened for ease of use. We have no evidence that 2c/PS verbs were shortened from 3c verbs, and thus suggest another mechanism leading to the prevalence of 2c verbs in Table 4.

**Table 4:** The 20 most frequently used verbal roots in BH with their Biblical frequencies [31].

**rank**	**Meaning**	**BH** (freq.)	**Akk.** [9]	***DAE*** #	**2c/3c**
1	say, see	***ˀmr*** (5317)	*a**m**ā**r**u*	–	3c
2	become	*hyh* (3576)	*e**w**û*	2056	2c
3	do	*ˁ**ś**y* (2632)	–	532	2c
4	come	bw***ˀ*** (2579)	*bâˀu*	599	2c
5	give	*n**tn*** (2014)	*na**d**ā**n**u*	1237	2c
6	go	*h**lk*** (1554)	*a**l**ā**k**u*	615	2c
7	see	r*ˀy* (1310)	–	887	2c
8	hear**^*1*^**	šm***ˁ*** (1165)	***š**e**mû***	242	3c
9	speak	***dbr*** (1135)	–	874	3c
10	sit	*y**šb*** (1087)	*wa**š**ā**b**u*	3072?	2c
11	go out	yṣ***ˀ*** (1075)	*wa**ṣû***	–	2c
12	return	***š**w**b*** (1075)	–	–	2c
13	take	lq***ḥ*** (966)	***l**e**qû***	–	3c
14	know	yd***ˁ*** (952)	*e**dû***	–	2c
15	ascend	*ˁ**l**y* (894)	*e**lû***	–	2c
16	stretch out	šl***ḥ*** (847)	***š**a**lû***	–	3c
17	die	***m**w**t*** (845)	***m**â**t**u*	2466	2c
18	eat	***ˀkl*** (814)	*a**k**ā**l**u*	1197?	3c
19	call**^*2*^**	qr***ˀ*** (736)	***q**e**rû***	879	3c
20	lift	nś***ˀ*** (658)	*na**šû***	1627	2c

Those with Akk. cognates are PS, whereas *DAE* entry numbers [18] indicate possible PAA origin. See Table S4 of the Supporting Information.

^1^The listed AA cognates mean “ear” and they are 2c (*DAE* #242).

^2^The AA reconstruction means “shout” and it is 2c (*DAE* #879).

 As recently shown [29], frequently used words (actually, *meanings*) are replaced (by other words of the same meaning) less often than the less frequent ones. Thus if the 2c stratum indeed predated the 3c one, the frequently used 2c lexemes may have simply survived replacement during the subsequent 3c era. This is supported by their frequency-rank dependence in Figure 1. As opposed to the total BH verbs with α ≈ 1, the 2c/BH verbs (collected in Table S5) exhibit an observably larger Zipf exponent (*α*_2c_=1.28), whereas the high frequency 3c verbs have a smaller *α*_3c_=0.82(linear fits not shown). This might be explainable by the 2c > 3c transition: while the 2c language was alive, 2c words of a given meaning were depleted at the same rate as alternate 2c lexemes were generated, and the language maintained its steady-state with the usual exponent α ≈ 1. After the 2c era has ended, 2c roots were no longer created only eliminated. Because less frequently used words decay faster, α_2c_ increased with time. 

 One may turn this into a quantitative method for dating the 2c > 3c transition. Suppose that once there were only 2c words, and at some time (*t* = 0) they started to be replaced with new 3c words. Assume that (up to a constant) the frequency of use of a certain verbal *meaning* (at least in the kernel lexicon),*f*_0_(r) , is an inherent property of human language and hence not strongly time-dependent. We thus equate it with the frequency of the total verb distribution (black circles in Figure 1). Therefore, at *t* = 0 the 2c frequency-rank relation was*f*_2c_(*r*,0)=*A*_2c_ *f*_0_(r), where *A*_2c_ is some constant. We expect *A*_2c_>1 if the 2c corpus was once used more frequently than today (or: with a smaller vocabulary each word is used more frequently).

 Subsequently, the frequency of 2c verb utilization decayed exponentially with time:

$$f_{\text{2c}}(r,t)=A_{\text{2c}}f_{0}(r)\exp[-k(f_{0}(r))t]$$ (2)

The rate coefficient,*k*(*f*_0_) , is a unique function of the (time-independent) verb *meaning* frequency,*f*_0_(r). Eventually, after some time *t* that we opt to determine,*f*_2c_(*r*,*t*) reached the values observed in the Biblical lexicon (blue triangles in Figure 1). 

 A similar equation was suggested by Leiberman et al. [28], see their supporting Eq. (3). It can be interpreted in two ways. Firstly, like in radioactive decay: the decay of any particle is instantaneous, and one counts the number of particles surviving by time *t*. This is useful when texts from different epochs are available, as in [28], but not for the analysis of a single text. However, words need not disappear instantaneously from the lexicon. Their use may gradually decrease over time until they eventually become obsolete, and this allows applying the above equation even when text(s) from just a single period are available.

 To proceed, a functional form for *k*(*f*) is required. We adopt Pagel et al. [29] power-law rate coefficient for lexical replacement. It depends on the part of speech, but otherwise is rather universal for the IE family, and possibly for all languages [22]:

$$k(f)=B/f^{\beta }$$ (3a)

From the correlation line for English verbs in their Figure 3a, one estimates *B* = 0.55 kyr^-1^ and β = 0.13. We *do not* vary these parameters in fitting our data. However, in Ref. [22] the frequencies are per million words of text, whereas in the Bible there are about 305,500 Hebrew words (a ratio of 3.27), hence what we insert into Eq. (2) is: 

$$k(f_{0})=B/{\left(3.27f_{0}\right)}^{\beta }$$ (3b)

Adjusting *t* and *A*_2c_ to fit the 2c data (blue triangles), we obtain the blue line agreeing with the data over the whole frequency range, even where it deviates from Zipf's law, Eq. (1). This gives *t* = 7.8 kyr. Adding the presumed age of BH, ca. 3 kyr, gives 10.8 kyr for the 2c > 3c transition, agreeing nicely with the onset of agriculture. 

 An analogous model may describe the 3c verbs, which experience exponential *growth* rather than decay:

$$f_{\text{3c}}(r,t)=A_{\text{3c}}f_{0}(r)\exp[k(f_{0}(r))t]$$ (4)

Of course, such growth cannot go on indefinitely, but we assume the time-depth is not large enough to observe saturation. With exactly the *same* parameters as above (excepting *A*_3c_) we obtain the red line in Figure 1, which fits the 3c data at high frequencies. Thus 10.8 kyr BP marks both the end of the 2c era and the onset of 3c morphology.

**Figure 2:**
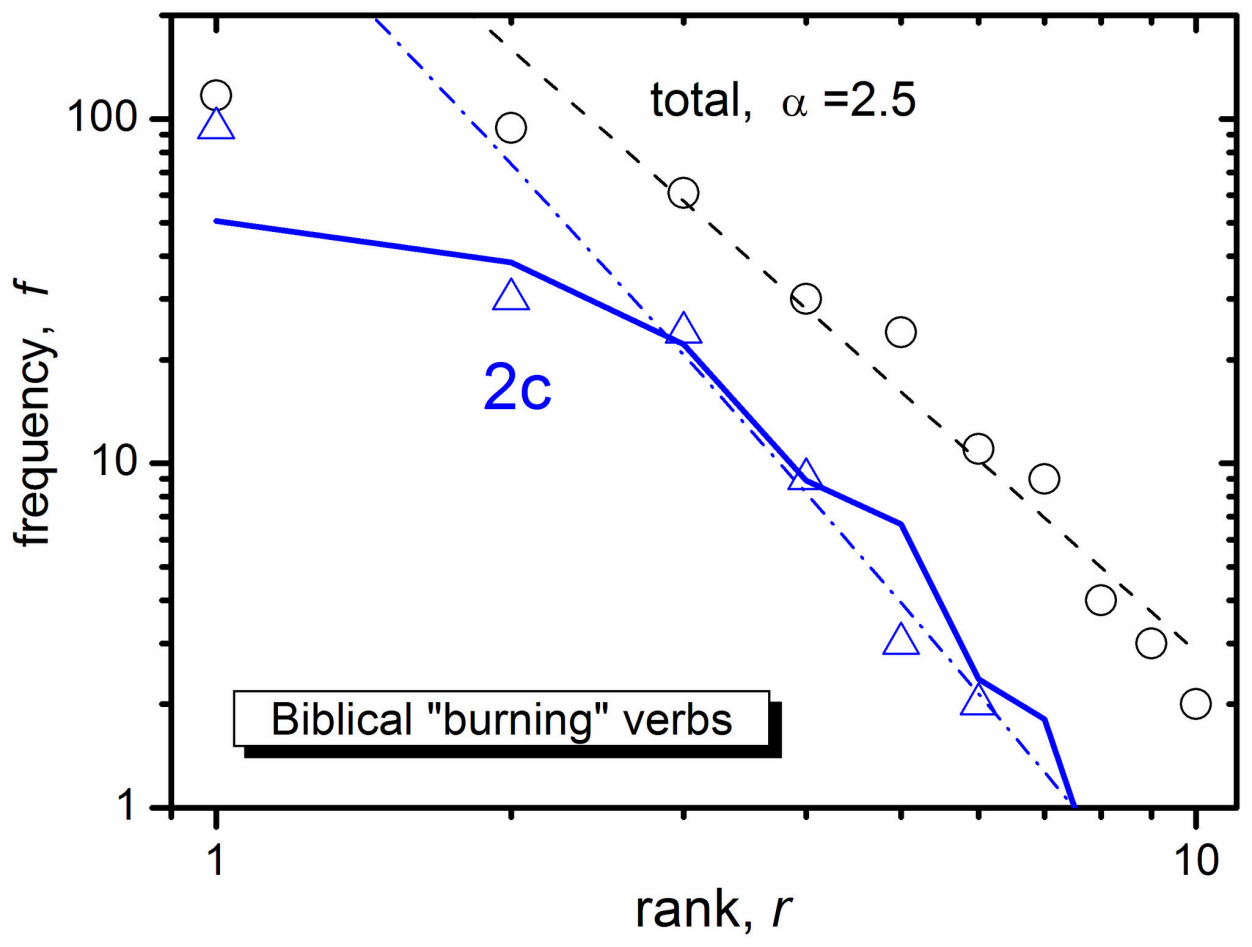
Frequency-rank plot for BH verbal roots that are near synonyms of “to burn” (circles), and their 2c subgroup (triangles). Dashed line represents Eq. (1) with *A* = 900 and α = 2.5. The application of Eqs. (2) and (3b) to it gives the dash-dotted line, whereas their application to the data itself (circles) gives the full line. Parameters are identical to those in Figure 1. The frequencies of the 10 roots were taken from a Biblical Concordance, and are as follows: ***śrp*** 117 (3c), *ḥry* 94 (2c), b***ˁr*** 61 (3c), y***ṣt*** 30 (2c), *kby* 24 (2c), lh***ṭ*** 11 (3c), *yqd* 9 (2c), ***dlq*** 4 (3c), *qly* 3 (2c), *kwy* 2 (2c).

 As a check for the robustness of this analysis, we return to the "burning verbs" discussed in Subsec. I(b) above. We find 10 such verbs in BH (some of these are PS, and thus appear in Table 1). Their frequency-rank relation is shown in Figure 2 (circles). The deviation from Zipf's law, dashed line, is even larger and its exponent α = 2.5. Of these verbs, 6 are 2c (triangles). Although a rather small collection, we can repeat our analysis. Remarkably, when Eqs. (2) and (3b) are applied to the data, with exactly the same parameters as in Figure 1, we obtain either the dashed-dotted line (when the dashed line is used as*f*_0_), or the full line (when the circles are used as*f*_0_). Thus the "burning verbs" behave like the entire BH verb population, both yielding the same date for the 2c > 3c transition.

## Conclusions

 In this work PS hunting vs. farming terms were collected based on the significance accorded to them in the archeological literature. Material innovations are paralleled by linguistic innovations namely: new names for new material objects and new verbs depicting their utilization. This allows to tentatively *date* these words independently from the comparative linguistic evidence.

 From the hunter-gatherer period mostly the frequently used words have survived change. “Fire” and “water” must have been such words, because they were essential for daily survival. The associated verbs are “to burn” and “to drink”, respectively. We have collected all the PS synonyms of these four lexemes finding remarkable correlations: (a) Most of them are also PAA and/or Nostratic (corroborating their pre-agricultural origins) and (b) of 2c morphology. A similar trend is observed for PS hunting terms, which are all 2c.

 The farming terms collected in Table 3 are those attributed by archeological studies to innovations of the Neolithic and Chalcolithic periods in W. Asia. These *all* have 3c morphology, and only rarely possess PAA cognates. We were able to find very few exceptions to this rule, and these represent secondary use of existing 2c roots. Hence PS hunting vs. farming lexicons have, on average, different time-depths and morphologies. Likely, then, a 2c-enriched hunter-gatherer language has evolved into a 3c-dominated farmer language with the transition to agriculture in W. Asia. 

 This suggestion is corroborated by a frequency analysis of BH verbs. While the total verbal corpus exhibits a Zipf plot with the expected exponent of about unity, its 2c subset has an observably larger exponent. This can be understood if the creation of new 2c roots ceased sometime in prehistory, and thereafter the use of the low frequency 2c verbs decreased faster than those of higher frequencies. This was turned into a novel quantitative method for dating the 2c > 3c transition. The date obtained, nearly 11 kyr BP, indeed marks the transition from hunting to farming. Thus two independent methods, applied to different parts of the Semitic lexicon ("specific" vs. "kernel"), lead to the same conclusion namely, that a major change in human lifestyle (the transition to agriculture) correlates, in W. Asia, with a major linguistic change.

## Supporting Information

Text S1 **Explains how the Proto-Semitic word reconstruction was achieved.**
(PDF)

Table S1 **Etymological Appendix for Table 1.**
(PDF)

Table S2 **Etymological Appendix for Table 2.**
(PDF)

Table S3 **Etymological Appendix for Table 3.**
(PDF)

Table S4 **Etymological Appendix for Table 4.**
(PDF)

Text S2 **List of references for the Etymological Appendix.**
(PDF)

Text S3 **The complete Etymological Appendix composed of the above six supporting files.**
(PDF)

Table S5 **Ranking of “weak” (originally 2c) verbal roots in the Hebrew Bible by frequency.** The 2c roots were extracted from the list in Ref. 31 according to the "broad definition" in the Methods section.
(PDF)

Table S6 **Transliteration: Proto-Semitic consonant phonemes with their Hebrew and Arabic equivalents.**
(PDF)
