# Supplementary material for: Statistics of Language Morphology Change: From Biconsonantal Hunters to Triconsonantal Farmers
Source: PLoS One. 2013 Dec 19;8(12):e83780. doi: 10.1371/journal.pone.0083780 (PMC3868553; doi:10.1371/journal.pone.0083780)
Supplement: Text S1 — Explains how the Proto-Semitic word reconstruction was achieved. (PDF) [file pone.0083780.s001.pdf]

Supporting Information for

Statistics of Language Morphology Change: From Biconsonantal Hunters to Triconsonantal Farmers

(by Noam Agmon)

Etymological Appendix

Yigal Bloch, Department of Jewish History, The Hebrew University, Jerusalem 91904, Israel.

The leftmost column of the following table includes the number of the relevant entry in the tables of the main text.

The second column from the left presents proto-words whose reconstruction is based on actual words attested in different Semitic languages. For the purposes of this reconstruction, a proto-word is a lexical unit expressing a definite semantic notion and possessing a more-or-less stable morphology – i.e., the same set of radicals (with possible metathesis of the radicals, or interchanges of homorganic consonants), and a small set of patterns (defined by the placement of vowels, prefixes and suffixes) in which those radicals are materialized. In some instances, the variety of morphologically and semantically related forms in the individual languages does not permit the reconstruction of a single form for a given proto-word. Nevertheless, the very existence of morphological and semantic similarity between the attested forms strongly suggests that they are reflexes of earlier forms belonging to a linguistic stratum that existed prior to the languages to which the attested forms belong.

Each reconstructed proto-word is classified as Proto-Semitic (PS) if its reflexes can be recognized in additional Afro-Asiatic languages beyond the Semitic family, or are attested in East Semitic (Akkadian) and at least one other Semitic language (while not an Akkadian or non-Semitic loanword in the latter). If no reflexes of a given proto-word are attested either in non-Semitic Afro-Asiatic languages or in Akkadian, that proto-word is classified as Proto-West Semitic (PWS). All proto-words of this kind quoted in the table below are attested, on the one hand, in Ethiopic or Modern South Arabian (the South Semitic languages), and on the other hand, in Hebrew, Aramaic, Ugaritic or Arabic (the Central Semitic languages). A proto-word whose reflexes are attested only in some of the latter four languages (including necessarily Arabic) is classified as Proto-Central Semitic (PCS).

The classification of languages adopted here is based on Huehnergard 2005.

Abbreviations used for Aramaic dialects:

BArm. = Biblical Aramaic

CPArm. = Christian Palestinian Aramaic

JArmTg = Jewish Aramaic of Targum Onkelos and Targum Jonathan (both composed in Palestine and edited in Babylonia in the early first millennium C.E.)

JBArm. = Jewish Babylonian Aramaic

JPArm. = Jewish Palestinian Aramaic

Mnd. = Mandaic
OArm. = Old Aramaic (9th-6th centuries B.C.E.)
OffArm = Official Aramaic (6th-4th centuries B.C.E.)
Palm. = Palmyrene (early centuries C.E.)
Sam. = Samaritan Aramaic
Syr. = Syriac

Dictionaries and lexicographical works used for individual languages and language groups:
Akkadian: *AHw*; *CAD*; *CDA*
Arabic: *AEL*; BK; Hava 1899
Aramaic: CAL; Jastrow 1996 (1903); Sokoloff 2002a; Sokoloff 2002b; Sokoloff 2003; Sokoloff 2009; Tal 2000
Geˁez: *CDG*
Hebrew: *BDB*; *HALOT*
Modern South Arabian: *JL*; *LSoq*; *ML*
Tigrinya: Kane 2000
Ugaritic: *DULAT*

For the identification of loanwords (lw.), beside the dictionaries of the specific languages, the following studies were used: Fraenkel 1962 (1886); Kaufman 1974; Leslau 1990; Lieberman 1977; Mankowski 2000. Identification of loanwords in the table without further discussion is based on the dictionaries and the abovementioned studies.

Proposals for the identification of additional words in specific languages as loanwords, or objections against existing identifications of loanwords, are discussed in the footnotes to the table.
