# Supplementary material for: Statistics of Language Morphology Change: From Biconsonantal Hunters to Triconsonantal Farmers
Source: PLoS One. 2013 Dec 19;8(12):e83780. doi: 10.1371/journal.pone.0083780 (PMC3868553; doi:10.1371/journal.pone.0083780)
Supplement: Table S1 — Etymological Appendix for Table 1. (PDF) [file pone.0083780.s002.pdf]

Table S1. Etymological Appendix for Table 1 (by Yigal Bloch)

No.	Proto-word	Hebrew	Aramaic	Ugaritic	Arabic	Modern South Arabian[1]	Ethiopic[2]	Akkadian[3]
1.1	**ˀiš, *ˀišāt* "fire" (PS, 2c)	*ˀēš* "fire"	*ˀš* (OArm.), *ˀšh* (OffArm.), *ˀeššāˀ*, *ˀeššātāˀ* (BArm., JArmTg., JPArm., JBArm., Syr.) "fire"	*išt* "fire"			*ˀəsāt* "fire"	*išātu* "fire"
1.2	**gir*(*r*) "fire, heat" (PS, 2c)[4]				*ǧāyirun* "bur-ning, intense heat inside the body"			*girru* "fire, fire-god"
1.3	**ˀūr* "fire"; **ˀār*, **ˀurr* "light" (PS, 2c)	*ˀūr* "fire"; *ˀōr* "light"	*ˀwr* "to shine" (JPArm.)	*ar*, *ir* "light"; *ur* "warmth, fire"	*ˀuwārun* "heat of fire"		*ˀarwa* "to flame, blaze" (Tigre)	*urru* "daytime"

[1] Recent studies in the attested Epigraphic South Arabian languages suggest that "none of these languages can be the ancestor of either the Modern South Arabian languages or the Ethiopian Semitic languages" (Huehnergard 2005: 161, and see the earlier studies cited there). Since Epigraphic South Arabian languages appear to belong to the Central Semitic branch of the Semitic language family (Huehnergard 2005: 160-161), and since the Central Semitic languages are already represented in the present table by Hebrew, Aramaic, Ugaritic and Arabic, this column is restricted to Modern South Arabian languages.

[2] Unless otherwise noted, the words in this column are from Geˁez.

[3] Final mimation in Akkadian nominal forms is not marked, unless a specific syllabic spelling with final mimation is quoted.

[4] Akkadian *girru*, reflecting formally the geminate root *grr*, and Arabic *ǧāyir*, reflecting formally the hollow (II-*y*) root *ǧyr* (BK: 361b), suggest that both these forms are extensions of the original 2c form **gir*(*r*).

1.4	**nūr* "light"; **nār* "fire" (PS, 2c)[5]	*nēr* "light, small clay lamp"; *nîr* "light, lamp"	*nūr*, *nūrāˀ* (BArm., JArmTg., JBArm., JPArm., Syr.) "fire"; *nəhōr*, *nəhōrāˀ* (BArm., JArmTg., JBArm., JPArm.), *nūhrāˀ* (Syr.) "light"[6]	*nr* "to shine, burn" (verb); *nr* "sheen, glean, lamp" (noun); *nrt* "lantern, lamp"	*nārun* "fire"; *nūrun* "light"		*nār, nūr* "light, fire" (Arabic lw.)	*nūru* "light, gleam"; *nawāru* "to be(come) bright, shine"
1.5	**nabl* "flame" (PS, 2c)			*nblu* "flame"	*nablun* "ar-row"[7]		*nabal* "flame"	*nablu* "flame, flash of fire, fire-arrow"
1.6	**ˀg*, **hg* "to burn,				*ˀaǧǧa* "to burn,	*ɛhgég* "to make a big	*hagägä* "to smoke	*agāgu* "to be(come)

[5] It has been argued that in PS, **nūr* meant exclusively "light," and the use of **nūr*/**nār* for "fire" in individual languages was a later semantic development (Kogan 2011: 194-195). Indeed, in Aramaic, the use of *nūr* for "fire," instead of the earlier **ˀēš*, is a relatively late phenomenon, whose emergence can be traced in historical times (Kogan 2005: 558). However, no similar indication of a relative lateness exists for Arabic *nārun* "fire", and the verb *nr* appears to be attested in Ugaritic with the meaning "to burn" beside "to shine" (*DULAT*: 641-642; see also Del Olmo Lete 2004: 297, n. 16, 299, n. 21). It is also possible that Akkadian *tinūru* "oven," and reflexes of the same word in West Semitic (Hebrew *tannûr*, Aramaic *tannūrāˀ*, Arabic *tannūr*) are genuine Semitic derivatives from the root *nwr*, which would support the inclusion of burning, beside lighting, in the original semantic field covered by this root (see Hoch 1994: 359, n. 14). However, it is equally possible that the West Semitic words for "oven" are all loans (direct or indirect) from Akkadian *tinūru*, whereas the latter is itself a loan from Sumerian DURUN "oven" or a so-called culture word – i.e., a word common to many languages from different linguistic families over a wide geographical area, for which no specific etymology in any known language can be established (thus Mankowski 2000: 150-151; and see also Civil 1973: 174, who hesitates between the possibilities of Semitic origin, implying a loan from Akkadian, and a culture word for Sumerian DURUN).

[6] In Aramaic, the hollow roots (II-*w/y*) are sometimes extended to a 3c pattern through the addition of *h* as the second radical; cf. Aramaic *rhṭ* "to run," Hebrew *rwṣ*, PS **rwẓ*. This phenomenon supports the classification of the hollow roots as originally 2c.

[7] Arabic *nablun* "arrow" may reflect either the actual use of flaming arrows in ancient warfare, or a metaphorical usage of "flame" to denote an intensive use of weapons in a battle (cf. the usage of *nablu* in Akkadian in contexts related to warfare – *CAD* N/1: 26a-b, s.v. *nablu* A, 1b).

	blaze (literally or figuratively)” (PS, 2c)[8]				blaze (said of fire)”; *hağğa* “to burn fiercely (said of fire)”	blaze, flash” (causative stem) (Jibbāli)	(said of fire), produce more smoke than flame, give off a strong, penetrating odor” (Tigrinya)	angry, flare up in anger”
1.7	**ḥr* “to be hot, burn” (PS? PWS? 2c)[9]	*ḥrh* (*ḥry*) “to be/ become hot, angry”; *ḥrr* “to burn”	*ḥry* “to be hot, glow, rake” (JBArm.); *ḥrḥr* “to set on fire” (JArmTg.)	*ḥrr* “to dry up, shrivel, burn up, catch fire”	*ḥarra* “to be hot, burn up”; *ḥarrun* “heat”	*ḥəráwrət* (Mehri) “heat, heat-spot, burnt food at the bottom of a pan”	*ḥarra*, *ḥarara* “to burn (intrans.), to be ablaze, hot, grilled, dried up”	*erēru* “to be parched (?)”; *erru* “parched (?)”[10]
1.8	**kb* “to burn, char, roast” (PS, 2c)[11]		*kbb* “to char” (JBArm., Akkadian lw.)		*kabābun* “roasted or broiled meat”	*kbb*, *qeb*(*b*) “to roast” (Soqoṭri); *qbb*		*kabābu* “to burn, scorch, char wood”

[8] The attested reflexes allow the reconstruction of both *ˀ*g* and **hg* (*ˀ*gg* and **hgg* in the 3c notation) for PS, and indicate that the original verb was, in all likelihood, intransitive. The variation ˀ/*h* is actually attested in the Arabic reflexes of the PS verb (ˀ*ağğa* vs. *hağğa*) and is explained by the fact that the consonants ˀ and *h* are homorganic: voiced vs. voiceless laryngeal consonants, respectively (see Lipiński 2001: §19.2).

[9] Whether this root can be reconstructed back to PS or only to PWS depends on the interpretation of the Akkadian evidence (see the following note).

[10] The meaning of the Akkadian term is not clear. *AHw*: 238b, 244a, translates the verb *erēru* “to be parched?” (“‘dürr sein’ ?”) and the adjective *erru* “approximately ‘to be parched’” (“etwa ‘dürr’”); note the indications of uncertainty of the translation, specified in the original. In contrast, *CAD* E: 280a, 307b, translates *erēru* “to become moldy” and *erru* “moldy” (see further the discussion of *erēru* in *CAD* A/2: 238a-b, s.v. *arāru* C). In Sumerian-Akkadian lexical lists, the word ^{GIŠ}*erru* (where ^{GIŠ} is the determinative for the semantic category of wood) appears as equivalent to Sumerian ^{GIŠ}BÍL(gibil) and ^{GIŠ}GÍBIL “firewood, tinder” (*AHw*: 244a; *CAD* E: 307b). Wood used for fire would be more likely parched than moldy. Hence, the translation specified in *AHw* appears preferable, and is hesitantly adopted in the present table, which makes it possible to reconstruct the 2c base **ḥr* “to be hot, burn” back to PS.

[11] Hebrew and Aramaic *kbh* (*kby*) "to be quenched," and Arabic *kabā* (*kbw*) "to smoulder" (*HALOT*: 457a), are formally derived from the 3c root *kbw*. Both the roots *kbb* and *kbw* would be derived from original 2c **kb*, and a strong association between

						“to roast potatoes” (Mehri)		
1.9	**kwy* “to burn, cauterize” (PS, 2c)	*kwh* (*kwy*) “to burn, scorch”	*kwy* “to burn, cauterize” (CPArm., JBArm., JPArm., Syr.)		*kawā* (*kwy*) “to caute-rize, burn one’s skin”			*kawû*, *kamû* “to burn (trans.), bake, roast”
1.10	*qd* “to burn, ignite”[12]	*yqd* “to burn”	*yqd* “to burn” (common)	*mqd* “scor-ched, singed”	*waqada* “to burn”	*wqd* “to put a big branch on the fire to make it burn a long time” (Mehri)		*qiādu*, *qâdu* “to ignite”
1.11	**qlw* “to roast, burn” (PS, 2c)	*qlh* (*qly*) “to roast”	*qly* “to roast, burn” (common)		*qalā* (*qlw/qly*) “to fry (wheat or meat)”	*qəlō* (*qlw/qly*) “to cook, fry, annoy, irritate” (Mehri); *qéle* (*qlw/qly*) “to roast, cook” (Soqoṭri)	*qalawa* “to roast, burn”	*qalû* “to burn, roast”

these roots is possible. However, one should be cautioned by the fact that *kbw* "to be quenched, to smoulder" cannot be traced further back than the Proto-Central Semitic (the latest common ancestor of Arabic, Canaanite – including Hebrew – and Aramaic).

[12] The verb *wqd* (> *yqd*) in West Semitic (of which Ugaritic *mqd* is a derived adjective), and the verb *qiādu* (root *qyd*) in Akkadian suggest different extensions of the original 2c root **qd*.

1.12	**qm* "to be(come) hot, burn" (PS, 2c)[13]						*moqa* (*mwq*) "to grow hot, be warm, become intense (heat)"; *maqaqa* "to burn, sting (e.g. medicine), burn the throat, cauterize"	*qamû* "to burn (trans.)"
1.13	**šb*, **śb* "to burn, flare up, emit sparks" (PS, 2c)[14]	*šābîb* "spark of fire" (Aramaic lw.?)	*šəbīb*, *šəbībāʔ* "spark of fire" (BArm., JPArm., Syr., Mnd.); *šbb* "to be		*šabba* "to be youthful, brisk, lively, to burn, blaze"	*śbb* "to climb, flare up (said of fire), grow up (said of youth)" (Mehri); *šebb*	*sehbo* "slow fire"[15]	*šabābu* "to roast, burn (in transferred meanings) (?)"; *šibūbu* "spark"[16]

[13] The 3c verbs *mwq*, *mqq* in Geʕez appear as extensions of original 2c **mq*, and the 3c verb *qamû* (*qmw*?) in Akkadian appears as an extension of original 2c **qm*. Comparative evidence from Western Chadic (Bolewa) suggests that the root **qm* is original, and **mq* (with further extensions) – a metathesis thereof (see *HSED*: no. 349).

[14] The evidence of Arabic and Mehri suggests that the original PS root was **śbb* (derived from 2c **śb*). However, this is not consistent with Hebrew *šābîb*, Aramaic *šəbībāʔ* "spark," and Geʕez *sababa* "to blow up, be joyful" (if the latter is really connected with the other words cited here – cf. the following note). The Hebrew, Aramaic and Geʕez forms suggest the original root **šbb* (derived from 2c **šb*). For a discussion of the problem of etymologically related words in different Semitic languages, which include **ś* as one of the radicals in some languages and a different sibilant in a corresponding position in other languages, see Blau 1998 (1977).

[15] For a proposed etymological connection between Geʕez *sehbo* and common Semitic *šbb* "to burn, flare up," see *CDG*: 492a. If this connection is valid, it appears that Geʕez took a different route to the extension of originally 2c base **šb* to fit the 3c pattern – by addition of *h* as the middle radical rather than by doubling the final radical.

[16] The translation "to roast, burn" for *šabābu* follows *CAD* Š/1: 2b-3a. *AHw*: 1118a-b translates "approximately 'to glow, be parched'" ("etwa 'glühen, verdorren'"). One way or another, the verb expresses some action connected with burning. Concerning *šibūbu* "spark," it has been suggested that it is an Aramaic loanword (*AHw*: 1229b). However, the direction of borrowing may have been reverse: from Akkadian to Aramaic (Abraham and Sokoloff 2011: 53, no. 244). Alternatively, it is possible that both Akkadian *šibūbu* and Aramaic *šəbībāʔ* are indigenous reflexes of an original PS lexeme.

			burned up with sparks" (JBArm.)			"to blow (upon the fire)" (Soqoṭri)		
1.14	**lhb* "to burn, be inflamed, hot" (PS, 3c)	*lahab* "flame"	*lhb* "to be inflamed"; *lhbʾ* "flame" (JPArm.); *šlhb* "to kindle, inflame" (CPArm., JPArm., Syr.)		*lahaba* "to be thirsty"; *lahhaba* "to make the fire flame fiercely, or without smoke"	*ləhēb* "hot wind" (Mehri)	*lahaba*, *lahba* "burn (intrans.), blaze, flame, be warm, perspire"	*laʔbu* "infectious fever (?)"[17]
1.15	**śrp* "to burn" (PS, 3c)[18]	*śrp* "to burn comple-tely"	*śrp* (OffArm.), *srp* (JBArm., JPArm., Sam.) "to burn"	*šrp* "to burn"		*śrf* "to build up sticks for fire" (Mehri)		*šarāpu* "to light a fire, burn, burn up"

[17] This is the translation proposed by *AHw*: 526b ("ein ansteckendes Fieber"). *CAD* L: 34b-35a translates *laʔbu* as "a skin disease" and "spot affected (by *laʔbu*)." However, CAD admits that the Akkadian lexical lists of synonyms explain *laʔbu* as a kind of fever for etymological reasons (*CAD* L: 35a). Thus, the etymological connection between *laʔbu* and West Semitic *lhb* "to burn, be inflamed" is supported by the evidence of the Akkadian lexical tradition.

[18] Egyptian *srf* "(to be) warm" (*WÄS* IV: 195) appears to be a cognate of this lexeme, which means that it can be traced back to PAA.
