# Supplementary material for: Statistics of Language Morphology Change: From Biconsonantal Hunters to Triconsonantal Farmers
Source: PLoS One. 2013 Dec 19;8(12):e83780. doi: 10.1371/journal.pone.0083780 (PMC3868553; doi:10.1371/journal.pone.0083780)
Supplement: Table S2 — Etymological Appendix for Table 2. (PDF) [file pone.0083780.s003.pdf]

Table S2. Etymological Appendix for Table 2 (by Yigal Bloch).

No.	Proto-word	Hebrew	Aramaic	Ugaritic	Arabic	Modern South Arabian	Ethiopic	Akkadian
2.1	**ḥaẓẓ*, **ḥiẓẓ* "arrow" (PS, 2c)	*ḥēṣ* "arrow"	*ḥṣyʾ* "arrows" (OArm.); *ḥṭʾ* "arrow" (OffArm.)	*ḥẓ* "arrow"	*ḥaẓwatun* "a small arrow"		*ḥaṣṣ* "arrow"	*ūṣu, uṣṣu* "arrow, arrowhead"
2.2	**kīs*, **kist* "small bag" (PS, 2c)[19]	*kîs* "bag, purse"	*kīs, kīsāʾ* "small bag, purse, fund" (JArmTg., JBArm., JPArm., Syr.)		*kīsun* "bag for money and precious stones"	*kəst* "bag" (Mehri)	*kis* "purse"	*kīsu* "leather bag for weights and silver, silver capital, treasury"
2.3	**qaš*(*t*) "bow" (PS, 2c)	*qešet* "bow"	*qšat*, *qaštāʾ*, *qeštāʾ* (common), *qšy* (Sam.) "bow"; *kšṭ* "to shoot with a bow" (Syr.)[20]	*qšt* "bow"	*qawsun* "bow"[21]		*qast* "bow"	*qaštu* "bow"

[19] It is commonly accepted that Hebrew *kîs* and Aramaic *kīsāʾ* are loanwords from Akkadian, and that Aramaic was the source for Arabic *kīsun* and Geʿez *kis* (see, e.g., *HALOT*: 472b; *CDG*: 295b). However, Mehri *kəst* "bag" stands out due to the feminine ending -*t*, which is not attested in Akkadian *kīsu* and its alleged borrowings. Thus, whether or not the words in Hebrew, Aramaic, Arabic and Geʿez are loans from Akkadian, Mehri *kəst* appears to be a genuine cognate of Akkadian *kīsu*, and hence the lexeme in question can be reconstructed as PS.

[20] The last verb is evidently denominative from *qšat* "bow," in which the feminine ending -*t* was re-interpreted as the third radical, along with de-emphathization *q* > *k* and emphathization *t* > *ṭ*.

[21] Arabic is unique among the Semitic languages in attesting a 3c base in the noun for "bow" (-*t* in other languages was originally the feminine suffix). It has been suggested that the form *qaws* in Arabic reflects a metathesis of the root *qsw* (PS **qšw*) "to be hard," viz., "hard to bend" (Rundgren 1990: 183-184). Then, both *qaws* and *qsw* < **qšw* would be extensions of originally 2c forms.

2.4	**rmy* "to throw, shoot, lay down" (PS, 2c)	*rmh* (*rmy*) "to throw, shoot"	*rmy* "to throw, set smth. down, move smth. downward" (common)	*rmy* "to throw, shoot (?)"	*ramā* (*rmy*) "to throw, cast, shoot"		*ramaya* "to strike, hit"	*ramû* "to throw, cast, lay down"
2.5	**ṣd* "to prowl, roam, hunt, fish" (PS, 2c)[22]	*ṣwd* "to hunt"; *ṣayid* "game (hunted meat)"	*ṣwd* "to hunt, capture, trap" (common)	*ṣd* "to hunt, scour, traverse"	*ṣāda* (*ṣyd*) "to capture, trap, hunt, fish"	*əṣtəyūd* "to fish" (Mehri)		*ṣâdu* "to prowl, turn about, whirl"; *ṣayyādu* "stalker, stalking hunter"
2.6	**ṣīd* "provisions"; **ṣd* "to provide with food" (PS, 2c)[23]	*ṣayid*, *ṣêdâ* "food, provisions for a journey"	*ṣaydāˀ* (common), *ṣwādāˀ*, *zwādāˀ* (Syr.), *zauada* (Mnd.) "provisions"; *zəwādāˀ* "provisions (esp. for		*zādun*, *zawādun* "provisions for traveling or for a fixed residence"; *zāda* (*zwd*) "to lay in stock provisions	*zəwōd*, *zəwədīn* "supplies"; *azīd* (*zwd*) "to supply, provision" (Mehri)		*ṣidītu* "provisions, travel provisions"; *ṣudû* "provisions"; *ṣuddû* "to provide with food"

[22] It appears that in PS, the verb **ṣd* (**ṣwd*/**ṣyd* in the 3c notation) signified both prowling/roaming and hunting/fishing. The verb retained only the former semantic notion in Akkadian (although the noun *ṣayyādu* retained the notion of hunting), and retained only the latter semantic notion in the West Semitic languages, with the apparent exception of Ugaritic, where the meaning "to scour, traverse (a territory)" (*DULAT*: 778) seems to be a development of the original notion "to prowl, roam."

[23] Some sort of semantic association between these words and the verb **ṣd* "to prowl, roam, hunt, fish" is possible; however, in Proto-Semitic (at the stage of tri-consonantal root morphology), the roots meaning "to prowl, roam, hunt, fish" and "to provide with food" were most likely perceived as different lexical entities, not as different aspects of the meaning of one and the same lexeme. This assumption is based on the fact that in several attested Semitic languages, these roots are treated differently, both in their use as verbs and in relation to the nouns derived from them. Thus, in Akkadian, *ṣidītu* "provisions" and *ṣuddû* "to provide with food" are derived from the 3c root *ṣdy*, whereas the verb *ṣâdu* "to prowl, turn about, whirl" and the noun *ṣayyādu* "stalker, stalking hunter" are derived from the 3c root *ṣwd*/*ṣyd*. In Arabic, the verb "to capture, trap, hunt, fish" is *ṣyd*, whereas "to lay in stock provisions" is *zwd* (it is possible that the first radical in the verb *zwd* had undergone the shift *ṣ* > *z*, from an emphatic to a voiced consonant, under the influence of the voiced dental plosive *d*). In Aramaic also, there is the verb *zwd* "to provide provisions," and the noun *zwādāˀ* "provisions" (beside *ṣaydāˀ* and *ṣwādāˀ*), which may reflect the same shift *ṣ* > *z*; but the verb "to hunt" appears always with *ṣ* as the first radical.

			traveling), outfit for burial" (JArmTg., JBArm.); *zwd* "to provide provisions" (Syr., JPArm.), "to provide outfit for burial" (JBArm.)		for traveling or for a fixed resi-dence"				
