# Supplementary material for: Statistics of Language Morphology Change: From Biconsonantal Hunters to Triconsonantal Farmers
Source: PLoS One. 2013 Dec 19;8(12):e83780. doi: 10.1371/journal.pone.0083780 (PMC3868553; doi:10.1371/journal.pone.0083780)
Supplement: Table S3 — Etymological Appendix for Table 3. (PDF) [file pone.0083780.s004.pdf]

Table S3. Etymological Appendix for Table 3 (by Yigal Bloch).

**No.**	**Proto-word**	**Hebrew**	**Aramaic**	**Ugaritic**	**Arabic**	**Modern South Arabian**	**Ethiopic**	**Akkadian**
3.1	**ˀikkar* “farmer” (PS, 3c)	*ˀikkār* “agricultural worker in servitude, without land” (Akkadian lw.?)	*ˀkr* “to plow, cultivate a field, bear (progeny, fruit)” (Syr.); *ˀikkārāˀ* “farmer” (JArmTg., JBArm., Syr.) (Akkadian lw.?)		*ˀakara* “to till ground, dig a cavity for water in the ground”; *ˀakkārun* “cultivator of land” (Aramaic lws.)		*akkärä* “to renew land by plowing and sowing” (Amharic); *t-akärä* “to build a house and cultivate the field around it for the first time” (Chaha)[24]	*ikkaru* “farmer, plowman”[25]
3.2	**ˀasam*, **ˀisint* “granary, storehouse” (PS, 3c)[26]	*ˀāsām* “granary, storehouse”	*ˀsym, ˀsymˀ* “granary” (Sam.); *ˀassān, ˀassānāˀ* “granary,	*asm* “granary”				*išinnu, iššenu,* “stalk of grain”; *išittu, isittu, esittu,* “treasury,

[24] The verbs in Ethiopian languages are unlikely to be loanwords from either Aramaic or Arabic (Militarev 2002: 146).

[25] Akkadian *ikkaru* is commonly considered a loan from Sumerian ENGAR “farmer,” and the corresponding words in Hebrew, Aramaic and Arabic are considered loans from Akkadian, with the Arabic words borrowed through the mediation of Aramaic (*AHw:* 369a; *CAD* I-J: 49a, 54b; *CDA*: 126a). This reconstruction is possible; however, as pointed out by Militarev, the evidence of the modern Semitic languages of Ethiopia suggests that the verbal root *ˀkr* “to cultivate land” belonged to the PS lexicon (see the preceding note). Thus, whether or not the words derived from this root in Hebrew, Aramaic and Arabic are Akkadian loans, the noun *ikkaru* in Akkadian itself appears to be genuinely Semitic, and Sumerian ENGAR appears to be a loan from Akkadian.

[26] The interchange *m*/*n* is relatively common in Semitic (Lipiński 2001: §11.7). The second radical in the Akkadian forms *išinnu* “stalk of grain” and *išittu* (< **išintu*) “treasury, storehouse” is *š*, which is not a standard correspondence for West Semitic *s*. However, interchange between *s* and *š* is attested in Akkadian – cf. the Babylonian forms of the numerals “seven” and “eight”: *sebe* and *samāne* (*GAG*: §69b); these forms are reflexes of PS **šabˁ* and **ṯamāniy*, although the normal reflex of PS **š* and **ṯ* in Akkadian is *š* (see Lipiński 2001: §35.12-13). In fact, the syllabic spelling *e-si-it-tum* in a Babylonian mathematical text from the first half of the second millennium B.C.E. (*CAD* I-J: 243b, s.v. *išittu*, b) indicates the form *esittum*, with *s* as the second radical.

			storehouse" (JArmTg., JBArm., JPArm.), "provisions" (Syr.)					storehouse"
3.3	*ʕ*inb*, *ʕ*inab* "grape, fruit" (PS, 3c)[27]	*ʕēnāb* "grape(s)"	*ʕinbāʔ* (JArmTg., JPArm., JBArm.,), *ʕenbtāʔ* (Syr.), *ʕnbʔ* (CPArm.) "grape(s)"	*ġnb* "grape, grapes"[28]	*ʕinabatun* "grape"			*inbu* "fruit tree, fruit, offspring, sexual attractiveness"
3.4	**biʔr* "well, pit, cistern" (PS, 3c)[29]	*bəʔēr* "well, pit (for extraction of bitumen or storage	*byrʔ* (OArm.), *bʔr* (OffArm.), *bəʔērāʔ* (JPArm.) "well";	*bir* "well"	*baʔara* "to dig (a well, a hole for cooking), hide, store for a time	*bayr* "well" (Mehri); *ʕébehor* "well, pit" (Soqoṭri)	*barbir*, *bərbir* "cistern, well, pit, latrine"	*būru*, *būrtu* "pit, hole, well, pond"; *berâtu* (pl.) "lagoons (?), swamps (?)"

[27] In West Semitic languages, reflexes of PS *ʕ*inb*, *ʕ*inab* signify uniformly grape(s). However, Akkadian *inbu* has a generic meaning "fruit, fruit tree" (*AHw*: 381b-382a; *CAD* I-J: 144a-147a); the meanings "offspring" and "sexual attractiveness" appear to be derived from that basic meaning. It is impossible to figure out whether the generic meaning "fruit" or the specific meaning "grape(s)" is more original.

[28] Ugaritic *ġnb* belongs to the limited group of words in which Ugaritic *ġ* corresponds to ʕ in other Semitic languages (see Tropper 2000: 126-127).

[29] The second radical ʔ is attested in Hebrew, Ugaritic (spelled with the sign denoting ʔ followed by *i* or a syllable-closing ʔ), as well as in some Aramaic and Arabic forms. The second radical *h* in Soqoṭri *ʕébehor* (where ʕ appears to be a morphological prefix – see *LSoq:* 295) reflects a shift from the voiced glottal ʔ to the homorganic unvoiced glottal *h*. The forms without ʔ in Aramaic and Arabic can be explained as resulting from syncopation of a syllable-closing ʔ: **iʔ* > *ī* or **eʔ* > *ē*. Geʕez *barbir*, *bərbir* reflects reduplication of the nominal base after the radical ʔ had been lost (cf. *bētata* "to persevere, persist, last, stay," derived from *bēta* "to spend the night, stay in a house, dwell," which is in turn derived from the originally 3c root *byt* – see *CDG*: 113a, 116a). Akkadian *būru*, *būrtu* may be derived from PS **biʔr*, with syncopation of ʔ and vowel shift **ī* > *ū* conditioned by the labial consonant *b*: **biʔr* > **bīr* > *būr*. On the other hand, Akkadian *būru*, *būrtu* can also be derived from PS **baʔVr*, **buʔVr* "pit, hole" (see *HSED*: no. 164). It appears that the semantic notions of "well" and "pit, water-cistern" were not fully lexically distinguished in PS – hence their blending in Akkadian, in some instances in Biblical Hebrew (*beʔĕrōt ḥēmār* "bitumen pits" in Gen. 14:10 and *bəʔēr* as a pit used for grain storage in 2 Sam. 17:19), in the 1st-millennium CE Aramaic dialects (Syriac and Jewish Aramaic), and in Geʕez (where the meaning "latrine" appears to be a secondary development from "pit"). In any event, PS **biʔr* appears to have denoted water reservoirs, and may have had a more generic meaning "pit," but probably did not refer specifically to storage pits, evidence for which is limited to reflexes of PS **biʔr* in Hebrew and Arabic only.

		of grain)"	*bēr*, *bērāˀ* (JArmTg., JBArm., JPArm.,), *bēˀrāˀ*, *bīrāˀ* (Syr.) "well, pit, grave"		of need"; *biˀrun*, *bīrun* "well"; *baˀˀārun* "digger of wells"; *baˀīratun* "a thing stored"			
3.5	**bšl* "to ripen, be cooked" (PS, 3c)[30]	*bšl* "to ripen, boil" (basic stem), "to cook" (doubled stem)	*bšl* "to ripen, be cooked" (basic stem), "to cook, boil" (doubled stem) (common)	*bšl* "to cook" (basic stem? doubled stem?)[31]	*basala* "to become strong (in taste or odor), sour (said of food)" (basic stem), "to cook unripe dates" (causative stem)	*bəhēl* "to be cooked, baked, ready" (basic stem), "to cook, prepare" (causative stem) (Mehri); *béhel* "to be cooked" (Soqoṭri)[32]	*basala* "to be cooked, be ripe" (basic stem), "to cook" (causative stem)	*bašālu* "to be cooked, ripen" (basic stem), "to cook" (causative stem)

[30] In all the attested Semitic languages, reflexes of PS **bšl* have the intransitive meaning "to ripen, be cooked" in the basic stem (i.e., the verbal stem that is declined without addition of consonantal prefixes or infixes, other than those expressing the grammatical person, and without lengthening of the vowels that appear between the root consonants). The transitive meaning "to cook" is reserved for the reflexes of PS **bšl* in the doubled stem (declined with the doubling of the middle radical, in all grammatical forms) and in the causative stem (declined with the addition of the consonantal prefix *š*, *h* or *ˀ*). The fact that no attested language uses a reflex of **bšl* in the doubled or causative stem with the meaning "to make smth. ripen" (related to floral products still in the stage of their growth), and the intransitive meaning of Arabic *basala* "to become strong, sour" (a state of food reached without a necessary human intervention) suggest that "to ripen, overripen" was the basic meaning of PS **bšl* (or its pre-PS ancestor). The meanings associated with cooking – both intransitive and transitive – appear to have resulted from later semantic developments.

[31] Comparison with other languages suggests that Ugaritic *bšl* "to cook" (transitive) is in the doubled stem. Given the relative scarcity of Ugaritic textual evidence, the absence of intransitive *bšl* "to ripen" in Ugaritic is not surprising.

[32] For the consonant shift **š* > *h* in Mehri, and in some instances, in Soqoṭri, see Lonnet and Simeone-Senelle 1997: 346-347, 361.

3.6	**buṭm*, *buṭn* "pistachio, terebinth" (PS, 3c) [33]	*bōṭnîm* (pl.) "pistachios"	*buṭmāˀ*, *buṭnāˀ* (JArmTg., JPArm., JBArm.), *beṭmətāˀ* (Syr.) "terebinth, pistachio"		*buṭmun* "terebinth, terebinth nuts (coll.)" (Aramaic lw.)		*bəṭm*, *buṭm* "terebinth tree" (Arabic lw.)	*buṭnu* "terebinth, (tree and nuts)"; *buṭuttu*, *buṭumtu*, *buṭuntu* "pistachio tree, wood, nut"
3.7	**duḫn* "millet" (PS, 3c)[34]	*dōḥan* "millet"	*duḥnāˀ* "millet" (Syr.)		*duḫnun* "millet"			*duḫnu* "millet"
3.8	**ḍaˀn* "small livestock (sheep and goats)" (PS, 3c)	*ṣōˀn*, *ṣōnê* "flocks (sheep and goats)"	*qn* (*ǧn*) (OffArm.), *ˁān, ˁānāˀ* (JPArm., Syr.) "small livestock, sheep"	*ṣin* "ewe, small livestock"	*ḍaˀnun* "sheep"			*ṣēnu*, *ṣānu*, *ṣeˀānu* "sheep and goats"
3.9	**gurn*, **garīn*, **magrān* "place for storing produce, threshing floor" (PS, 3c)	*gōren* "threshing floor"	*grnˀ* "threshing floor" (Sam., Hebrew lw.)	*grn* "threshing floor"	*ǧarana* "to grind grain vehemently"; *ǧurnun* "a stone mortar"; *ǧarīnun* "what one has ground (of grain), place where dates are dried or		*gwərn*, *gorn*, *gurn* "threshing floor"	*garānu*/ *qarānu* "to store, pile up in heaps"; *magrānu*/ *maqrānu* "grain pile, threshing floor"; *magrattu*/ *maqrattu* "threshing floor"[36]

[33] For the interchange *m*/*n*, see above, n. 26.

[34] PS **duḫn* "millet" is suggested to derive from the Arabic verb *daḫana*, "to give smoke" (*HALOT*: 218b). However, this verb is not attested elsewhere in Semitic (*DRS*: 250), and hence is not likely to be reconstructed back to PS.

					wheat is trodden out”[35]				
3.10	**hugār* “meadow, field, arable land” (PS, 3c)[37]			*ugr* “field, soil” *ugrt* “Ugarit (topo-nym)”				*garh, garāht* “field, arable land, farm”; *garha* “to plow”	*ugāru* “grass-land, meadow, arable land”
3.11	**ḥaql* “field” (PS, 3c)		*ḥaqlāˀ* “field” (common)		*ḥaqlun* “field, land lacking trees, cereals at an early stage of growth”			*ḥaql* “field, plain, desert, country-side”	*eqlu* “field”

[36] The cuneiform spellings of the nouns *magrānu/maqrānu*, *magrattu/maqrattu*, and of the different forms of the verb *garānu/qarānu*, do not allow to figure out whether the first radical is *g* or *q*. However, the etymological connection with the West Semitic reflexes of **gurn*, **garīn* “place for storing produce, threshing floor” appears more compelling than the connection with Arabic *qrn* “to tie, bind together,” suggested by W. von Soden (*AHw*: 902a).

[35] The Arabic forms cited here refer mostly to grinding, rather than threshing, of grain, which appears to be a later semantic development. However, one of the meanings of the noun *ǧarīnun* is “place where dates are dried,” and another meaning is “place where wheat is trodden out.” The latter two meanings fit the data from other Semitic languages, indicating that nouns derived from the root *grn* served as designations for places in which agricultural produce was stored in heaps and underwent initial processing.

[37] Geʕez **garh*, *garāht* indicate the glottal voiceless *h* as a radical. Postulating an etymological connection between the Geʕez forms, Akkadian *ugāru* and Ugaritic *ugr* (as proposed by Militarev 2002: 144) requires one to assume metathesis of the root consonants and interchange between ˀ and *h* (glottal voiced and voiceless consonants, respectively). Both assumptions are acceptable. Since Ugaritic and Akkadian belong to two different main branches of the Semitic language family (West and East Semitic, respectively), it appears that the order of the radicals and the vowel pattern attested in these two languages is closer to the form that assumedly existed in PS, and the Geʕez forms are secondary. In any event, it is unlikely that Geʕez *garh, garāht* can be etymologically connected with Arabic *qarāḥ*, *qirwāḥ* “land without trees” (as suggested in *DRS:* 184): *h* and *ḥ* are not homorganic consonants, and there is no regular sound shift in either Geʕez or Arabic, leading from one of them to the other (reservation to this effect is expressed already in *CDG*: 202b). Rather, Arabic *qarāḥ* appears to derive from the PWS root *qrḥ* “to be bald” (see *HALOT*: 1140a; *CDG*: 441a).

3.12	**ḥrṯ* "to plow" (PS, 3c)	*ḥrš* "to plow"	*ḥrt* "to dig, cut in pieces, incise, plow" (Syr.)	*ḥrṯ* "to plow, till, farm the land"	*ḥaraṯa* "to plow"		*ḥarasa* "to plow"	*erēšu* "to seed (using a plow), to cultivate a field"
3.13	**ḫamr* "fermenting wine" (PS? PWS?, 3c)[38]	*ḥemer* "(fermenting) wine"	*ḥəmar*, *ḥamrā*ˀ "wine" (common)	*ḫmr* "(fermenting?) wine"	*ḫamrun* "wine"; *ḫamara* "to become changed"	*ḥámer* "wine" (Soqoṭri)	*ḫamra* "to become sour" (Tigrinya)	*ḫammurtu* "a kind of beer" (Aramaic lw.?)[39]
3.14	**ḫimˀat* "cream, curd, butter" (PS, 3c)	*ḥemˀâ* "cream, butter"	*ḥmˀh* "curd" (Sam.); *ḥeˀwtā*ˀ "butter" (Syr., Akkadian lw.)[40]	*ḫmat* "butter, curd"		*ḥámi* "butter" (Soqoṭri)		*ḫimētu*, *ḫimātu* "butter, ghee"
3.15	**kapr* "village" (PS, 3c)	*kəpār* "open village"	*kaprā*ˀ "village" (JPArm., CPArm., Sam., Syr.)	*kpr* "village"	*kafrun* "village" (Aramaic lw.)			*kapru* "village in an open country, farm"
3.16	**karm*, **karān* "vine, vineyard" (PS, 3c)[41]	*kerem* "vineyard"	*krm* (OffArm.), *karmā*ˀ (JPArm., JBArm.,	*krm* "vineyard"	*karmun* "vine, vineyard"	*karmáym* "mountain" (Mehri)[42]	*karm*, *kərm* "vine, vineyard"	*karānu* "wine, grapevine, grapes"

[38] If the noun *ḫammurtu* "a kind of beer" is original in Akkadian, it will be justified to reconstruct **ḫamr* as a PS lexeme. If Akkadian *ḫammurtu* is an Aramaic loanword (which is not unlikely – see the following note), one can reconstruct **ḫamr* "fermented wine" only back to PWS.

[39] This term appears only in Assyrian documents of the 1st millennium B.C.E. (*CAD* Ḫ: 69b; *AHw*: 318a), and may be an Aramaic loanword, although *AHw*: 318a suggests (with a question mark) derivation from the Akkadian verb *ḫamāru* "to become dry."

[40] Syriac *ḥeˀwtā*ˀ is a loan from Akkadian *ḫimētu*, with the *m*/*w* interchange characteristic of the Babylonian dialect from the mid-2nd millennium onwards (Kaufman 1974: 55-56; cf. *GAG*: §31a).

[41] For the interchange *m*/*n*, see above, n. 26. Reconstruction of the proto-form **karān* is based on Akkadian *karānu*. However, a similar form had probably existed in West Semitic languages spoken in Canaan in the late 4th millennium B.C.E. In Egyptian,

			Syr.) “vine-yard”; *karmāˀ* (Syr.) “vine”				(Arabic lw.?)	
3.17	**labin*(*a*)*t*, **libint* “brick” (PS, 3c)[43]	*ləbēnâ* “brick”	*lēbnā*, *ləbēntāˀ*, *ləbēttāˀ* “brick” (common)	*lbnt* “brick”	*labinat* “brick” (Aramaic lw.?)			*libittu* “brick”
3.18	**nāqid* “shep-herd” (PS, 3c)	*nōqēd* “shep-herd, sheep-breeder”	*nāqdāˀ* “shep-herd”	*nqd* “chief shep-herd”	*naqqād* “a shepherd who tends the kind of sheep called *naqd* (ugly sheep with wool of a good quality)”			*nāqidu* “herds-man”
3.19	**palg* “water-course” (PS, 3c)[44]	*peleg* “artificial water channel,	*plg* “canal” (OffArm., Akkadian	*plg* “stream, canal”	*falağun*, *fuluğun* “river, rivulet,	*fəlēg* “water-course (not arti-	*falaga* “to flow, cause to flow in	*palgu* “canal, irrigation ditch”

the form *k3nw* “garden, vineyard” (grammatically plural) is attested since the mid-3rd millennium B.C.E. (*WÄS* V: 106). This form is an exact parallel of Akkadian *karānu* (for the correspondence between Egyptian *3* and Semitic *r*, see *EDE* 1: 50-61). However, Egyptian *k3nw* cannot be a genuine cognate of Akkadian *karānu*, since grapes do not naturally grow in Egypt (see McGovern 2003: 85), and it would be difficult to assume that a term for “vineyard” was inherited by Egyptian from PAA and preserved for millennia in the absence of actual vineyards in Egypt. Archaeological data indicate that viticulture was probably introduced into Egypt from Canaan in the late 4th millennium B.C.E. (McGovern 2003: 95-103), and it appears that the word *k3nw* “vineyard” was borrowed from Canaanite **karānū* (plural, base **karān*) at about the same time. Interestingly, from the 13th century B.C.E. onwards, another term for “garden, vineyard” appears in Egyptian: *k3m* (*WÄS* V: 106). This form corresponds phonologically to **karm*, which can be reconstructed for PWS based on the forms actually attested in known West Semitic languages (but whose attestations date only from the 14th century B.C.E. onwards).

[42] Etymological connection between Mehri *karmaym* “mountain” and reflexes of PS **karm*, **karān* “vineyard” (as exemplified by Hebrew *kerem*) was proposed by Müller 1985: 272. This connection may be based on the fact that the main regions of vine-growing in pre-Islamic Arabia were “hills and mountains” (Unwin 1996: 128).

[43] -(*a*)*t* in **labin*(*a*)*t*/**libint* is the feminine ending. As noted by Kaufman 1974: 66, and n. 178, “There is no compelling reason to assume that Akkadian is the origin of the common Semitic term and its related forms,” and “It is, in fact, difficult to account for the derivation of the Heb[rew] form. . . from any of the Akkadian forms” (contra *HALOT*: 518a).

		canal”	lw.)		running stream of water (for irrigation)”[45]	ficial)” (Mehri)	torrents, dig out, divide, split”; *falag* “river, brook, valley”	
3.20	**qamḥ* “vegetal food/ drink, flour” (PS, 3c)[46]	*qemaḥ* “flour”	*qamḥāʔ* “fine flour” (common)	*qmḥ* “flour”	*qamḥun* “full-grown wheat, grain of wheat”; *qamiḥa* “to eat (parched barley or wheat), drink”		*qamḥa* “to eat grain or fodder, graze”[47]	*qēmu* “flour, powder (of plants, nuts, etc.)”; *qamû*, *qemû* “to grind, pulverize”[48]
3.21	**rahṭ* “drinking trough” (PS, 3c)	*rahaṭ* “drinking trough” (Aramaic lw.?)	*rahṭāʔ* “drinking trough” (JPArm., Syr.)					*rāṭu* “water-channel, runnel”

[44] In verbal usage, the common Semitic root *plg* (*plk* in Akkadian *palāku*) means “to split, divide.” The noun **palg*, signifying a stream of water, appears to have developed from this root (see *CDG*: 159a). Although in individual Semitic languages, reflexes of **palg* can signify either a natural or an artificial stream of water (or both), the digging of an artificial canal would actively divide a given plot of land in two parts. Hence, it seems more likely that PS **palg* originally designated an artificial canal, and came to be used for designating natural water streams only later.

[45] With regard to an irrigation system whose name in Arabic is derived from the root *flǧ*, compare the *Aflaj* (*ʔaflaǧ*, “channels”) system in Oman, a World Heritage site that dates ca. 500 C.E. (http://www.worldheritagesite.org/sites/aflaj.html).

[46] The data from Geʕez and Akkadian (where *qēmu* was apparently used to signify powder of different vegetal substances beside grain – see *CAD* Q: 208), as well as the meaning “to drink” attested for the Arabic verb *qamiḥa*, suggest that PS **qamḥ* had a generic meaning “vegetal food/drink.” This meaning would presumably exist in PS beside the specific meaning “flour” (in the sense of powder obtained by grinding grain), which is attested in Hebrew, Aramaic and Ugaritic.

[47] In Geʕez, there is also the verb *qamḥa* “to produce fruit,” which appears to be denominative from *qamḥ* “produce, yield, fruit, leguminous plant.” In *CDG*: 431b-432a, *qamḥa* “to produce fruit,” and the noun from which it is derived, are separated from *qamḥa* “to eat grain or fodder, graze,” and only the latter verb is etymologically connected with Hebrew *qemaḥ*, Arabic *qamḥun*, etc.

[48] The Akkadian verb *qamû*, *qemû* “to grind, pulverize” appears to be denominative from *qēmu* “flour.”

3.22	**š*ʔ*b* "to draw water"	*šʔb* "to draw water"	*šʔb* "to draw out, absorb" (JBArm., JPArm.)	*šʔb* "to draw or carry water"	*saʔaba* "to be satisfied with drinking"; *saʔbun* "a leather receptacle for wine, honey, butter"		*saʔaba* "to drag, pull, follow"	*sâbu, sâpu* "to draw water"[49]
3.23a	**šikar* "beer, intoxi-cating drink" (PS, 3c)	*šēkār* "beer, intoxi-cating drink"	*šakrāʔ* "intoxica-ing drink" (CPArm., JBArm., Syr.)		*sakarun* "wine, fermented date juice"			*šikaru, šikru* "beer, alcoholic drink"
3.23b	**škr* "to be(come) drunk" (PS, 3c)	*škr* "to be(come) drunk"	*škr* "to be drunk" (CPArm., JBArm., Syr.)	*škr* "to become intoxi-cated"	*sakira* "to become intoxi-cated"	*sīkər* "to be drunk" (Mehri; Arabic lw.?)[50]	*sakra* "to be drunk, intoxi-cated"	*šakāru* "to be(come) drunk"
3.24	**šlq* "to boil, cook" (PS, 3c)[51]	**šlq* "to boil, seethe, dissect" (post-Biblical)	**šlq* "to cook, cleanse by boiling" (JBArm., JPArm., Syr.)		*Salaqa* "to boil, cook, remove meat or hair with hot water"		*šäläqä* "to be burned, to simmer" (Tigri-nya)	**salāqu* "to boil, cook"

[49] For the irregular correspondence of Akkadian *s* to West Semitic (and probably Proto-Semitic) *š, see above, n. 26.

[50] PS *š in Mehri normally shifted to *h*, and in some rare instances was retained (Lonnet and Simeonne-Senelle 1997: 346-347, 361). In Mehri *sīkər* "to be drunk," the correspondence of *s* to PS *š (where the PS consonant is assured by the evidence of Hebrew, Aramaic, Ugaritic and Akkadian) suggests that the Mehri word is a loan from Arabic.

[51] The attestation of *š* as the first radical in Hebrew, Aramaic and Tigrinya suggests that this was the first radical also in PS. Akkadian *salāqu*, with *s* as the first radical, appears to reflect an irregular sibilant correspondence (cf. above, n. 26).

3.25	**šṭl* "to plant" (PS, 3c)[52]	*šṭl* "to plant"	*šṭl* "to plant" (JBArm., Syr., Mnd., CPArm.); *šeteltāˀ* (Syr.), *šitlāˀ* (JBArm.), "plant"		*šatlun* "plant, sapling, young wood"; *šatlatun* "sapling, stalk of a plant"	*šílil* "to plant" (Soqoṭri)[53]		*satālu* "to plant"; *šitlu* "offshoot, sprout"[54]
3.26	**tibn* "straw, chaff" (PS, 3c)	*teben* "straw, chaff"	*tibnāˀ*, *tebnāˀ* "straw" (common)		*tibnun* "straw" (Aramaic lw.)			*tibnu* "straw, chaff"
3.27	**zrʕ* "to sow" (PS, 3c)[55]	*zrʕ* "to sow"; *zeraʕ* "seed, offspring"	*zrʕ* "to sow" (common)	*drʕ* "to sow, scatter"; *drʕ*, *ḏrʕ* "seed-grain, sowing"	*zaraʕa*, *ḏaraˀa* "to sow, scatter"		*zarʕa*, *zarˀa* "to sow, scatter seed"	*zarû* "to sow, scatter, winnow"; *zēru* "seed"

[52] The correspondence between Hebrew and Aramaic *š*, on the one hand, and Arabic *š* (rather than *s*), on the other hand, is problematic. Yet, the evidence of Hebrew and Aramaic suggests that PS **š* is the original first radical of the root under consideration. Perhaps Arabic *šatlun*, *šatlatun* (not attested in Classical Arabic – see *BDB*: 1060a; *SDA* I: 727a-b) are to be explained as Aramaic loans.

[53] The Soqoṭri verb appears to result from a re-analysis of the 3c root *štl*, whereby the middle radical *t* was interpreted as the morphological infix of a verbal stem, and thus excluded from the root (*LSoq*: 417).

[54] The verb *satālu* "to plant" appears only in texts composed in Assyria, viz., in the royal inscriptions of Sennacherib, 705-681 B.C.E. (see *AHw*: 1033a; *CAD* S: 197b). Hence, the spelling with a cuneiform sign indicating *s* as the first radical appears to be due to the fact that in the Assyrian dialect of the 1st millennium B.C.E. PS **š* had developed into *s* (Lipiński 2001: §15.2). The noun *šitlu* "offshoot, sprout," attested in Babylonian lexical lists of the 2nd and the 1st millennium B.C.E., features the standard Akkadian reflex of PS **š*.

[55] The forms with *ḏ* or a reflex thereof as the first radical (i.e., Ugaritic *drʕ*, *ḏrʕ* and Arabic *ḏaraˀa*) are likely to have been generated under the influence of the PS verb **ḏrw* "to scatter, spread, winnow" and its reflexes in the attested languages (Blau 1998 [1977]: 67-68). In Akkadian, *zarû* has both the meaning "to sow" and "to winnow," the latter being restricted to the Babylonian, and the former – almost entirely to the Assyrian dialect (see *AHw*: 1516b; *CAD* Z: 70b-71b). Although for an Akkadian verb derived from PS **zrʕ* the vowel shift **a* > *e* would be expected, its non-occurrence in *zarû* is not a conclusive argument that this verb is derived from PS **ḏrw*.
