# Supplementary material for: Statistics of Language Morphology Change: From Biconsonantal Hunters to Triconsonantal Farmers
Source: PLoS One. 2013 Dec 19;8(12):e83780. doi: 10.1371/journal.pone.0083780 (PMC3868553; doi:10.1371/journal.pone.0083780)
Supplement: Table S4 — Etymological Appendix for Table 4. (PDF) [file pone.0083780.s005.pdf]

Table S4. Etymological Appendix for Table 4 (by Yigal Bloch).

No.	Proto-word	Hebrew	Aramaic	Ugaritic	Arabic	Modern South Arabian	Ethiopic	Akkadian
4.1	**ˀmr* "to see, show, say" (PS, 3c)[56]	*ˀmr* "to say"	*ˀmr* "to say, command" (common)	*ˀmr* "to look at smth."; *amr* "order, demand, word (?)"	*ˀamara* "to command"	*ˀāmər* "matter, order" (Mehri)	*ˀammara* "to show, indicate, tell"	*amāru* "to see"
4.2	**hwy*, **hyy* "to be, become (smth. else), fall" (PS, 2c)	*hyh* (*hyy*) "to be"	*hyy* "to be, endure, come to pass" (common)		*hawā* (*hwy*) "to make smth. fall down"	*həwū* "to fall, swoop" (Mehri)	*tahayaya* (*hyy*) "to be free from care, disregard, overlook"	*ewû* "to change, turn into smth."
4.3	**ˁśw* "to do, work, (mis)treat smb." (PS, 2c)[57]	*ˁśh* (*ˁśy*) "to do, make"		*ˁšy* "to make, process, work, do smth. (bad)"	*ˁašā* (*ˁšw*) "to be or pretend to be blind, to treat smb. wrongly"			
4.4	**bwˀ* "to come, enter, return" (PS, 2c)	*bwˀ* "to come"		*bˀ* "to enter, come, reach"	*bāˀa* (*bwˀ*) "to return"		*boˀa* "to enter, penetrate, proceed"	*bâˀu* "to walk along, go through, come in"

[56] Assuming that the original meaning of PS **ˀmr* was "to see," and that the other meanings developed through a semantic shift ("to see" > "to show" > "to say" > "to command") seems the most economic way to account for the spectrum of the attested meanings of this verb in the Semitic languages (see Albright 1954: 229, n. 47; Rundgren 1963: 181-182). For an alternative proposal, assuming that the basic meaning of **ˀmr* in PS was "to stand out, be conspicuous, clear, bright," see *CDG*: 25b, and the earlier studies cited there.

[57] In Semitic, reflexes of the verb **ˁśw* are limited to the Central Semitic branch. However, reflexes of the 2c base **ˁś* "to do, make" are attested in Cushitic and Chadic languages (*HSED*: no. 1097). This suggests that the verb **ˁś* belongs to the PAA stratum, and therefore, **ˁśw* (extended to fit the 3c pattern) must have existed in PS.

4.5	**ntn* “to give” (PS, 2c)	*ntn* “to give”	*ntn* “to give” (common)	*ytn* “to give, direct (one’s face), raise (one’s voice)”			*natana* “to give”	*nadānu* “to give”
4.6	**hlk* “to go” (PS, 3c, but probably derived from 2c **lk*)[58]	*hlk* “to go”	*hlk* “to go, proceed, behave, die” (OffArm., BArm., JPArm., Sam.)	*hlk* “to go, run, flow, roam”	*halaka* “to perish”	*hlk* “to perish” (Soqoṭri)		*alāku* “to go”
4.7	**rˀy* “to see” (PWS, 2c)	*rˀh* (*rˀy*) “to see”			*raˀā* (*rˀy*) “to see, suppose, consi-der, opine”	*rāy*, *rey* “view, opinion” (Mehri, Soqoṭri); *mére* “mirror” (Soqoṭri)	*rəˀya* “to see, look at, contem-plate”	
4.8	**šmˁ* “to hear, listen” (PS, 3c)	*šmˁ* “to hear, listen, obey, under-stand”	*šmˁ* “to hear, listen, obey, under-stand” (common)	*šmˁ* “to hear, listen, notice”	*samiˁa* “to hear, listen”		*samˁa* “to hear, listen, give heed, obey”	*šemû* “to hear, learn, pay attention, obey”

[58] Both Hebrew *hlk* and Akkadian *alāku* are conjugated, in some forms of the basic stem, as though the verbal root were **wlk* (GKC: §69x; *GAG*: §97n). This suggests derivation from the original 2c base **lk*, extended in different ways to fit the 3c pattern. Some measure of support for this hypothesis can be drawn from the parallel use of another verb, *hwk*, for “to go” in Aramaic (see *HALOT*: 1859b-60a). The existence of **hwk* at least as early as in PWS is supported by Geˁez *hoka* “to stir, move, agitate” (*CDG*: 220a). It seems likely that PS **hlk* was a blending of two originally separate 2c bases: **lk* and **hk*.

4.9	**dbr* "to consider the results of an affair, mediate, speak" (doubled stem) (PCS, 3c)[59]	*dbr* "to speak" (doub-led stem)		*dbr* "to say, declare" (doub-led stem)	*dabara* "to follow behind some-one's back, follow or succeed smb."; *dabbara* "to consider the results of an affair, mediate, under-stand, plan"; *duburun* "back"	*adōbər* "to turn the back" (Mehri)	*tadabbara* (*dbr*) "to lie on one's back"	
4.10	**yṯb* "to sit, dwell" (PS, 2c)	*yšb* "to sit, dwell"	*ytb* "to sit, dwell" (common)	*yṯb* "to sit, down, install oneself, reside"	*waṯaba* "to leap, jump, sit down"		*ʾawsaba* "to take a wife, marry"	*wašābu* "to sit, reside, be settled"
4.11	**wḍʾ* "to go out, come forth, rise (in the sky),	*yṣʾ* "to go out, come forth"	*yʿy* "to sprout, blossom" (JArmTg. JPArm.,	*yṣʾ* "to go out, appear"		*ʾeḍa* (*ʿḍy*) "grow (said of herbs)" (Soqoṭri)	*waḍʾa*, *waṣʾa* "go out, go forth, depart, rise (said	*waṣû* "to go out, leave, rise (in the sky), sprout"

[59] The etymology of *dbr* "to speak" in Hebrew and Ugaritic (in the doubled stem) poses a problem. Apparently, the verb **dbr* in PWS signified different actions connected with one's back or rear side (as can be seen from the evidence of Geʿez, Mehri and the basic stem in Arabic). From this basic meaning, through a semantic shift ("rear side" > "later phase of an affair" > "outcome"), the verb **dbr* acquired the meaning "to consider the results of an affair, mediate, plan," as attested in the double stem in Arabic. "To speak" in Hebrew and Ugaritic (in the double stem) appears to be a further extension of this meaning, based on the notion of speech as an instrument of interpersonal mediation. The meaning of *dbr* connected with mediation, planning and speech is attested only in Central Semitic languages, hence the proto-verb **dbr* with this meaning can be reconstructed only on the level of PCS.

	sprout" (PS, 2c)		Syr.)				of sun)"	
4.12	**ṯwb* "to turn back, return" (PWS, 2c)	*šwb* "to turn back, return, repent"	*twb* "to return, turn about, regret" (common, written *šwb* before ca. 500 BCE)	*ṯb* "to turn around, return, repeat, answer"	*ṯaba* (*ṯwb*) "to return, repent, return to one's health"	*ṯwūb* "to requite smb." (Mehri)	*soba* "to turn, turn around, wrap around"	
4.13	**lqḥ* "to take" (PS, 3c)	*lqḥ* "to take, seize, accept"	*lqḥ* "to take, accept (favo-rably, collect)"	*lqḥ* "to take hold of smth., take, grasp, obtain"	*laqiḥa* "to conceive (said of a female), become fecund-dated (said of land)"	*lūqəḥ, līqəḥ* "to conceive (said of a she-camel)" (Mehri)	*laqqəḥa* "to lend"	*leqû* "to take, accept, assume responsi-bility"
4.14	**ydˁ* "to know" (PS, 2c)	*ydˁ* "to notice, learn, know (also sexu-ally)"	*ydˁ* "to know, perceive, cohabit with a woman" (common)	*ydˁ* "to know, reco-gnize, distin-guish"		*wīda* (*wdˁ*) "to know" (Mehri);	*ˀaydəˁa* "to make know, inform, announ-ce"	*idû, edû, wadû* "to know, be experi-enced, familiar with smth."
4.15	**ˁlw* "to ascend" (PS, 2c)	*ˁlh* (*ˁly*) "to go up"	*ˁly* "raise, exalt, bring back, evaluate" (doubled stem) (JArmTg., JBArm.,	*ˁly* "to go up, rise, attack"	*ˁalā* (*ˁlw*) "to be(come) high, elevated, ascend"	*ˀālēw* (*ˁlw*) "at the top" (Mehri); *ˁélhe* "high" (Soqoṭri)		*elû* "to up (to a higher ground or a more important locality, rise, grow, emerge"

			JPArm., Syr.)					
4.16	**šlḥ* “to stretch out, throw, send” (PS, 3c)	*šlḥ* “to stretch out, let free, send”	*šlḥ* “to send, stretch out, put out” (common)	*šlḥ* “to stretch, throw, send, give”		*hlˁ* (causative *šalaḥ*) “to cast a shadow” (Soqoṭri)		*šalû* “to whirl up, kick up dust, toss, spit, shoot arrows”[60]
4.17	**mwt* “to die” (PS, 2c)	*mwt* “to die”	*mwt* “to die” (common)	*mt* “to die, remain immobi-lized, lie aground (?)”	*māta* (*mwt*) “to die”	*mōt* “to die” (Mehri)	*mota* “to die”	*mâtu* “to die”
4.18	**ˀkl* “to eat” (PS, 3c)	*ˀkl* “to eat, devour”	*ˀkl* “to eat, consume” (common)	*ˀkl* “to eat, devour, consu-me, make use of smth.”	*ˀakala* “to swallow food after chewing, eat, consume”		*ˀəkl* “food, bread, grain, fodder”	*akālu* “to eat, consume, provide for oneself”
4.19	**qrˀ* “to call, shout, invite” (PS, 3c)	*qrˀ* “to call, give a name, shout, announ-ce”	*qry* “to call, read, cry out, proclaim” (common)	*qrˀ* “to call, shout, invite, invoke”	*qaraˀa* “to collect, put together, read and recite a book”			*qerû* “to invite, take along, lead away”

[60] For a proposed etymological connection between Akkadian *šalû* “to whirl up, kick up dust,” etc., and West Semitic *šlḥ* “to stretch out, throw, send,” see *DULAT*: 816.

4.20	**nśˀ*<br>“to rise, be(come) elevated, lift up, wear, carry” (PS, 2c)	*nśˀ*<br>“to carry, lift up, raise”	*nśˀ*<br>“to lift up, take away” (OArm., OffArm., BArm., JPArm.)	*nšˀ*<br>“to raise, lift, load with smth., wear”	*našaˀa*<br>“to live, rise, become elevated, grow up”	*miśiˀoh*<br>“bucket”	*naśˀa*, *nasˀa*<br>“to take, partake, receive, capture, pick up, raise, carry off”	*našû*<br>“to lift, take up, wear, carry, bring, accept, receive”
