# Supplementary material for: Statistics of Language Morphology Change: From Biconsonantal Hunters to Triconsonantal Farmers
Source: PLoS One. 2013 Dec 19;8(12):e83780. doi: 10.1371/journal.pone.0083780 (PMC3868553; doi:10.1371/journal.pone.0083780)
Supplement: Text S2 — List of references for the Etymological Appendix. (PDF) [file pone.0083780.s006.pdf]

References (for the Etymological Appendix)

Abraham, K. and Sokoloff, M. 2011. Aramaic Loanwords in Akkadian – A Reassessment of the Proposals. *Archiv für Orientforschung* 52: 22-76

AEL = Lane, E. W. 1968 (1863-1893). *An Arabic-English Lexicon.* Beirut: Librarie du Liban

AHw = von Soden, W. 1956-1981. *Akkadisches Handwörterbuch*. Wiesbaden: Harrassowitz

Albright, W. F. 1954. Northwest-Semitic Names in a List of Egyptian Slaves from the Eighteenth Century B.C. *Journal of the American Oriental Society* 74: 222-233

BDB = Brown, F., S. R. Driver, and C. A. Briggs. 1906. *A Hebrew and English Lexicon of the Old Testament.* Boston: Houghton, Mifflin & Co.

BK = de Biberstein Kazimirski, A. 1846. *Dictionnaire arabe-français contenant toutes les racines de la langue arabe, leurs derives, tant dans l'idiome vulgaire que dans l'idiome littéral, ainsi que les dialects d'Alger et de Maroc*. Paris: Barrois

Blau, J. 1998 (1977). "Weak" Phonetic Change and the Hebrew *śîn*. In: J. Blau, *Topics in Hebrew and Semitic Linguistics*. Jerusalem: Magnes. Pp. 50-103

CAD = Gelb, I. J. *et al.* (eds.). 1956-2011. *The Assyrian Dictionary of the Oriental Institute of the University of Chicago.* Chicago: Oriental Institute

CAL = Kaufman, S. et al. n.d. *Comprehensive Aramaic Lexicon.* Cincinnati: Hebrew Union College. Available online: http://cal1.cn.huc.edu/

CDG = Leslau, W. 1987. *Comparative Dictionary of Geʿez (Classical Ethiopic)*. Wiesbaden: Harrassowitz

Civil, M. 1973. Notes on Sumerian Lexicography, II. *Journal of Cuneiform Studies* 25: 171-177

Del Olmo Lete, G. 2004. *Canaanite Religion according to the Liturgical Texts of Ugarit* (tr. W. G. E. Watson). Winona Lake, In.: Eisenbrauns

Diakonoff, I. 1998. Earliest Semitic society linguistic data. *Journal of Semitic Studies* 43: 209-219

DRS = Cohen, D. (continued by J. Lentin, F. Bron and A. Lonnet). 1970-2012. *Dictionnaire des racines sémitiques ou attestées dans les langues sémitiques.* Fascicules 1-10. Leuven: Peeters

DULAT = del Olmo Lete, G. and J. Sanmartín. 2003. *A Dictionary of the Ugaritic Language in the Alphabetic Tradition* (Handbuch der Orientalistik I/67). Tr. by W. G. E. Watson. Leiden: Brill

EDE 1 = Takács, G. 1999. *Etymological Dictionary of Egyptian*, 1: *A Phonological Introduction* (Handbuch der Orientalistik I/48, 1). Leiden: Brill

EDE 2 = Takács, G. 2001. *Etymological Dictionary of Egyptian*, 2: *b-, p-, f-* (Handbuch der Orientalistik I/48, 2). Leiden: Brill

EDE 3 = Takács, G. 2008. *Etymological Dictionary of Egyptian*, 3: *m-* (Handbuch der Orientalistik I/48, 3). Leiden: Brill

Fraenkel, S. 1962 (1886). *Die aramäische Fremdwörter im arabischen*. Hildesheim: Olms

GAG = von Soden, W. 1995. *Grundriss der akkadischen Grammatik* (Analecta Orientalia, 33). 3rd, supplemented edition, with cooperation of W. R. Mayer. Rome: Pontificium Institutum Biblicum

GKC = Kautzsch, E. 1910. *Gesenius' Hebrew Grammar.* 2nd English edn., ed. A. E. Cowley. Oxford: Oxford University Press

HALOT = Koehler, L. and W. Baumgartner. 1994-2000. *The Hebrew and Aramaic Lexicon of the Old Testament* (trans. and ed. under the supervision of M. E. J. Richardson). Leiden: Brill

Hoffner, H. 1966. A Native Akkadian Cognate to West Semitic *gbn "Cheese"? *Journal of the American Oriental Society* 86: 27-31

HSED = Orel, V. E. and O. V. Stolbova. 1995. *Hamito-Semitic Etymological Dictionary: Materials for Reconstruction*. Leiden: Brill

Hoch, J. 1994. *Semitic Words in Egyptian Texts of the New Kingdom and the Third Intermediate Period*. Princeton: Princeton University Press.

Huehnergard, J. 2005. Features of Central Semitic. In: A. Gianto (ed.), *Biblical and Oriental Essays in Memory of William L. Moran* (Biblica et Orientalia 48). Rome: Pontificium Institutum Biblicum. Pp. 152-203

Jastrow, M. 1996 (1903). *A Dictionary of the Targumim, the Talmud Babli and Yerushalmi, and the Midrashic Literature*. New York: Judaica Press

JL = Johnstone, T. M. 1981. *Jibbāli Lexicon*. Oxford: Oxford University Press

Kane, T. L. 2000. *Tigrinya-English Dictionary*. Volumes 1-2. Springfield, VA : Dunwoody Press

Kaufman, S. 1974. *The Akkadian Influences on Aramaic* (Assyriological Studies 19). Chicago: University of Chicago Press

Kogan, L. 2005. Lexicon of the Old Aramaic Inscriptions and the Historical Unity of Aramaic. In: L. Kogan et al. (eds.), *Memoriae Igor M. Diakonoff* (*Babel und Bibel* 2). Winona Lake, In.: Eisenbrauns. Pp. 513-566

Kogan, L. 2011. Proto-Semitic Lexicon. In: S. Weninger et al. (eds.), *The Semitic Languages: An International Handbook* (Handbücher zur Sprach- und Kommunikationswissenschaft 36). Berlin: De Gruyter. Pp. 179-258

Leslau, W. 1990. *Arabic Loanwords in Ethiopian Semitic*. Wiesbaden: Harrassowitz

Lester, G.B. 2011. Frequency list for Biblical Hebrew.
http://anumma.files.wordpress.com/2011/03/hebrewfrequencylimited.pdf

Lieberman, S. J. 1977. *The Sumerian Loanwords in Old Babylonian Akkadian*, I: *Prolegomena and Evidence*. Missoula, Mo.: Scholars Press

Lipiński, E. 2001. *Semitic Languages: Outline of a Comparative Grammar* (Orientalia Lovaniensia analecta 80). Second edition. Leuven: Peeters

Lonnet, A. and M.-C. Simeone-Senelle. 1997. La phonologie des langues sudarabiques modernes. In: A. S. Kaye (ed.). 1997. *Phonologies of Asia and Africa (Including the Caucasus)*, vol. 1. Winona Lake, In.: Eisenbrauns. Pp. 337-372

LSoq = Leslau, W. 1937. *Lexique Soqotri (sudarabique moderne)*. Paris: Klincksieck

Mankowski, P. V. 2000. *Akkadian Loanwords in Biblical Hebrew* (Harvard Semitic Studies 47). Winona Lake, In.: Eisenbrauns

McGovern, P.E. 2003. *Ancient Wine: The Search for the Origins of Viniculture*. Princeton: Princeton University Press

Militarev, A. 2002. The prehistory of a dispersal: The Proto-Afrasian (Afroasiatic) farming lexicon. In: *Examining the Farming/Language Dispersal Hypothesis*, ed. P. Bellwood and C. Renfrew. Cambridge: McDonald Institute of Archaeological Research, 135–150

ML = Johnstone, T. M. 1987. *Mehri Lexicon*. London: School of Oriental and African Studies

Müller, W. W. 1985. Beiträge aus dem Mehri zum etymologischen Teil des hebräichen Lexikons. In: C. Robin (ed.), *Mélanges linguistiques offerts à Maxime Rodinson par ses élèves, ses collègues et ses amis*. Paris: Geuthner. Pp. 267-278

ND = Dolgopolsky, A. 2008. *Nostratic Dictionary*. Cambridge: McDonald Institute for Archaeological Research

Rundgren, F. 1963. Hebräisch *bäṣär* "Golderz" und *ʾāmar* "sagen": Zwei Etymologien. *Orientalia*, N.S. 32: 178-183

Rundgren, F. 1990. Review of Voigt 1988. *Kratylos* 35: 179-184

SDA = Dozy, R. P. A. 1927. *Supplement aux dictionnaires arabes*. Leiden: Brill

SED 1 = Militarev, A. and L. Kogan. 2000. *Semitic Etymological Dictionary, 1: Body Parts.* Münster: Ugarit-Verlag

SED 2 = Militarev, A. and L. Kogan. 2005. *Semitic Etymological Dictionary, 2: Animal Names*. Münster: Ugarit-Verlag

Sokoloff, M. 2002a. *A Dictionary of Jewish Babylonian Aramaic of the Talmudic and Geonic periods*. Ramat-Gan: Bar Ilan University Press, Baltimore: Johns Hopkins University Press

Sokoloff, M. 2002b. *A Dictionary of Jewish Palestinian Aramaic of the Byzantine Period*. Second edition. Ramat-Gan: Bar Ilan University Press, Baltimore: Johns Hopkins University Press

Sokoloff, M. 2003. *A Dictionary of Judean Aramaic*. Ramat-Gan: Bar Ilan University Press

Sokoloff, M. 2009. *Syriac Lexicon: A Translation from the Latin, Correction, Expansion, and Update of C. Brockelmann's Lexicon Syriacum*. Winona Lake, In.: Eisenbrauns, Piscataway, NJ: Gorgias Press

Steiner, R. C. 1977. *The Case for Fricative-Laterals in Proto-Semitic* (American Oriental Series 59). New Haven: American Oriental Society

Tal, A. *A Dictionary of Samaritan Aramaic* (Handbuch der Orientalistik I/50). Leiden: Brill

Tropper, J. 2000. *Ugaritische Grammatik* (Alter Orient und Altes Testament 273). Münster: Ugarit-Verlag

Unwin, T. 1996. *Wine and the Vine: An Historical Geography of Viticulture and the Wine Trade*. London: Routledge

Voigt, R. M. 1988. *Die infirmen Verbaltypen des Arabischen und das Biradikalismus-Problem*, Wiesbaden: Steiner

WÄS = Erman, A. and W. Grapow. 1926-1950. *Wörterbuch der Ägyptischen Sprache*. Leipzig: Hinrichs
