# Supplementary material for: Statistics of Language Morphology Change: From Biconsonantal Hunters to Triconsonantal Farmers
Source: PLoS One. 2013 Dec 19;8(12):e83780. doi: 10.1371/journal.pone.0083780 (PMC3868553; doi:10.1371/journal.pone.0083780)
Supplement: Text S3 — The complete Etymological Appendix composed of the above six supporting files. (PDF) [file pone.0083780.s007.pdf]

Supporting Information for

Statistics of Language Morphology Change: From Biconsonantal Hunters to Triconsonantal Farmers

(by Noam Agmon)

Etymological Appendix

Yigal Bloch, Department of Jewish History, The Hebrew University, Jerusalem 91904, Israel.

The leftmost column of the following table includes the number of the relevant entry in the tables of the main text.

The second column from the left presents proto-words whose reconstruction is based on actual words attested in different Semitic languages. For the purposes of this reconstruction, a proto-word is a lexical unit expressing a definite semantic notion and possessing a more-or-less stable morphology – i.e., the same set of radicals (with possible metathesis of the radicals, or interchanges of homorganic consonants), and a small set of patterns (defined by the placement of vowels, prefixes and suffixes) in which those radicals are materialized. In some instances, the variety of morphologically and semantically related forms in the individual languages does not permit the reconstruction of a single form for a given proto-word. Nevertheless, the very existence of morphological and semantic similarity between the attested forms strongly suggests that they are reflexes of earlier forms belonging to a linguistic stratum that existed prior to the languages to which the attested forms belong.

Each reconstructed proto-word is classified as Proto-Semitic (PS) if its reflexes can be recognized in additional Afro-Asiatic languages beyond the Semitic family, or are attested in East Semitic (Akkadian) and at least one other Semitic language (while not an Akkadian or non-Semitic loanword in the latter). If no reflexes of a given proto-word are attested either in non-Semitic Afro-Asiatic languages or in Akkadian, that proto-word is classified as Proto-West Semitic (PWS). All proto-words of this kind quoted in the table below are attested, on the one hand, in Ethiopic or Modern South Arabian (the South Semitic languages), and on the other hand, in Hebrew, Aramaic, Ugaritic or Arabic (the Central Semitic languages). A proto-word whose reflexes are attested only in some of the latter four languages (including necessarily Arabic) is classified as Proto-Central Semitic (PCS).

The classification of languages adopted here is based on Huehnergard 2005.

Abbreviations used for Aramaic dialects:
BArm. = Biblical Aramaic
CPArm. = Christian Palestinian Aramaic
JArmTg = Jewish Aramaic of Targum Onkelos and Targum Jonathan (both composed in Palestine and edited in Babylonia in the early first millennium C.E.)
JBArm. = Jewish Babylonian Aramaic
JPArm. = Jewish Palestinian Aramaic

Mnd. = Mandaic
OArm. = Old Aramaic (9th-6th centuries B.C.E.)
OffArm = Official Aramaic (6th-4th centuries B.C.E.)
Palm. = Palmyrene (early centuries C.E.)
Sam. = Samaritan Aramaic
Syr. = Syriac

Dictionaries and lexicographical works used for individual languages and language groups:
Akkadian: *AHw*; *CAD*; *CDA*
Arabic: *AEL*; BK; Hava 1899
Aramaic: CAL; Jastrow 1996 (1903); Sokoloff 2002a; Sokoloff 2002b; Sokoloff 2003; Sokoloff 2009; Tal 2000
Geˁez: *CDG*
Hebrew: *BDB*; *HALOT*
Modern South Arabian: *JL*; *LSoq*; *ML*
Tigrinya: Kane 2000
Ugaritic: *DULAT*

For the identification of loanwords (lw.), beside the dictionaries of the specific languages, the following studies were used: Fraenkel 1962 (1886); Kaufman 1974; Leslau 1990; Lieberman 1977; Mankowski 2000. Identification of loanwords in the table without further discussion is based on the dictionaries and the abovementioned studies.

Proposals for the identification of additional words in specific languages as loanwords, or objections against existing identifications of loanwords, are discussed in the footnotes to the table.

Table S1. Etymological Appendix for Table 1.

**No.**	**Proto-word**	**Hebrew**	**Aramaic**	**Ugaritic**	**Arabic**	**Modern South Arabian**[1]	**Ethiopic**[2]	**Akkadian**[3]
1.1	*_ˀiš_, *_ˀišāt_ "fire" (PS, 2c)	_ˀēš_ "fire"	_ˀš_ (OArm.), _ˀšh_ (OffArm.), _ˀeššāˀ_, _ˀeššātāˀ_ (BArm., JArmTg., JPArm., JBArm., Syr.) "fire"	_išt_ "fire"			_ˀəsāt_ "fire"	_išātu_ "fire"
1.2	*_gir_(_r_) "fire, heat" (PS, 2c)[4]				_ǧāyirun_ "burning, intense heat inside the body"			_girru_ "fire, fire-god"
1.3	*_ˀūr_ "fire"; *_ˀār_, *_ˀurr_ "light" (PS, 2c)	_ˀūr_ "fire"; _ˀōr_ "light"	_ˀwr_ "to shine" (JPArm.)	_ar_, _ir_ "light"; _ur_ "warmth, fire"	_ˀuwārun_ "heat of fire"		_ˀarwa_ "to flame, blaze" (Tigre)	_urru_ "daytime"
1.4	*_nūr_ "light";	_nēr_ "light,	_nūr_, _nūrāˀ_ (BArm.,	_nr_ "to shine,	_nārun_ "fire";		_nār_, _nūr_ "light,	_nūru_ "light,

[1] Recent studies in the attested Epigraphic South Arabian languages suggest that "none of these languages can be the ancestor of either the Modern South Arabian languages or the Ethiopian Semitic languages" (Huehnergard 2005: 161, and see the earlier studies cited there). Since Epigraphic South Arabian languages appear to belong to the Central Semitic branch of the Semitic language family (Huehnergard 2005: 160-161), and since the Central Semitic languages are already represented in the present table by Hebrew, Aramaic, Ugaritic and Arabic, this column is restricted to Modern South Arabian languages.

[2] Unless otherwise noted, the words in this column are from Geˁez.

[3] Final mimation in Akkadian nominal forms is not marked, unless a specific syllabic spelling with final mimation is quoted.

[4] Akkadian _girru_, reflecting formally the geminate root _grr_, and Arabic _ǧāyir_, reflecting formally the hollow (II-_y_) root _ǧyr_ (BK: 361b), suggest that both these forms are extensions of the original 2c form *_gir_(_r_).

	**nār* "fire" (PS, 2c)[5]	small clay lamp"; *nîr* "light, lamp"	JArmTg., JBArm., JPArm., Syr.) "fire"; *nəhōr*, *nəhōrāʔ* (BArm., JArmTg., JBArm., JPArm.), *nūhrāʔ* (Syr.) "light"[6]	burn" (verb); *nr* "sheen, glean, lamp" (noun); *nrt* "lantern, lamp"	*nūrun* "light"		fire" (Arabic lw.)	gleam"; *nawāru* "to be(come) bright, shine"
1.5	**nabl* "flame" (PS, 2c)			*nblu* "flame"	*nablun* "ar-row"[7]		*nabal* "flame"	*nablu* "flame, flash of fire, fire-arrow"
1.6	**ʔg*, **hg* "to burn, blaze (literally or figu-				*ʔaǧǧa* "to burn, blaze (said of fire)";	*ɛhgég* "to make a big blaze, flash" (causative	*hagägä* "to smoke (said of fire), produce	*agāgu* "to be(come) angry, flare up in anger"

[5] It has been argued that in PS, **nūr* meant exclusively "light," and the use of **nūr*/**nār* for "fire" in individual languages was a later semantic development (Kogan 2011: 194-195). Indeed, in Aramaic, the use of *nūr* for "fire," instead of the earlier **ʔēš*, is a relatively late phenomenon, whose emergence can be traced in historical times (Kogan 2005: 558). However, no similar indication of a relative lateness exists for Arabic *nārun* "fire", and the verb *nr* appears to be attested in Ugaritic with the meaning "to burn" beside "to shine" (*DULAT*: 641-642; see also Del Olmo Lete 2004: 297, n. 16, 299, n. 21). It is also possible that Akkadian *tinūru* "oven," and reflexes of the same word in West Semitic (Hebrew *tannûr*, Aramaic *tannūrāʔ*, Arabic *tannūr*) are genuine Semitic derivatives from the root *nwr*, which would support the inclusion of burning, beside lighting, in the original semantic field covered by this root (see Hoch 1994: 359, n. 14). However, it is equally possible that the West Semitic words for "oven" are all loans (direct or indirect) from Akkadian *tinūru*, whereas the latter is itself a loan from Sumerian DURUN "oven" or a so-called culture word – i.e., a word common to many languages from different linguistic families over a wide geographical area, for which no specific etymology in any known language can be established (thus Mankowski 2000: 150-151; and see also Civil 1973: 174, who hesitates between the possibilities of Semitic origin, implying a loan from Akkadian, and a culture word for Sumerian DURUN).

[6] In Aramaic, the hollow roots (II-*w*/*y*) are sometimes extended to a 3c pattern through the addition of *h* as the second radical; cf. Aramaic *rhṭ* "to run," Hebrew *rwṣ*, PS **rwẓ*. This phenomenon supports the classification of the hollow roots as originally 2c.

[7] Arabic *nablun* "arrow" may reflect either the actual use of flaming arrows in ancient warfare, or a metaphorical usage of "flame" to denote an intensive use of weapons in a battle (cf. the usage of *nablu* in Akkadian in contexts related to warfare – *CAD* N/1: 26a-b, s.v. *nablu* A, 1b).

	ratively)" (PS, 2c)[8]				*hağğa* "to burn fiercely (said of fire)"	stem) (Jibbāli)	more smoke than flame, give off a strong, penetrating odor" (Tigrinya)	
1.7	**ḥr* "to be hot, burn" (PS? PWS? 2c)[9]	*ḥrh* (*ḥry*) "to be/ become hot, angry"; *ḥrr* "to burn"	*ḥry* "to be hot, glow, rake" (JBArm.); *ḥrḥr* "to set on fire" (JArmTg.)	*ḥrr* "to dry up, shrivel, burn up, catch fire"	*ḥarra* "to be hot, burn up"; *ḥarrun* "heat"	*ḥəráwrət* (Mehri) "heat, heat-spot, burnt food at the bottom of a pan"	*ḥarra*, *ḥarara* "to burn (intrans.), to be ablaze, hot, grilled, dried up"	*erēru* "to be parched (?)"; *erru* "parched (?)"[10]
1.8	**kb* "to burn, char, roast" (PS, 2c) [11]		*kbb* "to char" (JBArm., Akkadian lw.)		*kabābun* "roasted or broiled meat"	*kbb*, *qeb*(*b*) "to roast" (Soqoṭri); *qbb*		*kabābu* "to burn, scorch, char wood"

[8] The attested reflexes allow the reconstruction of both *ʔ*g* and **hg* (*ʔ*gg* and **hgg* in the 3c notation) for PS, and indicate that the original verb was, in all likelihood, intransitive. The variation *ʔ*/*h* is actually attested in the Arabic reflexes of the PS verb (*ʔağğa* vs. *hağğa*) and is explained by the fact that the consonants *ʔ* and *h* are homorganic: voiced vs. voiceless laryngeal consonants, respectively (see Lipiński 2001: §19.2).

[9] Whether this root can be reconstructed back to PS or only to PWS depends on the interpretation of the Akkadian evidence (see the following note).

[10] The meaning of the Akkadian term is not clear. *AHw*: 238b, 244a, translates the verb *erēru* "to be parched?" ("'dürr sein' ?") and the adjective *erru* "approximately 'to be parched'" ("etwa 'dürr'"); note the indications of uncertainty of the translation, specified in the original. In contrast, *CAD* E: 280a, 307b, translates *erēru* "to become moldy" and *erru* "moldy" (see further the discussion of *erēru* in *CAD* A/2: 238a-b, s.v. *arāru* C). In Sumerian-Akkadian lexical lists, the word ^{GIŠ}*erru* (where ^{GIŠ} is the determinative for the semantic category of wood) appears as equivalent to Sumerian ^{GIŠ}BÍL(gibil) and ^{GIŠ}GÍBIL "firewood, tinder" (*AHw*: 244a; *CAD* E: 307b). Wood used for fire would be more likely parched than moldy. Hence, the translation specified in *AHw* appears preferable, and is hesitantly adopted in the present table, which makes it possible to reconstruct the 2c base **ḥr* "to be hot, burn" back to PS.

[11] Hebrew and Aramaic *kbh* (*kby*) "to be quenched," and Arabic *kabā* (*kbw*) "to smoulder" (*HALOT*: 457a), are formally derived from the 3c root *kbw*. Both the roots *kbb* and *kbw* would be derived from original 2c **kb*, and a strong association between these roots is possible. However, one should be cautioned by the fact that *kbw* "to be quenched, to smoulder" cannot be traced further back than the Proto-Central Semitic (the latest common ancestor of Arabic, Canaanite – including Hebrew – and Aramaic).

						"to roast potatoes" (Mehri)		
1.9	**kwy* "to burn, cauterize" (PS, 2c)	*kwh* (*kwy*) "to burn, scorch"	*kwy* "to burn, cauterize" (CPArm., JBArm., JPArm., Syr.)		*kawā* (*kwy*) "to caute-rize, burn one's skin"			*kawû*, *kamû* "to burn (trans.), bake, roast"
1.10	*qd* "to burn, ignite"[12]	*yqd* "to burn"	*yqd* "to burn" (common)	*mqd* "scor-ched, singed"	*waqada* "to burn"	*wqd* "to put a big branch on the fire to make it burn a long time" (Mehri)		*qiādu*, *qâdu* "to ignite"
1.11	**qlw* "to roast, burn" (PS, 2c)	*qlh* (*qly*) "to roast"	*qly* "to roast, burn" (common)		*qalā* (*qlw*/*qly*) "to fry (wheat or meat)"	*qəlō* (*qlw*/*qly*) "to cook, fry, annoy, irritate" (Mehri); *qéle* (*qlw*/*qly*) "to roast, cook" (Soqoṭri)	*qalawa* "to roast, burn"	*qalû* "to burn, roast"
1.12	**qm* "to be(come) hot, burn"						*moqa* (*mwq*) "to grow hot, be warm,	*qamû* "to burn (trans.)"

[12] The verb *wqd* (> *yqd*) in West Semitic (of which Ugaritic *mqd* is a derived adjective), and the verb *qiādu* (root *qyd*) in Akkadian suggest different extensions of the original 2c root **qd*.

	(PS, 2c)[13]						become intense (heat)”; *maqaqa* “to burn, sting (e.g. medicine), burn the throat, cauterize”	
1.13	**šb*, **śb* “to burn, flare up, emit sparks” (PS, 2c)[14]	*šābîb* “spark of fire” (Aramaic lw.?)	*šəbīb*, *šəbībāˀ* “spark of fire” (BArm., JPArm., Syr., Mnd.); *šbb* “to be burned up with sparks” (JBArm.)		*šabba* “to be youthful, brisk, lively, to burn, blaze”	*śbb* “to climb, flare up (said of fire), grow up (said of youth)” (Mehri); *šebb* “to blow (upon the fire)” (Soqoṭri)	*sehbo* “slow fire”[15]	*šabābu* “to roast, burn (in transferred meanings) (?)”; *šibūbu* “spark”[16]
1.14	**lhb*	*lahab*	*lhb*		*lahaba*	*ləhēb*	*lahaba*,	*laˀbu*

---

[13] The 3c verbs *mwq*, *mqq* in Geˁez appear as extensions of original 2c **mq*, and the 3c verb *qamû* (*qmw*?) in Akkadian appears as an extension of original 2c **qm*. Comparative evidence from Western Chadic (Bolewa) suggests that the root **qm* is original, and **mq* (with further extensions) – a metathesis thereof (see *HSED*: no. 349).

[14] The evidence of Arabic and Mehri suggests that the original PS root was **śbb* (derived from 2c **śb*). However, this is not consistent with Hebrew *šābîb*, Aramaic *šəbībāˀ* “spark,” and Geˁez *sababa* “to blow up, be joyful” (if the latter is really connected with the other words cited here – cf. the following note). The Hebrew, Aramaic and Geˁez forms suggest the original root **šbb* (derived from 2c **šb*). For a discussion of the problem of etymologically related words in different Semitic languages, which include **ś* as one of the radicals in some languages and a different sibilant in a corresponding position in other languages, see Blau 1998 (1977).

[15] For a proposed etymological connection between Geˁez *sehbo* and common Semitic *šbb* “to burn, flare up,” see *CDG*: 492a. If this connection is valid, it appears that Geˁez took a different route to the extension of originally 2c base **šb* to fit the 3c pattern – by addition of *h* as the middle radical rather than by doubling the final radical.

[16] The translation “to roast, burn” for *šabābu* follows *CAD* Š/1: 2b-3a. *AHw*: 1118a-b translates “approximately ‘to glow, be parched’” (“etwa ‘glühen, verdorren’”). One way or another, the verb expresses some action connected with burning. Concerning *šibūbu* “spark,” it has been suggested that it is an Aramaic loanword (*AHw*: 1229b). However, the direction of borrowing may have been reverse: from Akkadian to Aramaic (Abraham and Sokoloff 2011: 53, no. 244). Alternatively, it is possible that both Akkadian *šibūbu* and Aramaic *šəbībāˀ* are indigenous reflexes of an original PS lexeme.

	“to burn, be inflamed, hot” (PS, 3c)	“flame”	“to be inflamed”; *lhbˀ* “flame” (JPArm.); *šlhb* “to kindle, inflame” (CPArm., JPArm., Syr.)		“to be thirsty”; *lahhaba* “to make the fire flame fiercely, or without smoke”	“hot wind” (Mehri)	*lahba* “burn (intrans.), blaze, flame, be warm, perspire”	“infectious fever (?)”[17]
1.15	*śrp “to burn” (PS, 3c)[18]	*śrp* “to burn completely”	*śrp* (OffArm.), *srp* (JBArm., JPArm., Sam.) “to burn”	*šrp* “to burn”		*śrf* “to build up sticks for fire” (Mehri)		*šarāpu* “to light a fire, burn, burn up”

[17] This is the translation proposed by *AHw*: 526b (“ein ansteckendes Fieber”). *CAD* L: 34b-35a translates *laˀbu* as “a skin disease” and “spot affected (by *laˀbu*).” However, CAD admits that the Akkadian lexical lists of synonyms explain *laˀbu* as a kind of fever for etymological reasons (*CAD* L: 35a). Thus, the etymological connection between *laˀbu* and West Semitic *lhb* “to burn, be inflamed” is supported by the evidence of the Akkadian lexical tradition.

[18] Egyptian *srf* “(to be) warm” (*WÄS* IV: 195) appears to be a cognate of this lexeme, which means that it can be traced back to PAA.

Table S2. Etymological Appendix for Table 2.

No.	Proto-word	Hebrew	Aramaic	Ugaritic	Arabic	Modern South Arabian	Ethiopic	Akkadian
2.1	**ḥaẓẓ*, **ḥiẓẓ* "arrow" (PS, 2c)	*ḥēṣ* "arrow"	*ḥṣyˀ* "arrows" (OArm.); *ḥṭˀ* "arrow" (OffArm.)	*ḥẓ* "arrow"	*ḥaẓwatun* "a small arrow"		*ḥaṣṣ* "arrow"	*ūṣu, uṣṣu* "arrow, arrowhead"
2.2	**kīs*, **kist* "small bag" (PS, 2c)[19]	*kîs* "bag, purse"	*kīs*, *kīsāˀ* "small bag, purse, fund" (JArmTg., JBArm., JPArm., Syr.)		*kīsun* "bag for money and precious stones"	*kəst* "bag" (Mehri)	*kis* "purse"	*kīsu* "leather bag for weights and silver, silver capital, treasury"
2.3	**qaš*(*t*) "bow" (PS, 2c)	*qešet* "bow"	*qšat*, *qaštāˀ*, *qeštāˀ* (common), *qšy* (Sam.) "bow"; *kšṭ* "to shoot with a bow" (Syr.)[20]	*qšt* "bow"	*qawsun* "bow"[21]		*qast* "bow"	*qaštu* "bow"
2.4	**rmy* "to throw, shoot, lay down" (PS, 2c)	*rmh* (*rmy*) "to throw, shoot"	*rmy* "to throw, set smth. down, move smth. downward"	*rmy* "to throw, shoot (?)"	*ramā* (*rmy*) "to throw, cast, shoot"		*ramaya* "to strike, hit"	*ramû* "to throw, cast, lay down"

[19] It is commonly accepted that Hebrew *kîs* and Aramaic *kīsāˀ* are loanwords from Akkadian, and that Aramaic was the source for Arabic *kīsun* and Geˁez *kis* (see, e.g., *HALOT*: 472b; *CDG*: 295b). However, Mehri *kəst* "bag" stands out due to the feminine ending *-t*, which is not attested in Akkadian *kīsu* and its alleged borrowings. Thus, whether or not the words in Hebrew, Aramaic, Arabic and Geˁez are loans from Akkadian, Mehri *kəst* appears to be a genuine cognate of Akkadian *kīsu*, and hence the lexeme in question can be reconstructed as PS.

[20] The last verb is evidently denominative from *qšat* "bow," in which the feminine ending *-t* was re-interpreted as the third radical, along with de-emphathization *q* > *k* and emphathization *t* > *ṭ*.

[21] Arabic is unique among the Semitic languages in attesting a 3c base in the noun for "bow" (*-t* in other languages was originally the feminine suffix). It has been suggested that the form *qaws* in Arabic reflects a metathesis of the root *qsw* (PS **qšw*) "to be hard," viz., "hard to bend" (Rundgren 1990: 183-184). Then, both *qaws* and *qsw* < **qšw* would be extensions of originally 2c forms.

			(common)					
2.5	**ṣd* “to prowl, roam, hunt, fish” (PS, 2c)[22]	*ṣwd* “to hunt”; *ṣayid* “game (hunted meat)”	*ṣwd* “to hunt, capture, trap” (common)	*ṣd* “to hunt, scour, traverse”	*ṣāda* (*ṣyd*) “to capture, trap, hunt, fish”	*əṣtəyūd* “to fish” (Mehri)		*ṣâdu* “to prowl, turn about, whirl”; *ṣayyādu* “stalker, stalking hunter”
2.6	**ṣīd* “provisions”; **ṣd* “to provide with food” (PS, 2c)[23]	*ṣayid*, *ṣêdâ* “food, provisions for a journey”	*ṣaydāˀ* (common), *ṣwādāˀ*, *zwādāˀ* (Syr.), *zauada* (Mnd.) “provisions”; *zəwādāˀ* “provisions (esp. for traveling), outfit for burial” (JArmTg., JBArm.); *zwd* “to provide		*zādun*, *zawādun* “provisions for traveling or for a fixed residence”; *zāda* (*zwd*) “to lay in stock provisions for traveling or for a fixed residence”	*zəwōd*, *zəwədīn* “supplies”; *azīd* (*zwd*) “to supply, provision” (Mehri)		*ṣidītu* “provisions, travel provisions”; *ṣudû* “provisions”; *ṣuddû* “to provide with food”

[22] It appears that in PS, the verb **ṣd* (**ṣwd*/**ṣyd* in the 3c notation) signified both prowling/roaming and hunting/fishing. The verb retained only the former semantic notion in Akkadian (although the noun *ṣayyādu* retained the notion of hunting), and retained only the latter semantic notion in the West Semitic languages, with the apparent exception of Ugaritic, where the meaning “to scour, traverse (a territory)” (*DULAT*: 778) seems to be a development of the original notion “to prowl, roam.”

[23] Some sort of semantic association between these words and the verb **ṣd* “to prowl, roam, hunt, fish” is possible; however, in Proto-Semitic (at the stage of tri-consonantal root morphology), the roots meaning “to prowl, roam, hunt, fish” and “to provide with food” were most likely perceived as different lexical entities, not as different aspects of the meaning of one and the same lexeme. This assumption is based on the fact that in several attested Semitic languages, these roots are treated differently, both in their use as verbs and in relation to the nouns derived from them. Thus, in Akkadian, *ṣidītu* “provisions” and *ṣuddû* “to provide with food” are derived from the 3c root *ṣdy*, whereas the verb *ṣâdu* “to prowl, turn about, whirl” and the noun *ṣayyādu* “stalker, stalking hunter” are derived from the 3c root *ṣwd*/*ṣyd*. In Arabic, the verb “to capture, trap, hunt, fish” is *ṣyd*, whereas “to lay in stock provisions” is *zwd* (it is possible that the first radical in the verb *zwd* had undergone the shift *ṣ* > *z*, from an emphatic to a voiced consonant, under the influence of the voiced dental plosive *d*). In Aramaic also, there is the verb *zwd* “to provide provisions,” and the noun *zwādāˀ* “provisions” (beside *ṣaydāˀ* and *ṣwādāˀ*), which may reflect the same shift *ṣ* > *z*; but the verb “to hunt” appears always with *ṣ* as the first radical.

			provisions" (Syr., JPArm.), "to provide outfit for burial" (JBArm.)						

Table S3. Etymological Appendix for Table 3.

No.	Proto-word	Hebrew	Aramaic	Ugaritic	Arabic	Modern South Arabian	Ethiopic	Akkadian
3.1	*ˀikkar "farmer" (PS, 3c)	*ˀikkār* "agri-cultural worker in ser-vitude, without land" (Akka-dian lw.?)	*ˀkr* "to plow, cultivate a field, bear (progeny, fruit)" (Syr.); *ˀikkārāˀ* "farmer" (JArmTg., JBArm., Syr.) (Akkadian lw.?)		*ˀakara* "to till ground, dig a cavity for water in the ground"; *ˀakkārun* "culti-vator of land" (Aramaic lws.)		*akkärä* "to renew land by plowing and sowing" (Amha-ric); *t-akärä* "to build a house and cultivate the field around it for the first time" (Chaha)[24]	*ikkaru* "farmer, plowman"[25]
3.2	*ˀasam, *ˀisint "granary, store-house" (PS, 3c)[26]	*ˀāsām* "grana-ry, store-house"	*ˀsym, ˀsymˀ* "granary" (Sam.); *ˀassān, ˀassānāˀ* "granary,	*asm* "grana-ry"				*išinnu, iššenu,* "stalk of grain"; *išittu, isittu, esittu,* "treasury,

[24] The verbs in Ethiopian languages are unlikely to be loanwords from either Aramaic or Arabic (Militarev 2002: 146).

[25] Akkadian *ikkaru* is commonly considered a loan from Sumerian ENGAR "farmer," and the corresponding words in Hebrew, Aramaic and Arabic are considered loans from Akkadian, with the Arabic words borrowed through the mediation of Aramaic (*AHw:* 369a; *CAD* I-J: 49a, 54b; *CDA*: 126a). This reconstruction is possible; however, as pointed out by Militarev, the evidence of the modern Semitic languages of Ethiopia suggests that the verbal root *ˀkr* "to cultivate land" belonged to the PS lexicon (see the preceding note). Thus, whether or not the words derived from this root in Hebrew, Aramaic and Arabic are Akkadian loans, the noun *ikkaru* in Akkadian itself appears to be genuinely Semitic, and Sumerian ENGAR appears to be a loan from Akkadian.

[26] The interchange *m*/*n* is relatively common in Semitic (Lipiński 2001: §11.7). The second radical in the Akkadian forms *išinnu* "stalk of grain" and *išittu* (< **išintu*) "treasury, storehouse" is *š*, which is not a standard correspondence for West Semitic *s*. However, interchange between *s* and *š* is attested in Akkadian – cf. the Babylonian forms of the numerals "seven" and "eight": *sebe* and *samāne* (*GAG*: §69b); these forms are reflexes of PS **šabˁ* and **ṯamāniy*, although the normal reflex of PS **š* and **ṯ* in Akkadian is *š* (see Lipiński 2001: §35.12-13). In fact, the syllabic spelling *e-si-it-tum* in a Babylonian mathematical text from the first half of the second millennium B.C.E. (*CAD* I-J: 243b, s.v. *išittu*, b) indicates the form *esittum*, with *s* as the second radical.

			storehouse” (JArmTg., JBArm., JPArm.), “provisions” (Syr.)					storehouse”
3.3	**ˁinb*, **ˁinab* “grape, fruit” (PS, 3c)[27]	*ˁēnāb* “grape(s)”	*ˁinbāˀ* (JArmTg., JPArm., JBArm.,), *ˁenbtāˀ* (Syr.), *ˁnbˀ* (CPArm.) “grape(s)”	*ġnb* “grape, grapes”[28]	*ˁinabatun* “grape”			*inbu* “fruit tree, fruit, offspring, sexual attractiveness”
3.4	**biˀr* “well, pit, cistern” (PS, 3c)[29]	*bəˀēr* “well, pit (for extraction of bitumen or storage	*byrˀ* (OArm.), *bˀr* (OffArm.), *bəˀērāˀ* (JPArm.) “well”;	*bir* “well”	*baˀara* “to dig (a well, a hole for cooking), hide, store for a time	*bayr* “well” (Mehri); *ˁébehor* “well, pit” (Soqoṭri)	*barbir*, *bərbir* “cistern, well, pit, latrine”	*būru*, *būrtu* “pit, hole, well, pond”; *berâtu* (pl.) “lagoons (?), swamps (?)”

[27] In West Semitic languages, reflexes of PS *ˁinb, *ˁinab signify uniformly grape(s). However, Akkadian *inbu* has a generic meaning “fruit, fruit tree” (*AHw*: 381b-382a; *CAD* I-J: 144a-147a); the meanings “offspring” and “sexual attractiveness” appear to be derived from that basic meaning. It is impossible to figure out whether the generic meaning “fruit” or the specific meaning “grape(s)” is more original.

[28] Ugaritic *ġnb* belongs to the limited group of words in which Ugaritic *ġ* corresponds to ˁ in other Semitic languages (see Tropper 2000: 126-127).

[29] The second radical ˀ is attested in Hebrew, Ugaritic (spelled with the sign denoting ˀ followed by *i* or a syllable-closing ˀ), as well as in some Aramaic and Arabic forms. The second radical *h* in Soqoṭri *ˁébehor* (where ˁ appears to be a morphological prefix – see *LSoq:* 295) reflects a shift from the voiced glottal ˀ to the homorganic unvoiced glottal *h*. The forms without ˀ in Aramaic and Arabic can be explained as resulting from syncopation of a syllable-closing ˀ*: *iˀ* > *ī* or **eˀ* > *ē*. Geˁez *barbir*, *bərbir* reflects reduplication of the nominal base after the radical ˀ had been lost (cf. *bētata* “to persevere, persist, last, stay,” derived from *bēta* “to spend the night, stay in a house, dwell,” which is in turn derived from the originally 3c root *byt* – see *CDG*: 113a, 116a). Akkadian *būru*, *būrtu* may be derived from PS **biˀr*, with syncopation of ˀ and vowel shift **ī* > *ū* conditioned by the labial consonant *b*: **biˀr* > **bīr* > *būr*. On the other hand, Akkadian *būru*, *būrtu* can also be derived from PS **baˀVr*, **buˀVr* “pit, hole” (see *HSED*: no. 164). It appears that the semantic notions of “well” and “pit, water-cistern” were not fully lexically distinguished in PS – hence their blending in Akkadian, in some instances in Biblical Hebrew (*beˀĕrōt ḥēmār* “bitumen pits” in Gen. 14:10 and *bəˀēr* as a pit used for grain storage in 2 Sam. 17:19), in the 1st-millennium CE Aramaic dialects (Syriac and Jewish Aramaic), and in Geˁez (where the meaning “latrine” appears to be a secondary development from “pit”). In any event, PS **biˀr* appears to have denoted water reservoirs, and may have had a more generic meaning “pit,” but probably did not refer specifically to storage pits, evidence for which is limited to reflexes of PS **biˀr* in Hebrew and Arabic only.

		of grain)"	*bēr, bērāˀ* (JArmTg., JBArm., JPArm.,), *bēˀrāˀ*, *bīrāˀ* (Syr.) "well, pit, grave"		of need"; *biˀrun, bīrun* "well"; *baˀˀārun* "digger of wells"; *baˀīratun* "a thing stored"			
3.5	**bšl* "to ripen, be cooked" (PS, 3c)[30]	*bšl* "to ripen, boil" (basic stem), "to cook" (doubled stem)	*bšl* "to ripen, be cooked" (basic stem), "to cook, boil" (doubled stem) (common)	*bšl* "to cook" (basic stem? doubled stem?)[31]	*basala* "to become strong (in taste or odor), sour (said of food)" (basic stem), "to cook unripe dates" (causative stem)	*bəhēl* "to be cooked, baked, ready" (basic stem), "to cook, prepare" (causative stem) (Mehri); *béhel* "to be cooked" (Soqoṭri)[32]	*basala* "to be cooked, be ripe" (basic stem), "to cook" (causati ve stem)	*bašālu* "to be cooked, ripen" (basic stem), "to cook" (causative stem)

[30] In all the attested Semitic languages, reflexes of PS **bšl* have the intransitive meaning "to ripen, be cooked" in the basic stem (i.e., the verbal stem that is declined without addition of consonantal prefixes or infixes, other than those expressing the grammatical person, and without lengthening of the vowels that appear between the root consonants). The transitive meaning "to cook" is reserved for the reflexes of PS **bšl* in the doubled stem (declined with the doubling of the middle radical, in all grammatical forms) and in the causative stem (declined with the addition of the consonantal prefix *š*, *h* or ˀ). The fact that no attested language uses a reflex of **bšl* in the doubled or causative stem with the meaning "to make smth. ripen" (related to floral products still in the stage of their growth), and the intransitive meaning of Arabic *basala* "to become strong, sour" (a state of food reached without a necessary human intervention) suggest that "to ripen, overripen" was the basic meaning of PS **bšl* (or its pre-PS ancestor). The meanings associated with cooking – both intransitive and transitive – appear to have resulted from later semantic developments.

[31] Comparison with other languages suggests that Ugaritic *bšl* "to cook" (transitive) is in the doubled stem. Given the relative scarcity of Ugaritic textual evidence, the absence of intransitive *bšl* "to ripen" in Ugaritic is not surprising.

[32] For the consonant shift **š* > *h* in Mehri, and in some instances, in Soqoṭri, see Lonnet and Simeone-Senelle 1997: 346-347, 361.

3.6	**buṭm*, *buṭn* "pistachio, terebinth" (PS, 3c)[33]	*bōṭnîm* (pl.) "pistachios"	*buṭmāˀ*, *buṭnāˀ* (JArmTg., JPArm., JBArm.), *beṭmətāˀ* (Syr.) "terebinth, pistachio"		*buṭmun* "terebinth, terebinth nuts (coll.)" (Aramaic lw.)		*bəṭm*, *buṭm* "terebinth tree" (Arabic lw.)	*buṭnu* "terebinth, (tree and nuts)"; *buṭuttu*, *buṭumtu*, *buṭuntu* "pistachio tree, wood, nut"
3.7	**duḫn* "millet" (PS, 3c)[34]	*dōḥan* "millet"	*duḥnāˀ* "millet" (Syr.)		*duḫnun* "millet"			*duḫnu* "millet"
3.8	**ḏaˀn* "small livestock (sheep and goats)" (PS, 3c)	*ṣōˀn*, *ṣōnê* "flocks (sheep and goats)"	*qn* (*ǧn*) (OffArm.), *ˁān, ˁānāˀ* (JPArm., Syr.) "small livestock, sheep"	*ṣin* "ewe, small livestock"	*ḍaˀnun* "sheep"			*ṣēnu*, *ṣānu*, *ṣeˀānu* "sheep and goats"
3.9	**gurn*, **garīn*, **magrān* "place for storing produce, threshing floor" (PS, 3c)	*gōren* "threshing floor"	*grnˀ* "threshing floor" (Sam., Hebrew lw.)	*grn* "threshing floor"	*ǧarana* "to grind grain vehemently"; *ǧurnun* "a stone mortar"; *ǧarīnun* "what one has ground (of grain), place where dates are dried or		*gwərn*, *gorn*, *gurn* "threshing floor"	*garānu*/*qarānu* "to store, pile up in heaps"; *magrānu*/*maqrānu* "grain pile, threshing floor"; *magrattu*/*maqrattu* "threshing floor"[36]

[33] For the interchange *m*/*n*, see above, n. 26.

[34] PS **duḫn* "millet" is suggested to derive from the Arabic verb *daḫana*, "to give smoke" (*HALOT*: 218b). However, this verb is not attested elsewhere in Semitic (*DRS*: 250), and hence is not likely to be reconstructed back to PS.

					wheat is trodden out”[35]			
3.10	**hugār* “meadow, field, arable land” (PS, 3c)[37]			*ugr* “field, soil” *ugrt* “Ugarit (toponym)”			*garh, garāht* “field, arable land, farm”; *garha* “to plow”	*ugāru* “grass-land, meadow, arable land”
3.11	**ḥaql* “field” (PS, 3c)		*ḥaqlāˀ* “field” (common)		*ḥaqlun* “field, land lacking trees, cereals at an early stage of growth”		*ḥaql* “field, plain, desert, countryside”	*eqlu* “field”
3.12	**ḥrṯ*	*ḥrš*	*ḥrt*	*ḥrṯ*	*ḥaraṯa*		*ḥarasa*	*erēšu*

[36] The cuneiform spellings of the nouns *magrānu/maqrānu*, *magrattu/maqrattu*, and of the different forms of the verb *garānu/qarānu*, do not allow to figure out whether the first radical is *g* or *q*. However, the etymological connection with the West Semitic reflexes of **gurn*, **garīn* “place for storing produce, threshing floor” appears more compelling than the connection with Arabic *qrn* “to tie, bind together,” suggested by W. von Soden (*AHw*: 902a).

[35] The Arabic forms cited here refer mostly to grinding, rather than threshing, of grain, which appears to be a later semantic development. However, one of the meanings of the noun *ǧarīnun* is “place where dates are dried,” and another meaning is “place where wheat is trodden out.” The latter two meanings fit the data from other Semitic languages, indicating that nouns derived from the root *grn* served as designations for places in which agricultural produce was stored in heaps and underwent initial processing.

[37] Geˁez **garh*, *garāht* indicate the glottal voiceless *h* as a radical. Postulating an etymological connection between the Geˁez forms, Akkadian *ugāru* and Ugaritic *ugr* (as proposed by Militarev 2002: 144) requires one to assume metathesis of the root consonants and interchange between ˀ and *h* (glottal voiced and voiceless consonants, respectively). Both assumptions are acceptable. Since Ugaritic and Akkadian belong to two different main branches of the Semitic language family (West and East Semitic, respectively), it appears that the order of the radicals and the vowel pattern attested in these two languages is closer to the form that assumedly existed in PS, and the Geˁez forms are secondary. In any event, it is unlikely that Geˁez *garh, garāht* can be etymologically connected with Arabic *qarāḥ*, *qirwāḥ* “land without trees” (as suggested in *DRS:* 184): *h* and *ḥ* are not homorganic consonants, and there is no regular sound shift in either Geˁez or Arabic, leading from one of them to the other (reservation to this effect is expressed already in *CDG*: 202b). Rather, Arabic *qarāḥ* appears to derive from the PWS root *qrḥ* “to be bald” (see *HALOT*: 1140a; *CDG*: 441a).

	"to plow" (PS, 3c)	"to plow"	"to dig, cut in pieces, incise, plow" (Syr.)	"to plow, till, farm the land"	"to plow"		"to plow"	"to seed (using a plow), to cultivate a field"
3.13	**ḫamr* "fermenting wine" (PS? PWS?, 3c)[38]	*ḥemer* "(fermenting) wine"	*ḥəmar*, *ḥamrāˀ* "wine" (common)	*ḫmr* "(fermenting?) wine"	*ḫamrun* "wine"; *ḫamara* "to become changed"	*ḥámer* "wine" (Soqoṭri)	*ḥamra* "to become sour" (Tigrinya)	*ḫammurtu* "a kind of beer" (Aramaic lw.?)[39]
3.14	**ḫimˀat* "cream, curd, butter" (PS, 3c)	*ḥemˀâ* "cream, butter"	*ḥmˀh* "curd" (Sam.); *ḥeˀwtāˀ* "butter" (Syr., Akkadian lw.)[40]	*ḫmat* "butter, curd"		*ḥámi* "butter" (Soqoṭri)		*ḫimētu*, *ḫimātu* "butter, ghee"
3.15	**kapr* "village" (PS, 3c)	*kəpār* "open village"	*kaprāˀ* "village" (JPArm., CPArm., Sam., Syr.)	*kpr* "village"	*kafrun* "village" (Aramaic lw.)			*kapru* "village in an open country, farm"
3.16	**karm*, **karān* "vine, vineyard" (PS, 3c)[41]	*kerem* "vineyard"	*krm* (OffArm.), *karmāˀ* (JPArm., JBArm.,	*krm* "vineyard"	*karmun* "vine, vineyard"	*karmáym* "mountain" (Mehri)[42]	*karm*, *kərm* "vine, vineyard"	*karānu* "wine, grapevine, grapes"

[38] If the noun *ḫammurtu* "a kind of beer" is original in Akkadian, it will be justified to reconstruct **ḫamr* as a PS lexeme. If Akkadian *ḫammurtu* is an Aramaic loanword (which is not unlikely – see the following note), one can reconstruct **ḫamr* "fermented wine" only back to PWS.

[39] This term appears only in Assyrian documents of the 1st millennium B.C.E. (*CAD* Ḫ: 69b; *AHw*: 318a), and may be an Aramaic loanword, although *AHw*: 318a suggests (with a question mark) derivation from the Akkadian verb *ḫamāru* "to become dry."

[40] Syriac *ḥeˀwtāˀ* is a loan from Akkadian *ḫimētu*, with the *m*/*w* interchange characteristic of the Babylonian dialect from the mid-2nd millennium onwards (Kaufman 1974: 55-56; cf. *GAG*: §31a).

[41] For the interchange *m*/*n*, see above, n. 26. Reconstruction of the proto-form **karān* is based on Akkadian *karānu*. However, a similar form had probably existed in West Semitic languages spoken in Canaan in the late 4th millennium B.C.E. In Egyptian, the form *k3nw* "garden, vineyard" (grammatically plural) is attested since the mid-3rd millennium B.C.E. (*WÄS* V: 106). This form is an exact parallel of Akkadian *karānu* (for the correspondence between Egyptian *3* and Semitic *r*, see *EDE* 1: 50-61).

			Syr.) "vine-yard"; *karmāʾ* (Syr.) "vine"				(Arabic lw.?)	
3.17	*labin(a)t, *libint "brick" (PS, 3c)[43]	*ləbēnâ* "brick"	*lēbnā*, *ləbēntāʾ*, *ləbēttāʾ* "brick" (common)	*lbnt* "brick"	*labinat* "brick" (Aramaic lw.?)			*libittu* "brick"
3.18	*nāqid "shep-herd" (PS, 3c)	*nōqēd* "shep-herd, sheep-breeder"	*nāqdāʾ* "shep-herd"	*nqd* "chief shep-herd"	*naqqād* "a shepherd who tends the kind of sheep called *naqd* (ugly sheep with wool of a good quality)"			*nāqidu* "herds-man"
3.19	*palg "water-course" (PS, 3c)[44]	*peleg* "artificial water channel,	*plg* "canal" (OffArm., Akkadian	*plg* "stream, canal"	*falağun*, *fuluğun* "river, rivulet,	*fəlēg* "water-course (not arti-	*falaga* "to flow, cause to flow in	*palgu* "canal, irrigation ditch"

However, Egyptian *k3nw* cannot be a genuine cognate of Akkadian *karānu*, since grapes do not naturally grow in Egypt (see McGovern 2003: 85), and it would be difficult to assume that a term for "vineyard" was inherited by Egyptian from PAA and preserved for millennia in the absence of actual vineyards in Egypt. Archaeological data indicate that viticulture was probably introduced into Egypt from Canaan in the late 4th millennium B.C.E. (McGovern 2003: 95-103), and it appears that the word *k3nw* "vineyard" was borrowed from Canaanite **karānū* (plural, base **karān*) at about the same time. Interestingly, from the 13th century B.C.E. onwards, another term for "garden, vineyard" appears in Egyptian: *k3m* (*WÄS* V: 106). This form corresponds phonologically to **karm*, which can be reconstructed for PWS based on the forms actually attested in known West Semitic languages (but whose attestations date only from the 14th century B.C.E. onwards).

[42] Etymological connection between Mehri *karmaym* "mountain" and reflexes of PS **karm*, **karān* "vineyard" (as exemplified by Hebrew *kerem*) was proposed by Müller 1985: 272. This connection may be based on the fact that the main regions of vine-growing in pre-Islamic Arabia were "hills and mountains" (Unwin 1996: 128).

[43] -(*a*)*t* in **labin*(*a*)*t*/**libint* is the feminine ending. As noted by Kaufman 1974: 66, and n. 178, "There is no compelling reason to assume that Akkadian is the origin of the common Semitic term and its related forms," and "It is, in fact, difficult to account for the derivation of the Heb[rew] form. . . from any of the Akkadian forms" (contra *HALOT*: 518a).

[44] In verbal usage, the common Semitic root *plg* (*plk* in Akkadian *palāku*) means "to split, divide." The noun **palg*, signifying a stream of water, appears to have developed from this root (see *CDG*: 159a). Although in individual Semitic languages, reflexes

		canal”	lw.)		running stream of water (for irrigation)”[45]	ficial)” (Mehri)	torrents, dig out, divide, split”; *falag* “river, brook, valley”	
3.20	**qamḥ* “vegetal food/ drink, flour” (PS, 3c)[46]	*qemaḥ* “flour”	*qamḥāʾ* “fine flour” (common)	*qmḥ* “flour”	*qamḥun* “full-grown wheat, grain of wheat”; *qamiḥa* “to eat (parched barley or wheat), drink”		*qamḥa* “to eat grain or fodder, graze”[47]	*qēmu* “flour, powder (of plants, nuts, etc.)”; *qamû*, *qemû* “to grind, pulverize”[48]
3.21	**rahṭ* “drinking trough” (PS, 3c)	*rahaṭ* “drinking trough” (Aramaic lw.?)	*rahṭāʾ* “drinking trough” (JPArm., Syr.)					*rāṭu* “water-channel, runnel”
3.22	**šʾb*	*šʾb*	*šʾb*	*šʾb*	*saʾaba*		*saʾaba*	*sâbu, sâpu*

of **palg* can signify either a natural or an artificial stream of water (or both), the digging of an artificial canal would actively divide a given plot of land in two parts. Hence, it seems more likely that PS **palg* originally designated an artificial canal, and came to be used for designating natural water streams only later.

[45] With regard to an irrigation system whose name in Arabic is derived from the root *flǧ*, compare the *Aflaj* (*ʾaflaǧ*, “channels”) system in Oman, a World Heritage site that dates ca. 500 C.E. (http://www.worldheritagesite.org/sites/aflaj.html).

[46] The data from Geʿez and Akkadian (where *qēmu* was apparently used to signify powder of different vegetal substances beside grain – see *CAD* Q: 208), as well as the meaning “to drink” attested for the Arabic verb *qamiḥa*, suggest that PS **qamḥ* had a generic meaning “vegetal food/drink.” This meaning would presumably exist in PS beside the specific meaning “flour” (in the sense of powder obtained by grinding grain), which is attested in Hebrew, Aramaic and Ugaritic.

[47] In Geʿez, there is also the verb *qamḥa* “to produce fruit,” which appears to be denominative from *qamḥ* “produce, yield, fruit, leguminous plant.” In *CDG*: 431b-432a, *qamḥa* “to produce fruit,” and the noun from which it is derived, are separated from *qamḥa* “to eat grain or fodder, graze,” and only the latter verb is etymologically connected with Hebrew *qemaḥ*, Arabic *qamḥun*, etc.

[48] The Akkadian verb *qamû*, *qemû* “to grind, pulverize” appears to be denominative from *qēmu* “flour.”

	“to draw water”	“to draw water”	“to draw out, absorb” (JBArm., JPArm.)	“to draw or carry water”	“to be satisfied with drinking”; *saˀbun* “a leather receptacle for wine, honey, butter”		“to drag, pull, follow”	“to draw water”[49]
3.23a	**šikar* “beer, intoxicating drink” (PS, 3c)	*šēkār* “beer, intoxicating drink”	*šakrāˀ* “intoxica-ing drink” (CPArm., JBArm., Syr.)		*sakarun* “wine, fermented date juice”			*šikaru*, *šikru* “beer, alcoholic drink”
3.23b	**škr* “to be(come) drunk” (PS, 3c)	*škr* “to be(come) drunk”	*škr* “to be drunk” (CPArm., JBArm., Syr.)	*škr* “to become intoxicated”	*sakira* “to become intoxicated”	*sīkər* “to be drunk” (Mehri; Arabic lw.?)[50]	*sakra* “to be drunk, intoxicated”	*šakāru* “to be(come) drunk”
3.24	**šlq* “to boil, cook” (PS, 3c)[51]	**šlq* “to boil, seethe, dissect” (post-Biblical)	**šlq* “to cook, cleanse by boiling” (JBArm., JPArm., Syr.)		*Salaqa* “to boil, cook, remove meat or hair with hot water”		*šäläqä* “to be burned, to simmer” (Tigrinya)	**salāqu* “to boil, cook”
3.25	**štl* “to plant” (PS, 3c)[52]	*štl* “to plant”	*štl* “to plant” (JBArm.,		*šatlun* “plant, sapling,	*šílil* “to plant” (Soqoṭri)[53]		*satālu* “to plant”; *šitlu*

[49] For the irregular correspondence of Akkadian *s* to West Semitic (and probably Proto-Semitic) **š*, see above, n. 26.

[50] PS **š* in Mehri normally shifted to *h*, and in some rare instances was retained (Lonnet and Simeonne-Senelle 1997: 346-347, 361). In Mehri *sīkər* “to be drunk,” the correspondence of *s* to PS **š* (where the PS consonant is assured by the evidence of Hebrew, Aramaic, Ugaritic and Akkadian) suggests that the Mehri word is a loan from Arabic.

[51] The attestation of *š* as the first radical in Hebrew, Aramaic and Tigrinya suggests that this was the first radical also in PS. Akkadian *salāqu*, with *s* as the first radical, appears to reflect an irregular sibilant correspondence (cf. above, n. 26).

[52] The correspondence between Hebrew and Aramaic *š*, on the one hand, and Arabic *š* (rather than *s*), on the other hand, is problematic. Yet, the evidence of Hebrew and Aramaic suggests that PS **š* is the original first radical of the root under

			Syr., Mnd., CPArm.); *šeteltāˀ* (Syr.), *šitlāˀ* (JBArm.), “plant”		young wood”; *šatlatun* “sapling, stalk of a plant”			“offshoot, sprout”[54]
3.26	**tibn* “straw, chaff” (PS, 3c)	*teben* “straw, chaff”	*tibnāˀ*, *tebnāˀ* “straw” (common)		*tibnun* “straw” (Aramaic lw.)			*tibnu* “straw, chaff”
3.27	**zrˁ* “to sow” (PS, 3c)[55]	*zrˁ* “to sow”; *zeraˁ* “seed, offspring”	*zrˁ* “to sow” (common)	*drˁ* “to sow, scatter”; *drˁ*, *ḏrˁ* “seed-grain, sowing”	*zaraˁa*, *ḏaraˀa* “to sow, scatter”		*zarˁa*, *zarˀa* “to sow, scatter seed”	*zarû* “to sow, scatter, winnow”; *zēru* “seed”

consideration. Perhaps Arabic *šatlun*, *šatlatun* (not attested in Classical Arabic – see *BDB*: 1060a; *SDA* I: 727a-b) are to be explained as Aramaic loans.

[53] The Soqoṭri verb appears to result from a re-analysis of the 3c root *štl*, whereby the middle radical *t* was interpreted as the morphological infix of a verbal stem, and thus excluded from the root (*LSoq*: 417).

[54] The verb *satālu* “to plant” appears only in texts composed in Assyria, viz., in the royal inscriptions of Sennacherib, 705-681 B.C.E. (see *AHw*: 1033a; *CAD* S: 197b). Hence, the spelling with a cuneiform sign indicating *s* as the first radical appears to be due to the fact that in the Assyrian dialect of the 1st millennium B.C.E. PS **š* had developed into *s* (Lipiński 2001: §15.2). The noun *šitlu* “offshoot, sprout,” attested in Babylonian lexical lists of the 2nd and the 1st millennium B.C.E., features the standard Akkadian reflex of PS **š*.

[55] The forms with *ḏ* or a reflex thereof as the first radical (i.e., Ugaritic *drˁ*, *ḏrˁ* and Arabic *ḏaraˀa*) are likely to have been generated under the influence of the PS verb **ḏrw* “to scatter, spread, winnow” and its reflexes in the attested languages (Blau 1998 [1977]: 67-68). In Akkadian, *zarû* has both the meaning “to sow” and “to winnow,” the latter being restricted to the Babylonian, and the former – almost entirely to the Assyrian dialect (see *AHw*: 1516b; *CAD* Z: 70b-71b). Although for an Akkadian verb derived from PS **zrˁ* the vowel shift **a* > *e* would be expected, its non-occurrence in *zarû* is not a conclusive argument that this verb is derived from PS **ḏrw*.

Table S4. Etymological Appendix for Table 4.

No.	Proto-word	Hebrew	Aramaic	Ugaritic	Arabic	Modern South Arabian	Ethiopic	Akkadian
4.1	**ˀmr* "to see, show, say" (PS, 3c)[56]	*ˀmr* "to say"	*ˀmr* "to say, com-mand" (common)	*ˀmr* "to look at smth."; *amr* "order, demand, word (?)"	*ˀamara* "to com-mand"	*ˀāmər* "matter, order" (Mehri)	*ˀammara* "to show, indicate, tell"	*amāru* "to see"
4.2	**hwy*, **hyy* "to be, become (smth. else), fall" (PS, 2c)	*hyh* (*hyy*) "to be"	*hyy* "to be, endure, come to pass" (common)		*hawā* (*hwy*) "to make smth. fall down"	*həwū* "to fall, swoop" (Mehri)	*tahayaya* (*hyy*) "to be free from care, disregard, overlook"	*ewû* "to change, turn into smth."
4.3	**ˁśw* "to do, work, (mis)treat smb." (PS, 2c)[57]	*ˁśh* (*ˁśy*) "to do, make"		*ˁšy* "to make, process, work, do smth. (bad)"	*ˁašā* (*ˁšw*) "to be or pretend to be blind, to treat smb. wrongly"			
4.4	**bwˀ* "to come, enter, return" (PS, 2c)	*bwˀ* "to come"		*bˀ* "to enter, come, reach"	*bāˀa* (*bwˀ*) "to return"		*boˀa* "to enter, penetrate, proceed"	*bâˀu* "to walk along, go through, come in"

[56] Assuming that the original meaning of PS **ˀmr* was "to see," and that the other meanings developed through a semantic shift ("to see" > "to show" > "to say" > "to command") seems the most economic way to account for the spectrum of the attested meanings of this verb in the Semitic languages (see Albright 1954: 229, n. 47; Rundgren 1963: 181-182). For an alternative proposal, assuming that the basic meaning of **ˀmr* in PS was "to stand out, be conspicuous, clear, bright," see *CDG*: 25b, and the earlier studies cited there.

[57] In Semitic, reflexes of the verb **ˁśw* are limited to the Central Semitic branch. However, reflexes of the 2c base **ˁś* "to do, make" are attested in Cushitic and Chadic languages (*HSED*: no. 1097). This suggests that the verb **ˁś* belongs to the PAA stratum, and therefore, **ˁśw* (extended to fit the 3c pattern) must have existed in PS.

4.5	**ntn* “to give” (PS, 2c)	*ntn* “to give”	*ntn* “to give” (common)	*ytn* “to give, direct (one’s face), raise (one’s voice)”			*natana* “to give”	*nadānu* “to give”
4.6	**hlk* “to go” (PS, 3c, but probably derived from 2c **lk*)[58]	*hlk* “to go”	*hlk* “to go, proceed, behave, die” (OffArm., BArm., JPArm., Sam.)	*hlk* “to go, run, flow, roam”	*halaka* “to perish”	*hlk* “to perish” (Soqoṭri)		*alāku* “to go”
4.7	**rˀy* “to see” (PWS, 2c)	*rˀh* (*rˀy*) “to see”			*raˀā* (*rˀy*) “to see, suppose, consider, opine”	*rāy*, *rey* “view, opinion” (Mehri, Soqoṭri); *mére* “mirror” (Soqoṭri)	*rəˀya* “to see, look at, contemplate”	
4.8	**šmˁ* “to hear, listen” (PS, 3c)	*šmˁ* “to hear, listen, obey, understand”	*šmˁ* “to hear, listen, obey, understand” (common)	*šmˁ* “to hear, listen, notice”	*samiˁa* “to hear, listen”		*samˁa* “to hear, listen, give heed, obey”	*šemû* “to hear, learn, pay attention, obey”

[58] Both Hebrew *hlk* and Akkadian *alāku* are conjugated, in some forms of the basic stem, as though the verbal root were **wlk* (GKC: §69x; *GAG*: §97n). This suggests derivation from the original 2c base **lk*, extended in different ways to fit the 3c pattern. Some measure of support for this hypothesis can be drawn from the parallel use of another verb, *hwk*, for “to go” in Aramaic (see *HALOT*: 1859b-60a). The existence of **hwk* at least as early as in PWS is supported by Geˁez *hoka* “to stir, move, agitate” (*CDG*: 220a). It seems likely that PS **hlk* was a blending of two originally separate 2c bases: **lk* and **hk*.

4.9	**dbr* "to consider the results of an affair, mediate, speak" (doubled stem) (PCS, 3c)[59]	*dbr* "to speak" (doubled stem)		*dbr* "to say, declare" (doubled stem)	*dabara* "to follow behind someone's back, follow or succeed smb."; *dabbara* "to consider the results of an affair, mediate, understand, plan"; *duburun* "back"	*adōbər* "to turn the back" (Mehri)	*tadabbara* (*dbr*) "to lie on one's back"	
4.10	**yṯb* "to sit, dwell" (PS, 2c)	*yšb* "to sit, dwell"	*ytb* "to sit, dwell" (common)	*yṯb* "to sit, down, install oneself, reside"	*waṯaba* "to leap, jump, sit down"		*ˀawsaba* "to take a wife, marry"	*wašābu* "to sit, reside, be settled"
4.11	**wḍˀ* "to go out, come forth, rise (in the sky),	*yṣˀ* "to go out, come forth"	*yˁy* "to sprout, blossom" (JArmTg. JPArm.,	*yṣˀ* "to go out, appear"		*ˀeḍa* (*ˁḍy*) "grow (said of herbs)" (Soqoṭri)	*waḍˀa*, *waṣˀa* "go out, go forth, depart, rise (said	*waṣû* "to go out, leave, rise (in the sky), sprout"

[59] The etymology of *dbr* "to speak" in Hebrew and Ugaritic (in the doubled stem) poses a problem. Apparently, the verb **dbr* in PWS signified different actions connected with one's back or rear side (as can be seen from the evidence of Geˁez, Mehri and the basic stem in Arabic). From this basic meaning, through a semantic shift ("rear side" > "later phase of an affair" > "outcome"), the verb **dbr* acquired the meaning "to consider the results of an affair, mediate, plan," as attested in the double stem in Arabic. "To speak" in Hebrew and Ugaritic (in the double stem) appears to be a further extension of this meaning, based on the notion of speech as an instrument of interpersonal mediation. The meaning of *dbr* connected with mediation, planning and speech is attested only in Central Semitic languages, hence the proto-verb **dbr* with this meaning can be reconstructed only on the level of PCS.

	sprout" (PS, 2c)		Syr.)				of sun)"	
4.12	**ṯwb* "to turn back, return" (PWS, 2c)	*šwb* "to turn back, return, repent"	*twb* "to return, turn about, regret" (common, written *šwb* before ca. 500 BCE)	*ṯb* "to turn around, return, repeat, answer"	*ṯaba* (*ṯwb*) "to return, repent, return to one's health"	*ṯwūb* "to requite smb." (Mehri)	*soba* "to turn, turn around, wrap around"	
4.13	**lqḥ* "to take" (PS, 3c)	*lqḥ* "to take, seize, accept"	*lqḥ* "to take, accept (favo-rably, collect)"	*lqḥ* "to take hold of smth., take, grasp, obtain"	*laqiḥa* "to conceive (said of a female), become fecund-dated (said of land)"	*lūqəḥ, līqəḥ* "to conceive (said of a she-camel)" (Mehri)	*laqqəḥa* "to lend"	*leqû* "to take, accept, assume responsi-bility"
4.14	**ydʕ* "to know" (PS, 2c)	*ydʕ* "to notice, learn, know (also sexu-ally)"	*ydʕ* "to know, perceive, cohabit with a woman" (common)	*ydʕ* "to know, reco-gnize, distin-guish"		*wīda* (*wdʕ*) "to know" (Mehri);	*ʔaydəʕa* "to make know, inform, announ-ce"	*idû, edû, wadû* "to know, be experi-enced, familiar with smth."
4.15	**ʕlw* "to ascend" (PS, 2c)	*ʕlh* (*ʕly*) "to go up"	*ʕly* "raise, exalt, bring back, evaluate" (doubled stem) (JArmTg., JBArm.,	*ʕly* "to go up, rise, attack"	*ʕalā* (*ʕlw*) "to be(come) high, elevated, ascend"	*ʔālēw* (*ʕlw*) "at the top" (Mehri); *ʕélhe* "high" (Soqoṭri)		*elû* "to up (to a higher ground or a more important locality, rise, grow, emerge"

			JPArm., Syr.)					
4.16	**šlḥ* “to stretch out, throw, send” (PS, 3c)	*šlḥ* “to stretch out, let free, send”	*šlḥ* “to send, stretch out, put out” (common)	*šlḥ* “to stretch, throw, send, give”		*hlˁ* (causative *šalaḥ*) “to cast a shadow” (Soqoṭri)		*šalû* “to whirl up, kick up dust, toss, spit, shoot arrows”[60]
4.17	**mwt* “to die” (PS, 2c)	*mwt* “to die”	*mwt* “to die” (common)	*mt* “to die, remain immobi-lized, lie aground (?)”	*māta* (*mwt*) “to die”	*mōt* “to die” (Mehri)	*mota* “to die”	*mâtu* “to die”
4.18	**ˀkl* “to eat” (PS, 3c)	*ˀkl* “to eat, devour”	*ˀkl* “to eat, consume” (common)	*ˀkl* “to eat, devour, consu-me, make use of smth.”	*ˀakala* “to swallow food after chewing, eat, consume”		*ˀəkl* “food, bread, grain, fodder”	*akālu* “to eat, consume, provide for oneself”
4.19	**qrˀ* “to call, shout, invite” (PS, 3c)	*qrˀ* “to call, give a name, shout, announ-ce”	*qry* “to call, read, cry out, proclaim” (common)	*qrˀ* “to call, shout, invite, invoke”	*qaraˀa* “to collect, put together, read and recite a book”			*qerû* “to invite, take along, lead away”
4.20	**nśˀ* “to rise, be(come) elevated, lift up,	*nśˀ* “to carry, lift up, raise”	*nśˀ* “to lift up, take away” (OArm.,	*nšˀ* “to raise, lift, load with	*našaˀa* “to live, rise, become elevated,	*miśiˀoh* “bucket”	*naśˀa*, *nasˀa* “to take, partake, receive,	*našû* “to lift, take up, wear, carry,

[60] For a proposed etymological connection between Akkadian *šalû* “to whirl up, kick up dust,” etc., and West Semitic *šlḥ* “to stretch out, throw, send,” see *DULAT*: 816.

	wear,<br>carry”<br>(PS, 3c)		OffArm.,<br>BArm.,<br>JPArm.)	smth.,<br>wear”	grow up”		capture,<br>pick up,<br>raise,<br>carry off”	bring,<br>accept,<br>receive”

References

Abraham, K. and Sokoloff, M. 2011. Aramaic Loanwords in Akkadian – A Reassessment of the Proposals. *Archiv für Orientforschung* 52: 22-76

AEL = Lane, E. W. 1968 (1863-1893). *An Arabic-English Lexicon*. Beirut: Librarie du Liban

AHw = von Soden, W. 1956-1981. *Akkadisches Handwörterbuch*. Wiesbaden: Harrassowitz

Albright, W. F. 1954. Northwest-Semitic Names in a List of Egyptian Slaves from the Eighteenth Century B.C. *Journal of the American Oriental Society* 74: 222-233

BDB = Brown, F., S. R. Driver, and C. A. Briggs. 1906. *A Hebrew and English Lexicon of the Old Testament*. Boston: Houghton, Mifflin & Co.

BK = de Biberstein Kazimirski, A. 1846. *Dictionnaire arabe-français contenant toutes les racines de la langue arabe, leurs derives, tant dans l'idiome vulgaire que dans l'idiome littéral, ainsi que les dialects d'Alger et de Maroc*. Paris: Barrois

Blau, J. 1998 (1977). "Weak" Phonetic Change and the Hebrew *śîn*. In: J. Blau, *Topics in Hebrew and Semitic Linguistics*. Jerusalem: Magnes. Pp. 50-103

CAD = Gelb, I. J. *et al.* (eds.). 1956-2011. *The Assyrian Dictionary of the Oriental Institute of the University of Chicago*. Chicago: Oriental Institute

CAL = Kaufman, S. et al. n.d. *Comprehensive Aramaic Lexicon*. Cincinnati: Hebrew Union College. Available online: http://cal1.cn.huc.edu/

CDG = Leslau, W. 1987. *Comparative Dictionary of Geʿez (Classical Ethiopic)*. Wiesbaden: Harrassowitz

Civil, M. 1973. Notes on Sumerian Lexicography, II. *Journal of Cuneiform Studies* 25: 171-177

Del Olmo Lete, G. 2004. *Canaanite Religion according to the Liturgical Texts of Ugarit* (tr. W. G. E. Watson). Winona Lake, In.: Eisenbrauns

Diakonoff, I. 1998. Earliest Semitic society linguistic data. *Journal of Semitic Studies* 43: 209-219

DRS = Cohen, D. (continued by J. Lentin, F. Bron and A. Lonnet). 1970-2012. *Dictionnaire des racines sémitiques ou attestées dans les langues sémitiques.* Fascicules 1-10. Leuven: Peeters

DULAT = del Olmo Lete, G. and J. Sanmartín. 2003. *A Dictionary of the Ugaritic Language in the Alphabetic Tradition* (Handbuch der Orientalistik I/67). Tr. by W. G. E. Watson. Leiden: Brill

EDE 1 = Takács, G. 1999. *Etymological Dictionary of Egyptian*, 1: *A Phonological Introduction* (Handbuch der Orientalistik I/48, 1). Leiden: Brill

EDE 2 = Takács, G. 2001. *Etymological Dictionary of Egyptian*, 2: *b-, p-, f-* (Handbuch der Orientalistik I/48, 2). Leiden: Brill

EDE 3 = Takács, G. 2008. *Etymological Dictionary of Egyptian*, 3: *m-* (Handbuch der Orientalistik I/48, 3). Leiden: Brill

Fraenkel, S. 1962 (1886). *Die aramäische Fremdwörter im arabischen*. Hildesheim: Olms

GAG = von Soden, W. 1995. *Grundriss der akkadischen Grammatik* (Analecta Orientalia, 33). 3rd, supplemented edition, with cooperation of W. R. Mayer. Rome: Pontificium Institutum Biblicum

GKC = Kautzsch, E. 1910. *Gesenius' Hebrew Grammar*. 2nd English edn., ed. A. E. Cowley. Oxford: Oxford University Press

HALOT = Koehler, L. and W. Baumgartner. 1994-2000. *The Hebrew and Aramaic Lexicon of the Old Testament* (trans. and ed. under the supervision of M. E. J. Richardson). Leiden: Brill

Hoffner, H. 1966. A Native Akkadian Cognate to West Semitic *gbn "Cheese"? *Journal of the American Oriental Society* 86: 27-31

HSED = Orel, V. E. and O. V. Stolbova. 1995. *Hamito-Semitic Etymological Dictionary: Materials for Reconstruction.* Leiden: Brill

Hoch, J. 1994. *Semitic Words in Egyptian Texts of the New Kingdom and the Third Intermediate Period.* Princeton: Princeton University Press.

Huehnergard, J. 2005. Features of Central Semitic. In: A. Gianto (ed.), *Biblical and Oriental Essays in Memory of William L. Moran* (Biblica et Orientalia 48). Rome: Pontificium Institutum Biblicum. Pp. 152-203

Jastrow, M. 1996 (1903). *A Dictionary of the Targumim, the Talmud Babli and Yerushalmi, and the Midrashic Literature.* New York: Judaica Press

JL = Johnstone, T. M. 1981. *Jibbāli Lexicon.* Oxford: Oxford University Press

Kane, T. L. 2000. *Tigrinya-English Dictionary*. Volumes 1-2. Springfield, VA : Dunwoody Press

Kaufman, S. 1974. *The Akkadian Influences on Aramaic* (Assyriological Studies 19). Chicago: University of Chicago Press

Kogan, L. 2005. Lexicon of the Old Aramaic Inscriptions and the Historical Unity of Aramaic. In: L. Kogan et al. (eds.), *Memoriae Igor M. Diakonoff* (*Babel und Bibel* 2). Winona Lake, In.: Eisenbrauns. Pp. 513-566

Kogan, L. 2011. Proto-Semitic Lexicon. In: S. Weninger et al. (eds.), *The Semitic Languages: An International Handbook* (Handbücher zur Sprach- und Kommunikationswissenschaft 36). Berlin: De Gruyter. Pp. 179-258

Leslau, W. 1990. *Arabic Loanwords in Ethiopian Semitic*. Wiesbaden: Harrassowitz

Lieberman, S. J. 1977. *The Sumerian Loanwords in Old Babylonian Akkadian*, I: *Prolegomena and Evidence*. Missoula, Mo.: Scholars Press

Lipiński, E. 2001. *Semitic Languages: Outline of a Comparative Grammar* (Orientalia Lovaniensia analecta 80). Second edition. Leuven: Peeters

Lonnet, A. and M.-C. Simeone-Senelle. 1997. La phonologie des langues sudarabiques modernes. In: A. S. Kaye (ed.). 1997. *Phonologies of Asia and Africa (Including the Caucasus)*, vol. 1. Winona Lake, In.: Eisenbrauns. Pp. 337-372

LSoq = Leslau, W. 1937. *Lexique Soqotri (sudarabique moderne)*. Paris: Klincksieck

Mankowski, P. V. 2000. *Akkadian Loanwords in Biblical Hebrew* (Harvard Semitic Studies 47). Winona Lake, In.: Eisenbrauns

McGovern, P.E. 2003. *Ancient Wine: The Search for the Origins of Viniculture*. Princeton: Princeton University Press

Militarev, A. 2002. The prehistory of a dispersal: The Proto-Afrasian (Afroasiatic) farming lexicon. In: *Examining the Farming/Language Dispersal Hypothesis*, ed. P. Bellwood and C. Renfrew. Cambridge: McDonald Institute of Archaeological Research, 135–150

ML = Johnstone, T. M. 1987. *Mehri Lexicon.* London: School of Oriental and African Studies

Müller, W. W. 1985. Beiträge aus dem Mehri zum etymologischen Teil des hebräichen Lexikons. In: C. Robin (ed.), *Mélanges linguistiques offerts à Maxime Rodinson par ses élèves, ses collègues et ses amis*. Paris: Geuthner. Pp. 267-278

ND = Dolgopolsky, A. 2008. *Nostratic Dictionary*. Cambridge: McDonald Institute for Archaeological Research

Rundgren, F. 1963. Hebräisch *bāṣār* "Golderz" und *ʔāmar* "sagen": Zwei Etymologien. *Orientalia*, N.S. 32: 178-183

Rundgren, F. 1990. Review of Voigt 1988. *Kratylos* 35: 179-184

SDA = Dozy, R. P. A. 1927. *Supplement aux dictionnaires arabes*. Leiden: Brill

SED 1 = Militarev, A. and L. Kogan. 2000. *Semitic Etymological Dictionary, 1: Body Parts.* Münster: Ugarit-Verlag

SED 2 = Militarev, A. and L. Kogan. 2005. *Semitic Etymological Dictionary, 2: Animal Names*. Münster: Ugarit-Verlag

Sokoloff, M. 2002a. *A Dictionary of Jewish Babylonian Aramaic of the Talmudic and Geonic periods*. Ramat-Gan: Bar Ilan University Press, Baltimore: Johns Hopkins University Press

Sokoloff, M. 2002b. *A Dictionary of Jewish Palestinian Aramaic of the Byzantine Period*. Second edition. Ramat-Gan: Bar Ilan University Press, Baltimore: Johns Hopkins University Press

Sokoloff, M. 2003. *A Dictionary of Judean Aramaic*. Ramat-Gan: Bar Ilan University Press

Sokoloff, M. 2009. *Syriac Lexicon: A Translation from the Latin, Correction, Expansion, and Update of C. Brockelmann's Lexicon Syriacum*. Winona Lake, In.: Eisenbrauns, Piscataway, NJ: Gorgias Press

Steiner, R. C. 1977. *The Case for Fricative-Laterals in Proto-Semitic* (American Oriental Series 59). New Haven: American Oriental Society

Tal, A. *A Dictionary of Samaritan Aramaic* (Handbuch der Orientalistik I/50). Leiden: Brill

Tropper, J. 2000. *Ugaritische Grammatik* (Alter Orient und Altes Testament 273). Münster: Ugarit-Verlag

Unwin, T. 1996. *Wine and the Vine: An Historical Geography of Viticulture and the Wine Trade*. London: Routledge

Voigt, R. M. 1988. *Die infirmen Verbaltypen des Arabischen und das Biradikalismus-Problem*, Wiesbaden: Steiner

WÄS = Erman, A. and W. Grapow. 1926-1950. *Wörterbuch der Ägyptischen Sprache*. Leipzig: Hinrichs
