# Supplementary material for: Statistics of Language Morphology Change: From Biconsonantal Hunters to Triconsonantal Farmers
Source: PLoS One. 2013 Dec 19;8(12):e83780. doi: 10.1371/journal.pone.0083780 (PMC3868553; doi:10.1371/journal.pone.0083780)
Supplement: Table S5 — Ranking of “weak” (originally 2c) verbal roots in the Hebrew Bible by frequency. The 2c roots were extracted from the list in Ref. 31 according to the "broad definition" in the Methods section. (PDF) [file pone.0083780.s008.pdf]

Table S5. Ranking of "weak" (originally 2c) verbs in the Hebrew Bible by frequency (by Yigal Bloch, based on Lester 2011)

**Rank**	**Root**	**Meaning**	**Number of occurrences In the Hebrew Bible**
1	*hyh*	to be	3576
2	*ˁśh* (1)*	to do, make	2632
3	*bwˀ*	to come	2579
4	*ntn*	to give, put, set	2014
5	*hlk***	to walk, go	1554
6	*rˀh*	to see	1310
7	*yšb*	to sit, dwell	1087
8	*yṣˀ*	to go out	1075
9	*šwb*	to return	1075
10	*ydˁ*	to know	952
11	*ˁlh*	to go up	894
12	*mwt*	to die	845
13	*nśˀ*	to lift, carry, take	658
14	*qwm*	to arise, stand	627
15	*śym*	to put, set	588
16	*nkh*	to smite	501
17	*ṣwh*	to command	496
18	*yld*	to bear, beget	495
19	*npl*	to fall	435
20	*yrd*	to go down	382
21	*bnh*	to build	377
22	*ngd*	to tell, declare	371
23	*yrˀ* (1)	to fear	318
24	*ˁnh* (1)	to answer	317
25	*swr*	to turn aside	298
26	*ḥyh*	to live	287
27	*yrš*	to possess, inherit, dispossess	232
28	*kwm*	to be firm, establish, prepare	219
29	*šth*	to drink	217
30	*nṭh*	to stretch out	216
31	*ysp*	to add, do again	215
32	*nṣl*	to take away, deliver	213
33	*klh*	to be complete, finished	208

* A number in parentheses after a verbal root serves to distinguish between homonymous 3c roots.

** For the classification of the verb hlk "to walk, go" as originally 2c (**lk*), see n. 58 to the Etymological Appendix.

34	*rwm*	to be high, exalted	195
35	*ykl*	to be able	194
36	*glh*	to uncover, remove<br>go into exile	187
37	*yšʕ*	to deliver, save	178
38	*rbh* (1)	to be many, great	178
39	*ḥwh* (2)	to bow, worship	173
40	*byn*	to understand	171
41	*rʕh* (1)	to shepherd, pasture, feed	167
42	*sbb*	to go around	163
43	*nws*	to flee	160
44	*ksh*	to cover	153
45	*ngʕ*	to touch, strike	150
46	*hll* (2)	to praise	146
47	*nsʕ*	to pull out, set out, journey	146
48	*ḥnh* (1)	to camp	143
49	*ḥll* (1)	to profane, begin	135
50	*nwḥ* (1)	to rest	134
51	*pnh*	to turn	134
52	*bwš* (1)	to be ashamed	125
53	*ngš*	to approach	125
54	*yṭb*	to be good, pleasing	117
55	*nbʔ*	to prophesy	115
56	*bkh*	to weep	114
57	*ydh* (2)	to praise, confess	111
58	*nḥm*	to comfort, relent	108
59	*ytr*	to remain over	106
60	*rwṣ*	to run	104
61	*rʕʕ* (1)	to do evil	97
62	*ḥrh* (1)	to burn, be angry	93
63	*šmm*	to be desolate, appalled	92
64	*šyr*	to sing	88
65	*šyt*	to put, set	88
66	*qnh*	to buy	85
67	*pll*	to pray	84
68	*gwr* (1)	to sojourn, dwell	83
69	*qll*	to curse, be easy, light, swift	82
70	*ʕwr* (2)	to arouse, stir up, uncover	80
71	*ʕnh* (2)	to oppress, humiliate,<br>be afflicted	79

72	*ḥnn* (1)	to show favor	77
73	*ḥlh*	to be sick, entreat	75
74	*nṣb* (1)	to stand	74
75	*lyn*	to lodge, stay overnight	70
76	*nbṭ*	to look	70
77	*ryb*	to strive, contend	70
78	*pwṣ*	to scatter, overflow	66
79	*nṣḥ*	to direct (music), oversee	65
80	*tmm*	to be complete	64
81	*ʾrr*	to curse	63
82	*nṣr*	to watch, keep	62
83	*šqh*	to cause to drink	62
84	*znh* (1)	to be a harlot	60
85	*pdh*	to redeem	60
86	*ybš*	to be dry, wither	59
87	*ykḥ*	to decide, reprove	59
88	*nḥl*	to inherit, possess	59
89	*nṭʿ*	to plant	59
90	*šdd*	to devastate	58
91	*yʿṣ*	to counsel	57
92	*ṣrr* (1)	to bind, restrict, distress	57
93	*ḥzh*	to see	55
94	*ʾbh*	to be willing	54
95	*ḥtt*	to be dismayed	53
96	*yṣq*	to pour, cast	53
97	*rnn*	to sing for joy	53
98	*mdd*	to measure	52
99	*ndḥ* (1)	to banish, drive away	51
100	*tʿh*	to err	51
101	*nkr*	to recognize, disguise, treat as foreign	50
102	*nśg*	to reach, overtake	50
103	*ngp*	to strike	49
104	*yṣb*	to stand	48
105	*rṣh* (1)	to accept, be pleased with	48
106	*gyl*	to rejoice	47
107	*ḥyl* (1)	to writhe, travail, wait	47
108	*prr* (1)	to break	47
109	*ṣph* (2)	to overlay	47
110	*qwh* (1)	to wait for, hope	47

111	*šbh*	to take captive	47
112	*yrh* (3)	to teach, show	46
113	*rph*	to sink, fail, leave alone	46
114	*yṣr*	to form, plan, make pottery	45
115	*bll*	to mix, give feed	44
116	*mrh*	to rebel	44
117	*nqh*	to be unpunished, clean, free	44
118	*rwʕ*	to shout	44
119	*ʔwr*	to be light	43
120	*bzz*	to plunder	43
121	*hrh*	to conceive	43
122	*yḥl*	to wait, hope	43
123	*bzh*	to despise	42
124	*zwb*	to flow	42
125	*ysd* (1)	to lay a foundation, establish	42
126	*nwʕ*	to shake, wander	42
127	*ntṣ*	to break down	42
128	*mwṭ*	to totter, shake	41
129	*ysr* (1)	to instruct, warn, rebuke	40
130	*ntš*	to forsake	40
131	*ʕwd* (2)	to warn, testify	40
132	*nḥh* (1)	to lead	39
133	*zrh* (1)	to scatter	38
134	*kwl*	to provide, contain	38
135	*ṣph* (1)	to watch	38
136	*ḥsh*	to take refuge	37
137	*nsh*	to test, try	36
138	*nqm*	to avenge	35
139	*hmh*	to roar	34
140	*mḥh* (1)	to wipe out	34
141	*nwp* (1)	to wave	34
142	*yhb*	to give, come	33
143	*ṣwr* (1)	to besiege, bind	32
144	*yll*	to wail	31
145	*mwl* (1)	to circumcise	31
146	*nʔp*	to commit adultery	31
147	*ndr*	to vow	31
148	*nšq* (1)	to kiss	31
149	*ʔwh*	to desire	30
150	*dmh* (1)	to be like, intend	30

151	*nhg* (1)	to drive, lead	30
152	*yʕd*	to appoint, meet	29
153	*yrh* (1)	to shoot, throw	29
154	*prh*	to bear fruit	29
155	*mnh*	to number, count, appoint	28
156	*ndd*	to flee, wander	28
157	*qšh*	to be hard	28
158	*tlh*	to hang	28
159	*yṣt*	to kindle, burn	27
160	*yšr*	to be smooth, straight, right	27
161	*ntq*	to tear off, lure away	27
162	*ʕwp* (1)	to fly	27
163	*pth* (1)	to be simple, entice, deceive	27
164	*śwś*	to rejoice	27
165	*ygʕ*	to toil, be weary	26
166	*ṣrr* (2)	to be hostile	26
167	*hgh* (1)	to mediate, moan, speak	25
168	*nwd*	to move to and fro, wander, mourn	25
169	*nsk* (1)	to pour out	25
170	*gwʕ*	to die	24
171	*ḥws*	to pity	24
172	*kbh*	to quench	24
173	*nʔṣ*	to despise	24
174	*nzh*	to sprinkle	24
175	*swg* (1)	to turn back	24
176	*rwš*	to be poor	24
177	*twr*	to explore, spy	24
178	*dyn*	to judge	23
179	*yʕl*	to profit	23
180	*ngś*	to oppress	23
181	*ḥmm*	to warm	22
182	*ṭwb*	to be pleasing, good	22
183	*mss*	to melt	22
184	*pśh*	to spread	22
185	*qyṣ* (2)	to awake	22
186	*qrh*	to befall, happen	22
187	*rbb* (1)	to be many, great	22
188	*rdh* (1)	to rule	22
189	*ʔth*	to come, bring	21

190	*mwš* (2)	to depart	21
191	*ntk*	to pour out	21
192	*ntš*	to uproot	21
193	*ṣwm*	to fast	21
194	*šgh*	to err	21
195	*šwʕ*	to cry for help	21
196	*yḥś*	to be enrolled by genealogy	20
197	*śyḥ*	to meditate, speak	20
198	*dmm* (1)	to be silent, be dumb	19
199	*ḥqq*	to inscribe, decree	19
200	*ynh*	to oppress, wrong	19
201	*lʔh*	to be weary	19
202	*nbl* (1)	to fade, wither, droop	19
203	*nqb*	to designate, curse, pierce	19
204	*ʕll*	to act severely, glean	19
205	*ryq*	to empty	19
206	*rṣṣ*	to crush	19
207	*gll*	to roll	18
208	*ḥwš* (1)	to hasten	18
209	*yʔl* (1)	to undertake, be willing	18
210	*ybl*	to carry	18
211	*lwn* (1)	to grumble	18
212	*nṭp*	to drop	18
213	*npṣ* (1)	to shatter	18
214	*nšʔ* (1)	to be in debt	18
215	*swt*	to incite, mislead	18
216	*yṣg*	to set, place	17
217	*ktt*	to beat, crush	17
218	*mwg*	to melt, waver	17
219	*ndb*	to freely offer	17
220	*nqp* (2)	to surround	17
221	*sph*	to sweep away	17
222	*srr*	to be rebellious, stubborn	17
223	*ʕwh*	to twist, commit iniquity	17
224	*ṣwd*	to hunt	17
225	*šḥḥ*	to bow down, be humble	17
226	*blh*	to wear out	16
227	*dwš*	to thresh, trample	16
228	*ḥgg*	to celebrate	16
229	*ḥšh*	to be silent, still	16

230	*ynq*	to suck	16
231	*yšn* (1)	to sleep	16
232	*mrr*	to be bitter	16
233	*skk* (1)	to cover	16
234	*ʿnh* (4)	to sing, cry	16
235	*šwh* (1)	to be like, compare	16
236	*gzz*	to shear	15
237	*grh*	to provoke	15
238	*hll* (3)	to be mad, boast	15
239	*ḥṣh*	to divide	15
240	*ngn*	to play music	15
241	*ʿrh*	to be naked, empty	15
242	*pṣh*	to open	15
243	*qdd*	to bow down	15
244	*šwr* (1)	to behold	15
245	*bwz*	to despise	14
246	*brr* (1)	to purify, purge	14
247	*zmm*	to purpose	14
248	*ḥkh*	to wait	14
249	*ṭwl*	to hurl	14
250	*krh* (1)	to dig	14
251	*lwh* (2)	to borrow	14
252	*mwr*	to change	14
253	*nšʾ* (2)	to deceive	14
254	*qbb*	to curse	14
255	*qṣṣ*	to cut off	14
256	*rwh*	to be saturated, drink	14
257	*rwḥ*	to be wide, get relief, smell	14
258	*šll* (2)	to spoil, plunder	14
259	*šnh* (1)	to change	14
260	*ʾph*	to bake	13
261	*dqq*	to crush	13
262	*hmm* (1)	to make a noise, confuse	13
263	*ʿṭh* (1)	to wrap, cover	13
264	*šwṭ* (1)	to go to and fro, roam, whip, despise	13
265	*bws*	to trample	12
266	*dmh* (3)	to be ruined, cut off	12
267	*ḥph*	to cover	12
268	*lwh* (1)	to join	12

269	*npḥ*	to breathe	12
270	*sll*	to build up	12
271	*ʕwt*	to be bent, pervert, sustain	12
272	*ṣwq* (1)	to oppress	12
273	*šʕh*	to gaze	12
274	*ṭwḥ*	to plaster	11
275	*yqṣ*	to awake	11
276	*yqr*	to be precious	11
277	*nbʕ*	to flow, utter, pour out	11
278	*ngḥ*	to push, gore	11
279	*nzl*	to flow	11
280	*nḥš*	to practice divination	11
281	*nʕr* (2)	to shake	11
282	*nšk* (1)	to bite	11
283	*ʕzz*	to be strong	11
284	*ʕnn*	to practice witchcraft, cloud	11
285	*šsh*	to plunder	11
286	*ʔwṣ*	to hasten, urge, be narrow	10
287	*gwr* (3)	to fear	10
288	*zyd*	to boil, act presumptuously	10
289	*ḥwl*	to whirl, dance, writhe	10
290	*mqq*	to rot	10
291	*nhl*	to guide, lead	10
292	*nzr*	to dedicate, consecrate	10
293	*nḥt*	to descend	10
294	*swk* (2)	to anoint	10
295	*pwḥ* (2)	to testify	10
