# Supplementary material for: Statistics of Language Morphology Change: From Biconsonantal Hunters to Triconsonantal Farmers
Source: PLoS One. 2013 Dec 19;8(12):e83780. doi: 10.1371/journal.pone.0083780 (PMC3868553; doi:10.1371/journal.pone.0083780)
Supplement: Table S6 — Transliteration: Proto-Semitic consonant phonemes with their Hebrew and Arabic equivalents. (PDF) [file pone.0083780.s009.pdf]

Table S6. Proto-Semitic (PS) consonant phonemes with their Hebrew and Arabic equivalents.

ṯ	***t***	***ś***	***š***	***r***	***q***	***ḍ***	***ṣ***	***p***	***ġ***	***ʿ***	***s***	***n***	***m***	***l***	***k***	***y***	***ẓ***	***ṭ***	***ḫ***	***ḥ***	***z***	***w***	***h***	***ḏ***	***d***	***g***	***b***	***ʾ***	**PS**
–	ת	שׂ	שׁ	ר	ק	–	צ	פ	–	ע	ס	נ	מ	ל	כ	י	–	ט	–	ח	ז	ו	ה	–	ד	ג	ב	א	**Heb.**
ث	ت	–	ش	ر	ق	ض	ص	ف	غ	ع	س	ن	م	ل	ك	ي	ظ	ط	خ	ح	ز	و	ه	ذ	د	ج	ب	أ	**Arabic**
